# Time-Series Field Phenotyping of Soybean Growth Analysis by Combining Multimodal Deep Learning and Dynamic Modeling

**DOI:** 10.34133/plantphenomics.0158

**Published:** 2024-03-20

**Authors:** Hui Yu, Lin Weng, Songquan Wu, Jingjing He, Yilin Yuan, Jun Wang, Xiaogang Xu, Xianzhong Feng

**Affiliations:** ^1^ Key Laboratory of Soybean Molecular Design Breeding, State Key Laboratory of Black Soils Conservation and Utilization, Northeast Institute of Geography and Agroecology, Chinese Academy of Sciences, Changchun 130102, China.; ^2^ Zhejiang Lab, Hangzhou 310012, China.; ^3^ Yanbian University, Yanji 133002, China.

## Abstract

The rate of soybean canopy establishment largely determines photoperiodic sensitivity, subsequently influencing yield potential. However, assessing the rate of soybean canopy development in large-scale field breeding trials is both laborious and time-consuming. High-throughput phenotyping methods based on unmanned aerial vehicle (UAV) systems can be used to monitor and quantitatively describe the development of soybean canopies for different genotypes. In this study, high-resolution and time-series raw data from field soybean populations were collected using UAVs. The RGB (red, green, and blue) and infrared images are used as inputs to construct the multimodal image segmentation model—the RGB & Infrared Feature Fusion Segmentation Network (RIFSeg-Net). Subsequently, the segment anything model was employed to extract complete individual leaves from the segmentation results obtained from RIFSeg-Net. These leaf aspect ratios facilitated the accurate categorization of soybean populations into 2 distinct varieties: oval leaf type variety and lanceolate leaf type variety. Finally, dynamic modeling was conducted to identify 5 phenotypic traits associated with the canopy development rate that differed substantially among the classified soybean varieties. The results showed that the developed multimodal image segmentation model RIFSeg-Net for extracting soybean canopy cover from UAV images outperformed traditional deep learning image segmentation networks (precision = 0.94, recall = 0.93, F1-score = 0.93). The proposed method has high practical value in the field of germplasm resource identification. This approach could lead to the use of a practical tool for further genotypic differentiation analysis and the selection of target genes.

## Introduction

Soybeans have a rich history of cultivation and are considered traditional crops [1]. They serve as both oilseeds and grains and play an important role as industrial raw materials and economic crops. However, as typical land-intensive products, maize and rice yield in many countries and regions is far lower than that of major crops such as maize and rice [2]. To bridge the supply–demand gap, the development of new varieties with yield as the target trait is of paramount importance [3,4]. The yield potential of dicotyledonous broadleaf crops, which are important dicotyledonous broadleaf crops, is largely determined by their canopy structure. Currently, it is unclear whether the establishment speed of the soybean canopy is physiologically linked to yield [5]. Soybean pods are primarily filled after canopy closure. This has led to the prevalent use of early-maturing soybean varieties that provide rapid canopy closure. Therefore, monitoring the early vigor and canopy development of different soybean genotypes is crucial for understanding the relationship between soybean yield and protein content [6]. Both early vigor and canopy development are related to growth patterns, and agronomically meaningful characteristics are urgently needed to link growth-related phenotypes to genotypes [7–10].

Traditional growth monitoring methods require breeders to conduct extensive field surveys, which are time-consuming and labor-intensive [11]. High-throughput phenotyping is a crucial means to address this labor-intensive challenge [12]. Although high-throughput phenotyping is a relatively new approach in agriculture, remote sensing technology based on unmanned aerial vehicle (UAV) platforms is a mature research field [13,14]. The cost-effectiveness of remote sensing technology has promoted various related studies in precision agriculture, enabling breeders to monitor crop characteristics and temporal and spatial variations using UAV platforms [15]. Image processing methods based on UAV platforms have proven effective for monitoring crop canopy cover and early vigor [16,17]. For example, previous studies used drones equipped with various sensors to study parameters such as the leaf area index [18], aboveground biomass [19], maturity [20], wilting stage, and yield prediction [21,22]. UAVs are advantageous for collecting large amounts of raw field data in high-throughput cases, making them valuable tools for data acquisition.

The analysis of these data requires advanced image processing methods [23]. Researchers have attempted to extract crop canopy cover using threshold segmentation methods [24,25]. However, threshold segmentation methods are sensitive to light intensity, and images collected at different times often require manual intervention to achieve good segmentation results. With the widespread application of machine learning, especially deep learning methods, many of the drawbacks of threshold segmentation methods have been effectively overcome [26]. Notably, well-trained deep learning models can automatically remove weeds from images. Nevertheless, constructing datasets for training deep learning models requires substantial resources, and the existing image segmentation networks have reached a bottleneck in segmentation accuracy. Improving the accuracy of deep learning algorithms is a major research direction. Multisource data fusion is a promising solution [27]. By inputting multimodal data into deep learning models, complementary information from different dimensions can further enhance image segmentation accuracy.

However, a good image segmentation method combined with time-series images collected by UAVs can be used to accurately assess canopy development. However, the selection of germplasm resources typically requires comprehensive consideration, and low-level canopy cover features can lead to errors [28]. Dynamic modeling can be used to infer intermediate features from lower-level features [5,20]. Researchers use prior physiological knowledge in the form of parameters or semiparametric growth models to extract intermediate traits, such as traits related to critical growth periods, specific time points, and specific temperatures. Some researchers use nonlinear function dynamic modeling and extract traits that are linked to yield and maturity. This time-series dynamic phenotyping method based on high-throughput phenotyping of phenotypes can help breeders derive a plethora of "hidden" parameters as useful phenotypic traits in breeding environments [9,29].

In this study, we collected 200 typical soybean varieties from the northeast region of China and utilized UAVs to gather multisource phenotypic data. To overcome the challenges encountered in high-throughput phenotyping, we devised a multimodal deep learning model specifically tailored for soybean canopy segmentation in the field. Leveraging infrared information substantially enhances the segmentation accuracy of soybean canopies in RGB images captured by UAVs. The segmentation results were processed using a large-scale artificial intelligence model, facilitating the extraction of individual leaves and the subsequent calculation of leaf aspect ratios. The soybean genotypes collected were then categorized based on these aspect ratios, resulting in the subdivision of 200 soybean varieties into 4 distinct subgroups. Employing dynamic modeling, we extracted and constructed 5 phenotypic parameters (including canopy cover at 500 °C cumulative temperature [CC_500TT_], canopy cover at 1,000 °C cumulative temperature [CC_1000TT_], canopy cover at 1,500 °C cumulative temperature [CC1_500TT_], the cumulative temperature required for 30% canopy cover [TT_30%CC_], and the cumulative temperature required for 50% canopy cover [TT_50%CC_]) related to canopy development dynamics using time-series UAV image data. Finally, we conducted a detailed analysis of the canopy development of soybean plants across different subgroups. This comprehensive approach represents a typical UAV phenotyping process, showcasing the integration of advanced technologies and methodologies to derive meaningful insights into soybean growth patterns and dynamics. High-throughput phenotyping is typically applied to field germplasm resource identification and provides powerful tools for the breeding of high-yield soybean varieties.

## Materials and Methods

### Experimental design

**Fig. 1.**
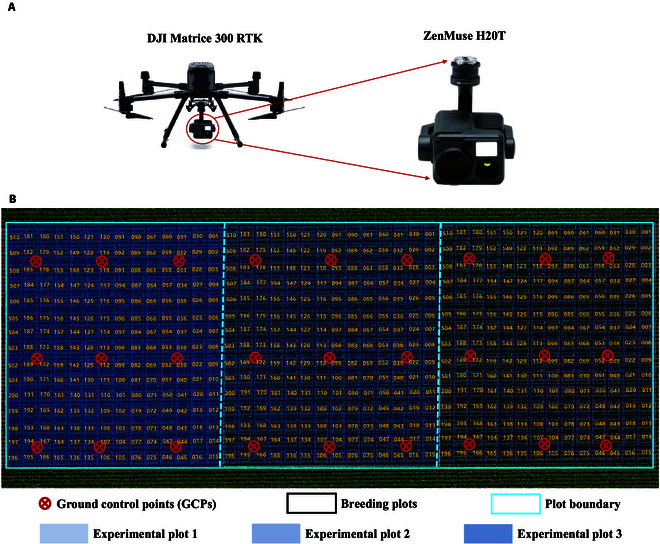
Overview of the performed field experiments: The UAV and sensors used for data collection (A) and the trial with group locations (the yellow boxes) (B).

In the present study, we used a diverse collection of 200 soybean cultivars collected from Northeast China, and these samples spanned 5 temperature accumulation zones. The soybean germplasms were evaluated at the Changchun experimental field located in the northeast region of China in 2023. Changchun (44.06°N, 118.13°E) has a continental climate with 4 distinct seasons and is located in the temperate climate zone. The average rainfall, average humidity, and average temperature in Changchun were 522 to 615 mm, 67.83%, and 14 to 25 °C, respectively. Two hundred soybean germplasms were planted in 3 replicates; each soybean germplasm was planted in 4 rows with a row length of 200 cm, and the spacing between rows was 60 cm. Standard agronomic practices were followed to grow the soybean plants. Phenotypic flowering time data were collected by counting the number of flowering soybean plants in each plot at the flowering stage, and the average flowering time was used for the final analysis. An overview of the field experiments is shown in Fig. 1.

### High-throughput UAV measurements

High-throughput measurements were obtained utilizing an unmanned aerial platform. We employed a fivefold zoom interchangeable lens camera (H20T) featuring a full-frame sensor with dimensions of 5,184 × 3,888 pixels as the primary sensor. The platforms employed included a Warp M300 UAV (Shenzhen DJI Innovation Technology Co., Ltd., Shenzhen, China) and a Ronin-MX gimbal (Shenzhen DJI Innovation Technology Co., Ltd., Shenzhen, China). During each flight, the UAV meticulously followed a predetermined flight path, capturing both visible and infrared data with an 80% horizontal overlap and 80% vertical overlap at consistent intervals. Along the flight path, a speed of 3.0 m/s was maintained, resulting in a ground sampling distance of 0.38 cm/pixel. The data collection cycle was set at 1 to 2 flights per week.

### Overview of the methodology flow

**Fig. 2.**
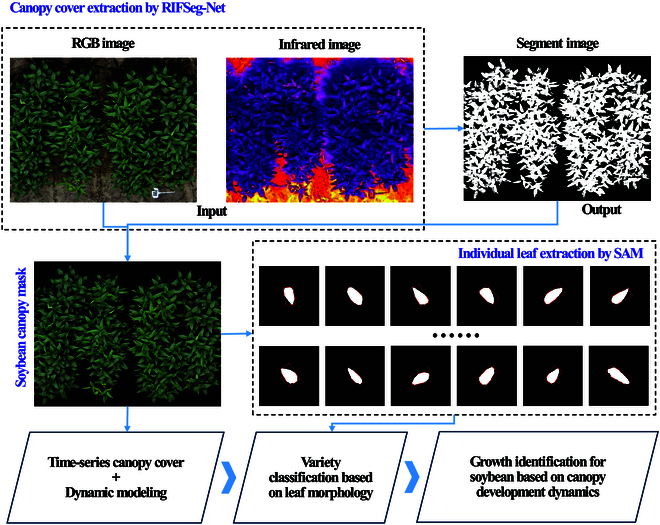
Methodology flowchart. The canopies of soybean plants were segmented and masked from field UAV images using RIFSeg-Net. Individual leaves in the canopy were extracted using the SAM to classify soybeans of different genotypes in terms of the aspect ratio. Finally, the rate of establishment of soybean canopies in different subgroups was evaluated.

The main flow of the proposed methodology is shown in Fig. 2 and mainly consists of 3 parts: (a) A set of multimodal deep learning models is developed. Taking RGB and infrared images as inputs, infrared features are used to improve the accuracy of soybean canopy segmentation from RGB images. (b) The canopy in the RGB image was removed using the segmentation results from the previous step. Individual leaves with intact soybean canopies were extracted using the segment anything model (SAM), and the aspect ratios were calculated for different genotypes. (c) Classification of soybean populations collected for the study based on leaf morphology. The rate of establishment of soybean canopies in different subpopulations was analyzed in conjunction with time-series data.

### Segmentation network

#### Model structure

**Fig. 3.**
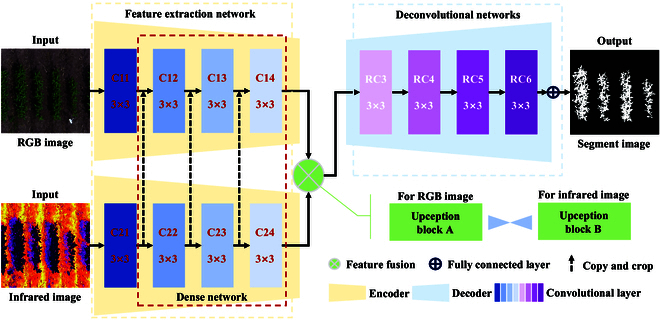
RIFSeg-Net consists of 3 modules: a feature extraction module, a feature fusion module, and a feature resolution module. The feature extraction module consists of 2 encoders, which are used to extract features from RGB and infrared images; the feature fusion module consists of 2 Upception blocks, which are used to ensure that the number of channels for extracting features from RGB and infrared images is the same to facilitate fusion; and the feature resolution module consists of a decoder, which is used to recover the resolution of the feature map. The encoder and decoder regions are symmetrically designed. At the end of RIFSeg-Net, a sigmoid function is used to obtain a probability map of the segmentation result.

We propose a novel deep learning network called the RGB & Infrared Feature Fusion Segmentation Network (RIFSeg-Net) (Fig. 3). Using the encoder–decoder design concept, 2 encoders are constructed for feature extraction using ResNet as the backbone (the backbone is replaceable and contains 5 structures: ResNet-18, ResNet-34, ResNet-50, ResNet-101, and ResNet-152) [30]. A new decoder is developed to obtain the feature map resolution for final application in field soybean image segmentation. The backbone network of RIFSeg-Net is adapted from well-established fusion networks [31]. In the frameworks design, we strategically decreased the number of hidden layers specifically for the binary classification problem, aiming to enhance the overall efficiency. Furthermore, the incorporation of functions such as leaky ReLU and sigmoid in both the encoder and decoder components renders RIFSeg-Net particularly suitable for binary classification tasks. These modifications contribute to the model's effectiveness and efficiency in addressing the specific challenges posed by binary classification problems.

*Encoders*: We designed 2 encoders to extract features from RGB and IR (infrared) images. The structures of the 2 encoders are identical, except for the number of input channels in the first layer. We use ResNet as the feature extractor. To avoid too much loss of spatial information in the feature map, the average pooling layer and the fully connected layer of ResNet are removed. This also helps reduce the model size. ResNet starts with an initial block that consists of a convolution layer, a BN (batch normalization) layer, and a leaky ReLU activation function. Since ResNet is designed for 3-channel RGB images, we modify the number of input channels in the convolution layer in the initial block of the IR encoder to 1. After the initial block, the maximum pooling layer and 4 residual layers are used in turn to gradually reduce the resolution and increase the number of channels in the elemental map.

**Fig. 4.**
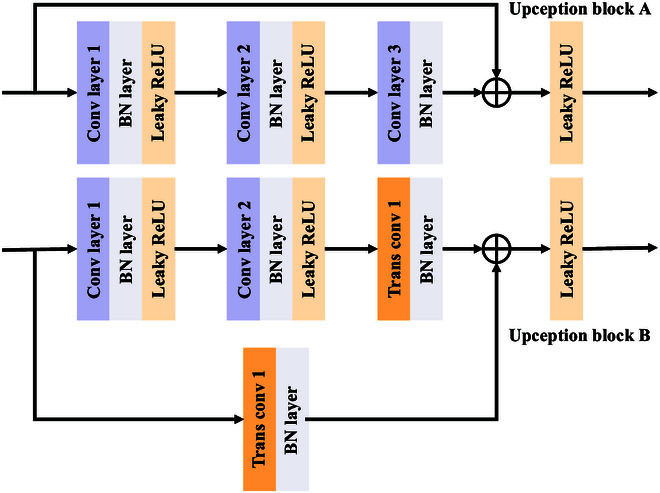
The architecture of the Upception block. In block A, there are 3 convolutional layers that maintain the resolution and number of feature channels. The input and convolutional layers output feature maps through elementwise summation.

*Decoder*: The decoder is mainly used to obtain segmentation results. With the decoder, the resolution of the feature map is gradually restored to the resolution of the input image. In addition, we construct a network module called Upception before decoding. It consists of 2 subblocks: A and B. Block A keeps the resolution and the number of feature mapping channels unchanged. Block B increases the resolution and decreases the number of feature mapping channels. Upception can fuse RGB and IR feature maps through pixel-by-pixel summation, and the shape of the feature maps is not changed after fusion. In block B, the first convolutional layer keeps the resolution constant and reduces the number of feature channels by a factor of 2. The second convolutional layer keeps the resolution and the number of feature channels constant. Transposed convolutional layer 1 keeps the number of channels constant and increases the resolution by a factor of 2. Therefore, transposed convolutional layer 2 is needed to increase the input resolution and reduce the number of feature channels before summation. The detailed framework of the 2 sets of Upception blocks is shown in Fig. 4. The specific feature extraction details are given in Table 1.

**Table 1.** The specific structure of the 2 Upception blocks. A total of 5 decoding layers are included, and C, H, and W represent the number of channels, height, and width of the feature map, respectively.

	Name	Stride	Padding	Input size	Output size	Kernel size
Upception block A	Conv 1	1	0	c×h×w	c×h×w	1×1
Conv 2	1	1	c×h×w	c×h×w	3×3
Conv 3	1	1	c×h×w	c×h×w	3×3
Upception block B	Conv 1	1	0	c×h×w	c/2×h×w	1×1
Conv 2	1	1	c/2×h×w	c/2×h×w	3×3
TransConv 1	2	1	c/2×h×w	c/2×2 h×2w	2×2
TransConv 2	3	0	c×h×w	c/2×2 h×2w	2×2

#### Main functions

The loss function of RIFSeg-Net can be divided into 2 main parts: *L_SSIM_* and *L_TV_* [31]. Among them, *L_SSIM_* is used to measure the structural similarity of an image by combining the 3 aspects of brightness, contrast, and structure to measure the quality of the input image. RIFSeg-Net involves 2 forms of images as inputs, and the LSSIM function is needed to measure which of the synthesized images are most similar to the RGB and IR images. For example, the *SSIM* (*I_A_*, *I_F_* |*W*) and *SSIM* (*I_B_*, *I_F_* |*W*) indices are computed separately using the *L_SSIM_* function, where *W* denotes a sliding window of size m*n, *SSIM* (*I_A_*, *I_F_* |*W*) denotes the similarity between the RGB image and the fused image, and *SSIM* (*I_B_*, *I_F_* |*W*) denotes the similarity between the IR image and the fused image. If *SSIM* (*I_A_*, *I_F_* |*W*) is greater than *SSIM* (*I_B_*, *I_F_* |*W*), *I_A_* and *I_F_* are more similar in a sliding window of size *W*, and the fused image will retain more RGB information in the window of *W*. The main formulas involved in the loss function of the *L_SSIM_* part are as follows:

$$E\left(I|W\right)=\frac{1}{m}\sum_{i=1}^{m\times n}P_{i}$$ (1)

$$L_{\text{SSIM}}=1-\frac{1}{N}\sum_{W=1}^{N}\text{Score}\left(I_{A},I_{B},I_{F}|W\right)$$ (2)

In this case, the first formula is used to calculate the average value within the sliding window, and the result with the highest similarity to the fused image is output to calculate the corresponding score; the second formula is the overall calculation of the *L_SSIM_* function, where *N* denotes the total number of sliding windows, and the average value is obtained and then subtracted from 1 to be used as the loss function.

For the second loss function, the total variation (*L_TV_*) is a measure of image noise. The *L_TV_* utilizes the square of the difference between the horizontal and vertical pixels and then sums each pixel to calculate the total variation. If there is noise, the variation between pixels will be large, and the total variation may be very large. The specific formula is as follows:

$$R\left(i,j\right)=I_{A}\left(i,j\right)-I_{F}\left(i,j\right)$$ (3)

$$L_{\mathrm{TV}}=\sum_{i,j}\left({\left\|R\left(i,j+1\right)-R\left(i,j\right)\right\|}_{2}+{\left\|R\left(i,+1j\right)-R\left(i,j\right)\right\|}_{2}\right)$$ (4)

In this case, the corresponding pixels in *I_A_* and *I_F_* are first subtracted to obtain *R* (*i*, *j*), and the total variation is subsequently obtained. The *L_SIMM_* and *L_TV_* are combined as the loss function of RIFSeg-Net. However, since the *L_SIMM_* and *L_TV_* are not of uniform order of magnitude, it is easy to lead to an overall weight shift in the network. It is necessary to introduce a balancing parameter *φ* for *L_SIMM_* so that the 2 loss functions are at the same level. Therefore, the overall loss function of RIFSeg-Net is as follows:

$$\begin{array}{c} \text{LOSS}=\varphi L_{\text{SSIM}}+L_{\mathrm{TV}}=\varphi \left(1-\frac{1}{N}\sum_{W=1}^{N}\text{Score}\left(I_{A},I_{B},I_{F}|W\right)\right)+\\ \sum_{i,j}\left({\left\|R\left(i,j+1\right)-R\left(i,j\right)\right\|}_{2}+{\left\|R\left(i,+1j\right)-R\left(i,j\right)\right\|}_{2}\right) \end{array}$$ (5)

### Individual leaf segmentation and phenotype extraction

Before proceeding with further phenotyping, we sought to precisely classify the collected soybean genotypes based on leaf morphology. The SAM was used to extract individual, intact leaves from the canopy segmentation results. Initially, we utilized the segmentation outcomes of RIFSeg-Net to mask the canopies of soybean plants in the original RGB images. Subsequently, the pretrained weights of the SAM were invoked to extract each individual leaf, ensuring that all canopy leaves were successfully isolated. For each genotype, 5 to 10 complete leaves were selected for subsequent analysis. The aspect ratio of the smallest outer rectangle enclosing each leaf was calculated using Python. Subsequently, the aspect ratio served as a foundational criterion for clustering. Initially, the collected soybean genotypes were broadly clustered into 2 categories: the oval leaf type (OLT) variety and the lanceolate leaf type (LLT) variety, recognized as the 2 predominant soybean types. The OLT variety has leaves that closely resemble an oval shape, whereas the LLT variety is characterized by more elongated leaves.

Following this, efforts were made to further refine the classification of the OLT and LLT subgroups. The soybeans belonging to distinct subgroups were then phenotyped in conjunction with the results of phenotype extraction. This meticulous classification based on leaf morphology provides a more nuanced understanding of soybean genotypic variations, facilitating detailed phenotypic analysis and contributing to a comprehensive assessment of soybean diversity.

### Dynamic modeling

Canopy cover data collected at 14 different time points were utilized to create growth curves for 200 distinct soybean varieties [5]. These growth curves were generated by employing Python's LinearGAM library. The GAM, which is a generalized additive model, is a smoothed semiparametric model. It provides a linking function for the relationship between predictor variables and the expected values of dependent variables. This feature enables the automatic modeling of nonlinear relationships, eliminating the need for manual experimentation with various transformations for each variable. The modeled weights can subsequently be employed to predict canopy cover at any given time point or to estimate the effective accumulation temperature.

### Assessment indicators

RIFSeg-Net was used to segment soybean canopy images in the field, which is considered a binary classification problem. In the process of segmentation accuracy assessment, a pixel-level comparison was made between the predicted output and the classification results based on ground truth data. Usually, pixels belonging to the soybean canopy that are correctly predicted are defined as true positives (TP); pixels belonging to the soybean canopy that are incorrectly predicted are defined as false positives (FP); pixels belonging to the background that are correctly predicted are defined as true negatives (TN); and pixels belonging to the background that are incorrectly predicted are defined as false negatives (FN). Based on these rules, the following 3 evaluation metrics were used in this study [32,33].

*Precision*. The proportion of true-positive samples among those predicted to be positive is defined as follows:

$$P=\frac{\mathrm{TP}}{\mathrm{TP}+\mathrm{FP}}$$ (6)

*Recall*. This index reflects how many positive samples in the total sample are correctly predicted and is defined as follows:

$$R=\frac{\mathrm{TP}}{\mathrm{TP}+\mathrm{FN}}$$ (7)

*F1-score*. After the accuracy and recall are calculated, the F1-score can be calculated, which represents the weighted harmonic average of the accuracy and recall. It is used for standardized measurement and is defined as follows:

$$F1-\text{score}=\frac{2\mathrm{PR}}{P+R}$$ (8)

### Network training

First, we preprocessed the UAV images to construct the dataset. For the images of each soybean variety, we divided the region of interest for the time-series data using the data from the last monitoring time point as a benchmark. Slide cropping was performed in strict accordance with uniform dimensions to ensure that time-series images of the same soybean variety were of the same size. All the images were color corrected using a colorimetric card. After preprocessing, we obtained 2,000 field soybean images for growth analysis. A total of 1,200 representative images were selected and manually labeled using Labelme Software, and a dataset for RIFSeg-Net training was constructed. Of these, 1,000 images were used for training and validation of the model (80% of the data were used for training, and 20% were used for validation). The remaining 200 images were used for testing the model. We used the test set to compare the performances of the RIFSeg-Net models with different backbones. In addition, we selected an FCN (Fully Convolutional Network) [34], UNet [35], SegNet [36], FuseNet [37], MFNet [38], and PSPNet [39] for comparison; these models were trained with the same training strategy and subsequently compared with RIFSeg-Net. Finally, RIFSeg-Net was trained many times. During the training process, each epoch included 500 batches with a size of 1. Training losses declined quickly over the first 100 batches and then slowed. The model was trained on a workstation with a 2 Intel Xeon (R) Gold 6148 CPU (central processing unit), 256 GB RAM (random access memory), and an NVIDIA Quadro RTX6000 GPU (graphic processing unit).

## Results

### Modeling validation studies

To assess the effectiveness of RIFSeg-Net, we conducted rigorous testing on an independent dataset in 2 distinct phases: (a) Comparative Model Accuracy Evaluation: In this phase, we systematically compared the accuracy of RIFSeg-Net against that of several established models. The comparisons included an FCN, UNet, SegNet, FuseNet, MFNet, PSPNet, and RTFNet. These models represent a comprehensive spectrum of image segmentation methods, including those designed for multimodal data fusion. (b) Backbone Architecture Analysis: To gauge the impact of different backbone architectures on the performance of RIFSeg-Net, we employed a variety of ResNet models, specifically ResNet-18, ResNet-34, ResNet-50, ResNet-101, and ResNet-152, to construct diverse versions of RIFSeg-Net. We subsequently assessed the model's effectiveness with each of these backbone configurations. For specific details, please refer to Table 2 for a comprehensive breakdown.

**Table 2.** Performance of different models for independent test sets. Nos. 1 to 6 represent the 6 groups of established models. No. 7 to No. 11 represent 5 RIFSeg-Nets constructed separately using different backbones.

No.	Methods	*P*	*R*	F1-score
1	FCN	0.85	0.77	0.81
2	U-net	0.88	0.82	0.85
3	SegNet	0.88	0.85	0.86
4	FuseNet	0.86	0.81	0.83
5	MFNet	0.91	0.87	0.89
6	PSPNet	0.91	0.91	0.91
7	RTFNet	0.92	0.90	0.91
8	RIFSeg-Net-18	0.90	0.86	0.88
9	RIFSeg-Net-34	0.89	0.88	0.88
10	RIFSeg-Net-50	0.94	0.93	0.93
11	RIFSeg-Net-101	0.92	0.91	0.91
12	RIFSeg-Net-152	0.93	0.91	0.92

As depicted in Table 2, it becomes evident that the multimodal model exhibits a clear advantage over conventional deep learning image segmentation models in terms of accuracy. The incorporation of multidimensional information as input proves to be instrumental in enhancing the model’s precision. When comparing various backbone architectures, it is evident that the most favorable performance is achieved with ResNet-50. The RIFSeg-Net configuration utilizing ResNet-50 achieved the highest F1-score, reaching an impressive 0.93. Notably, deeper feature extraction, while potentially beneficial in certain contexts, can introduce information redundancy, which may, in turn, have an adverse effect on model accuracy.

### Variety classification based on leaf morphology

**Fig. 5.**
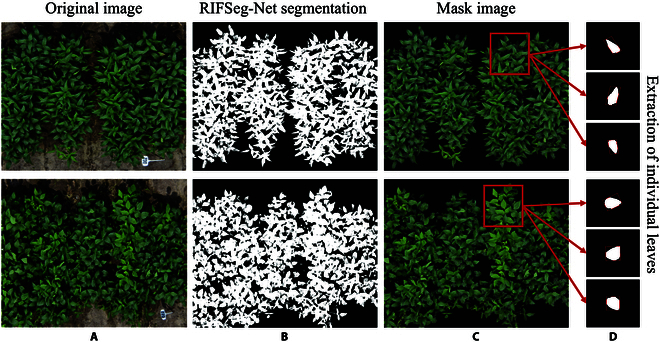
Visualization of image processing for typical OLT and LLT soybean varieties. The original image (A), the RIFSeg-Net segmentation result (B), the image masked with the RIFSeg-Net segmentation result (C), and the image processing result of an individual leaf segmented using the SAM (D).

The original image was masked using the RIF model segmentation results as a baseline. Furthermore, masked images were used for the extraction of individual, intact leaves from the soybean canopy. The extraction of individual leaves was performed using the large model SAM [40]. This is a very GPU-consuming and time-consuming task. The fine-tuned SAM effectively extracted the individual leaves from the soybean canopy mask image. We then used a Python image processing algorithm to extract the minimum outer rectangles of the individual leaves. A visualization of the image processing series is shown in Fig. 5. In this session, all the leaves from the canopy were extracted. The extraction results for only some of the canopy leaves are shown in Fig. 5. All leaf analysis results were collated and uploaded as separate files, the details of which can be downloaded to view File S1. The length and width of the minimum outer rectangle were used to calculate the aspect ratio of the leaves, which was subsequently used to classify the 200 germplasms sampled. In the classification process, 2 broad categories were first classified: the OLT variety and the LLT variety. These 2 soybean leaf types are widely recognized. Furthermore, we subdivided the 2 subclasses based on the OLT and LLT. Ultimately, the collection of 200 soybean varieties was classified into 4 categories. The number of soybean varieties in each category is shown in Table 3.

**Table 3.** Statistics for the number of soybean varieties classified into different categories

	OLT		LLT	
Number	58		142	
	Subclass A	Subclass B	Subclass A	Subclass B
Number	35	23	57	85

### Dynamic modeling

**Fig. 6.**
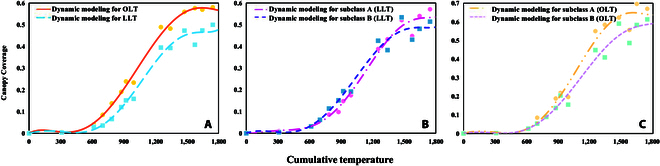
Dynamic modeling results of canopy development for different varieties of soybean. (A) Comparison between the OLT and LLT groups. (B) Comparison of the 2 subclasses of LLTs. (C) Comparison of the 2 subclasses of OLTs.

Based on the leaf morphology classification results, we conducted an analysis of soybean canopy development for the different varieties via dynamic modeling, as illustrated in Fig. 6. Figure 6A shows a comparison between soybean plants with OLT and LLT leaf shapes. It is evident that, compared with those with LLT leaf shapes, soybean plants with OLT leaf shapes exhibit faster canopy development and achieve greater final canopy cover. In Fig. 6B, we compare 2 subclasses within the LLT foliation, where the LLT_A_ subclass demonstrates slower canopy development but achieves a greater final canopy cover in contrast to the LLT_B_ subclass. Figure 6C displays a comparison of the results between 2 subclasses within the OLT leaf shape category, with the OLT_A_ subclass showing accelerated canopy development and greater final canopy coverage than the OLT_B_ subclass. The distinct variations in dynamic modeling outcomes among the 200 soybean germplasm resources became evident after categorization into these 4 groups.

### Canopy development in different populations

**Fig. 7.**
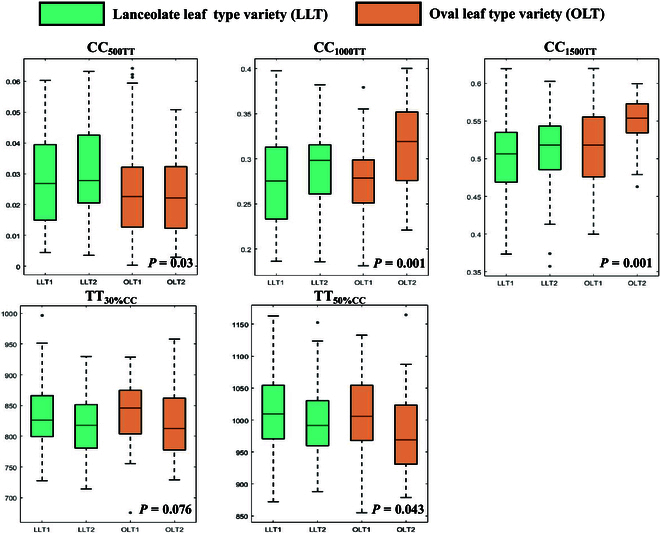
Comparison of the phenotypic trait variations associated with soybean canopy cover among the different groups. For each box plot, different boxes represent different subclasses.

We categorized the 200 soybean germplasms into 4 groups and extracted 5 phenotypic parameters from the dynamic modeling results for the different genotypes. These parameters included CC_500TT_, CC_1000TT_, CC1_500TT_, TT_30%CC_, and TT_50%CC_. We utilized MATLAB to create box plots to compare the variations in phenotypic traits associated with soybean canopy cover among these 4 groups, as shown in Fig. 7. The results clearly revealed substantial differences among the groups.

## Discussion

### General assessment of the proposed methodology

We harnessed the power of UAV multisource data fusion in tandem with cutting-edge deep learning algorithms and dynamic models to comprehensively characterize a collection of 200 soybean cultivars. This methodological approach is at the forefront of plant phenotyping research and encompasses fields such as image processing, dynamic system modeling, and multisource data fusion. Our UAV, equipped with an array of multisource sensors, enabled us to capture high-temporal-precision soybean canopy image data throughout the entire reproductive cycle. In contrast to the manual phenotyping procedures traditionally employed in agricultural research, the utilization of UAV technology empowers us to conduct high-throughput, large-scale field experiments. The techniques elucidated in this paper are well suited for the efficient phenotyping of crop populations harboring a multitude of genotypic variations under field conditions. They provide breeders with invaluable trait-related information, enabling the detection and quantification of disparities among groups of genotypes [41,42].

In this study, we utilized a UAV to capture soybean canopy image data across 14 flights conducted at regular intervals throughout the reproductive cycle. Unlike frameworks employed in some prior studies, our high-throughput phenotyping platform stands out for its ability to acquire data at a frequent temporal scale, offering a distinct advantage over traditional UAV methods. However, as we delved into the data processing and analysis phases, we recognized the importance of adopting specific data acquisition strategies. For instance, focusing on intensive data collection during pivotal fertility periods proved to be particularly effective. This approach allowed us to closely monitor soybean growth dynamics, providing a detailed understanding of the intricate processes involved. Implementing targeted data collection programs aligned with the critical phases of crop growth and development holds the promise of yielding even more valuable insights. This strategic approach enhances the precision of our observations, ensuring that the data acquired during key growth stages contributes substantially to our overall comprehension of soybean behavior and performance.

### The importance of multimodal data fusion

The deep learning model, fueled by the fusion of multimodal data inputs, enables us to automatically capture dynamic canopy cover and monitor soybean growth in the field with high precision. In contrast to the threshold segmentation methods commonly used in previous research, the deep learning model is not constrained by variations in light intensity during data collection and is capable of minimizing the impact of weeds on canopy cover extraction to the greatest extent possible. Furthermore, by integrating information from multiple data sources, the model can reduce errors associated with single-modal approaches, thereby enhancing the accuracy and performance of respective modeling tasks, ultimately achieving more precise results. However, importantly, multimodal data fusion models typically require multidimensional inputs, which implies a trade-off with inference speed during the data processing phase [43,44]. For agricultural applications, although the efficiency and accuracy of phenotype extraction are of paramount importance, real-time image processing tasks are not extensively involved. Therefore, the inference speed of RIFSeg-Net is acceptable. Relevant studies have confirmed that multimodal data fusion is a powerful approach for tackling complex problems and leveraging diverse data, thus contributing to advancements in phenotype analysis research and addressing challenges in practical field applications [27,45].

### Implications of dynamic modeling

Utilizing the “S” (sigmoid) growth function to fit time-series parameters allows us to establish biologically meaningful and reliable parameters for characterizing genotype differences during growth and development processes [5]. Dynamic system models can mitigate the phenotypic errors that might occur at specific time points. However, the choice of the fitting function is crucial, as incorrect selection may result in substantial errors in phenotype analysis. Therefore, a solid understanding of plant physiological mechanisms and knowledge about the growth and development of plants are indispensable [46]. In the utilization of dynamic system models, precise phenotypic parameters during critical growth periods are paramount. For instance, phenotype parameters at the initial time point and canopy closure time point are directly related to the overall growth pattern of the corresponding genotypes. It is recommended that researchers and breeders engage in thorough discussions regarding the critical growth periods of the studied soybean variety. This approach is pivotal for describing the general growth patterns of different plant genotypes using dynamic system models. Furthermore, dynamic system models can be employed to analyze early vigor during crop growth. Previous research has indicated a positive correlation between yield and early vigor, but it is also correlated with the timing of mid-season vigor. The importance of early vigor for yield is likely related to the establishment of the canopy, flowering, and pod formation, thus involving source–sink dynamics. Further studies can employ the methods proposed in this study to conduct more detailed phenotype analyses specifically focused on early vigor [47,48].

### Application of high-throughput phenotypes

The proposed multimodal data fusion deep learning model enables high-precision segmentation of drone images. Furthermore, in conjunction with the SAM, the SAM was used for the identification of soybean germplasms and for the analysis of growth-related phenotypic traits. This constitutes a high-throughput, nondestructive approach for precise phenotype identification. The pipeline presented can be directly applied in breeding environments, as it rapidly identifies growth-related phenotypic data for each genotype.

In practical field applications, breeders generally prioritize target traits such as yield and quality. However, these target traits are often decomposable [49]. For instance, previous research has indicated that soybean plant height is negatively correlated with lodging resistance and positively correlated with the number of nodes, and the number of nodes is positively correlated with pod quantity. Therefore, breeders may consider finding trade-offs while focusing on target traits. For example, utilizing high-temporal and high-throughput data collection methods to analyze the dynamics of correlated traits can be beneficial [50]. Time-series data are crucial for accurate parameter estimation. In conclusion, through high-throughput phenotyping, it is possible to quickly identify crop germplasm resources with favorable traits, such as high yield, resistance, and quality [51,52]. This approach can aid breeders in developing new varieties that are more productive and resilient, consequently enhancing both crop quality and yield.

## Data Availability

The data presented in this study are available upon request from the corresponding author.
