# Supplementary figures and images for: Time-Series Field Phenotyping of Soybean Growth Analysis by Combining Multimodal Deep Learning and Dynamic Modeling (part 2 of 2)
Source: Plant Phenomics. 2024 Mar 20;6:0158. doi: 10.34133/plantphenomics.0158 (PMC10959008; doi:10.34133/plantphenomics.0158)

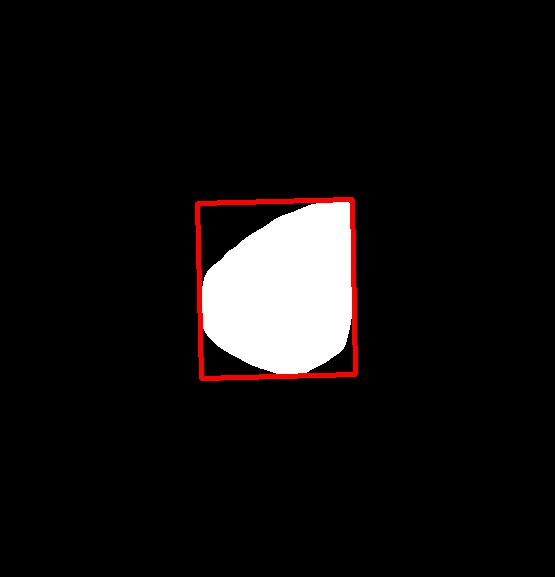

Supplement: Supplementary 1 — File S1 [file plantphenomics.0158.f1.zip › supplementary/0721_021_112.jpg]

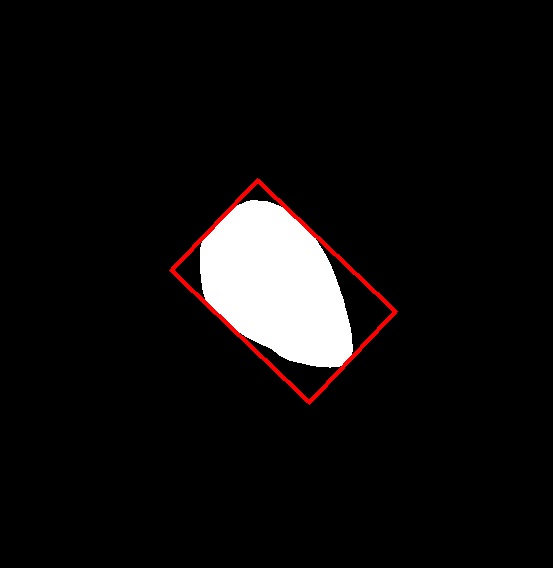

Supplement: Supplementary 1 — File S1 [file plantphenomics.0158.f1.zip › supplementary/0721_021_46.jpg]

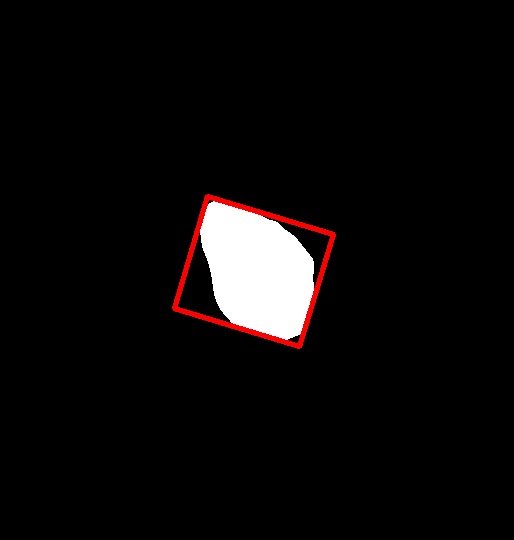

Supplement: Supplementary 1 — File S1 [file plantphenomics.0158.f1.zip › supplementary/0721_021_62.jpg]

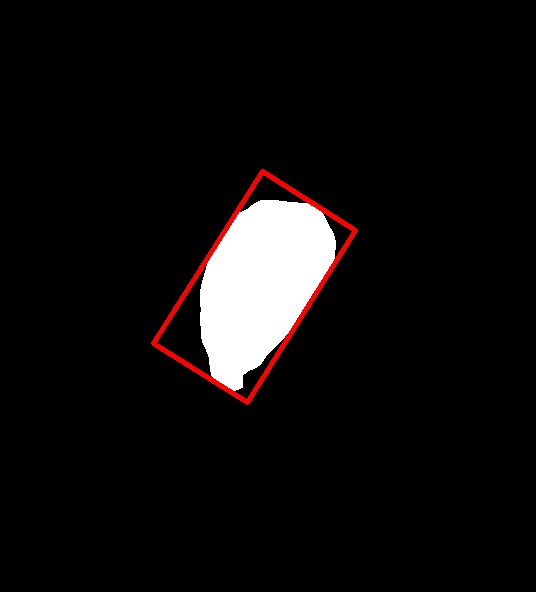

Supplement: Supplementary 1 — File S1 [file plantphenomics.0158.f1.zip › supplementary/0721_021_71.jpg]

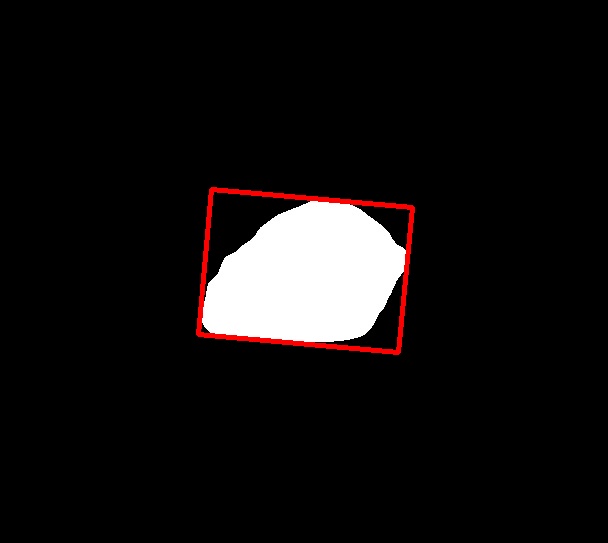

Supplement: Supplementary 1 — File S1 [file plantphenomics.0158.f1.zip › supplementary/0721_021_87.jpg]

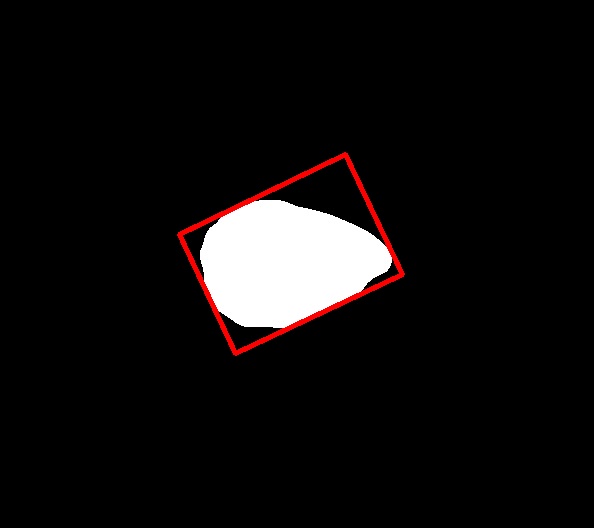

Supplement: Supplementary 1 — File S1 [file plantphenomics.0158.f1.zip › supplementary/0721_022_101.jpg]

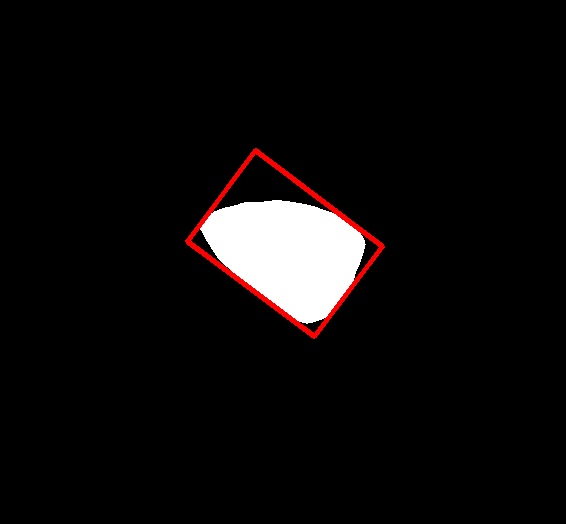

Supplement: Supplementary 1 — File S1 [file plantphenomics.0158.f1.zip › supplementary/0721_022_116.jpg]

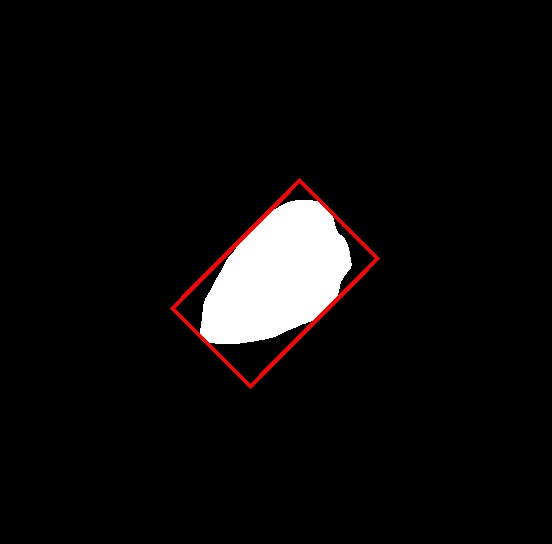

Supplement: Supplementary 1 — File S1 [file plantphenomics.0158.f1.zip › supplementary/0721_022_18.jpg]

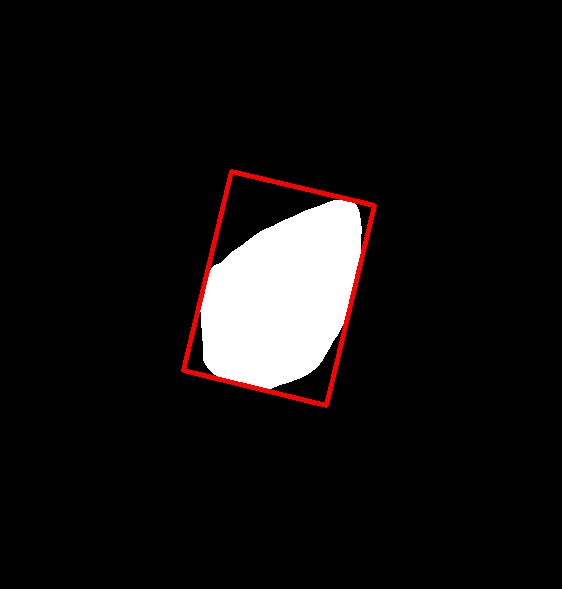

Supplement: Supplementary 1 — File S1 [file plantphenomics.0158.f1.zip › supplementary/0721_022_192.jpg]

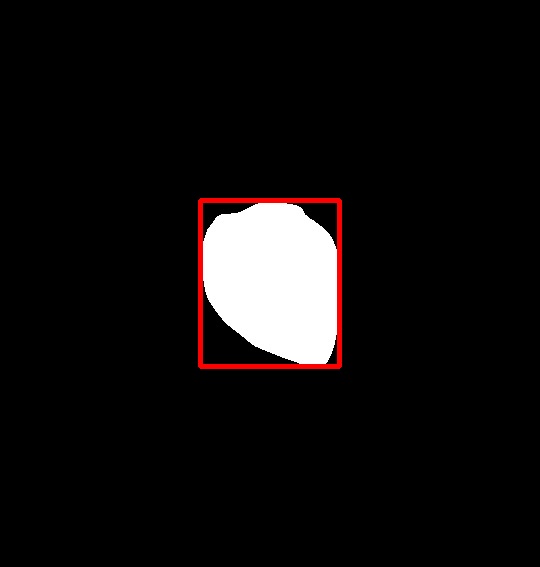

Supplement: Supplementary 1 — File S1 [file plantphenomics.0158.f1.zip › supplementary/0721_022_96.jpg]

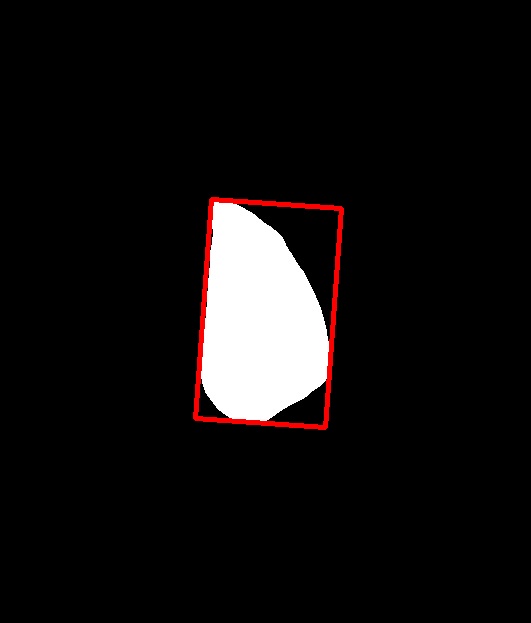

Supplement: Supplementary 1 — File S1 [file plantphenomics.0158.f1.zip › supplementary/0721_023_1.jpg]

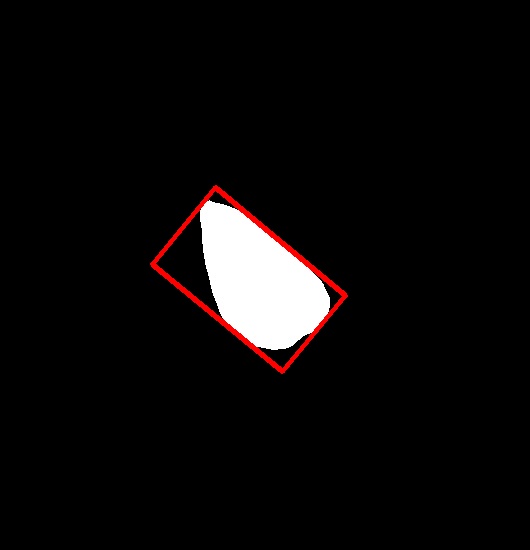

Supplement: Supplementary 1 — File S1 [file plantphenomics.0158.f1.zip › supplementary/0721_023_101.jpg]

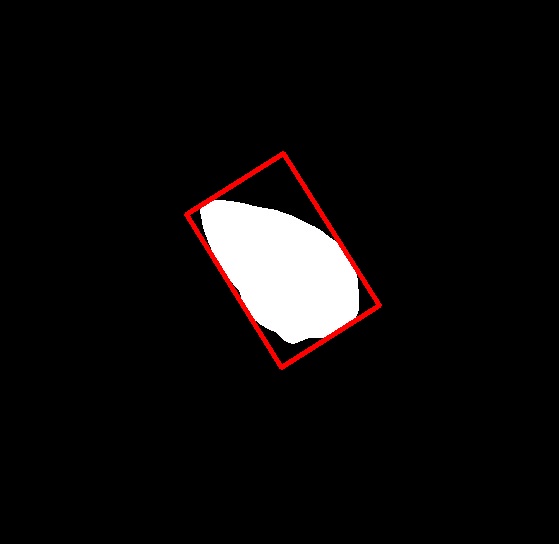

Supplement: Supplementary 1 — File S1 [file plantphenomics.0158.f1.zip › supplementary/0721_023_137.jpg]

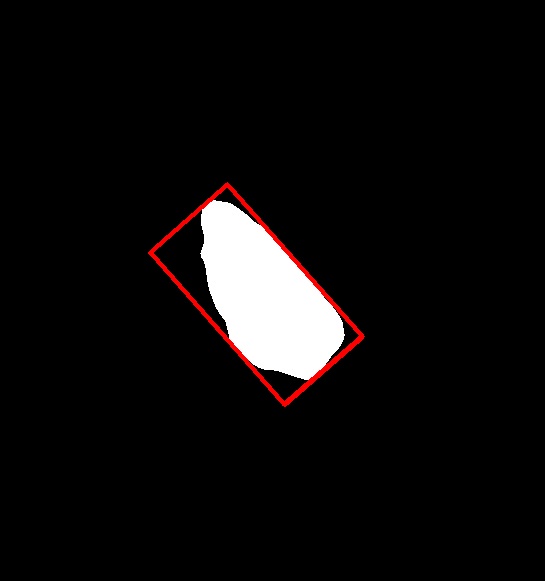

Supplement: Supplementary 1 — File S1 [file plantphenomics.0158.f1.zip › supplementary/0721_023_28.jpg]

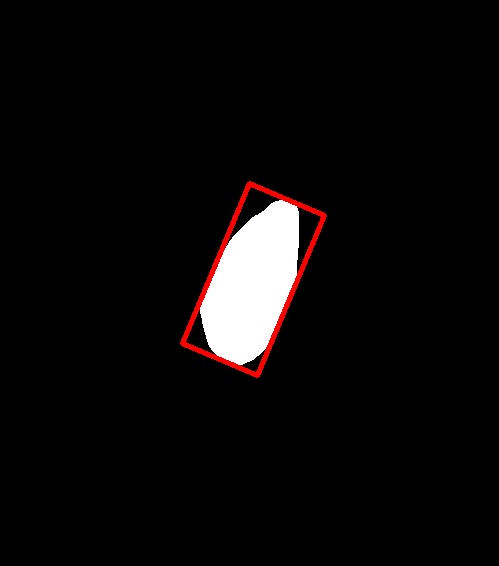

Supplement: Supplementary 1 — File S1 [file plantphenomics.0158.f1.zip › supplementary/0721_023_79.jpg]

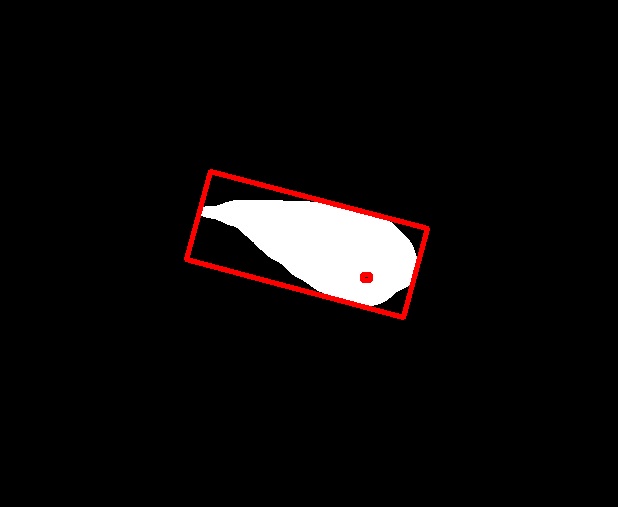

Supplement: Supplementary 1 — File S1 [file plantphenomics.0158.f1.zip › supplementary/0721_024_116.jpg]

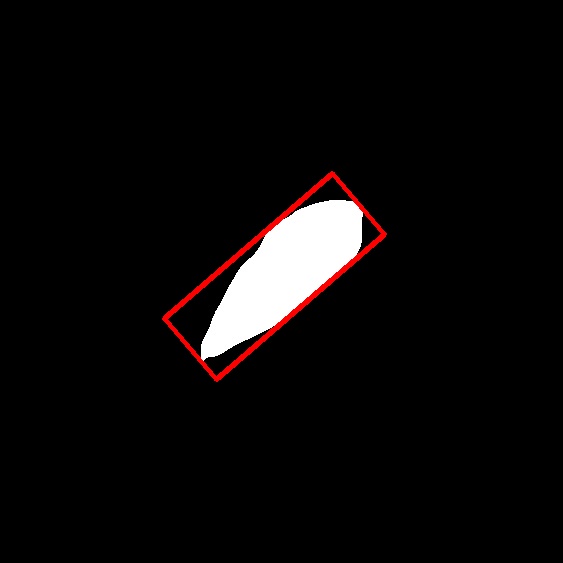

Supplement: Supplementary 1 — File S1 [file plantphenomics.0158.f1.zip › supplementary/0721_024_147.jpg]

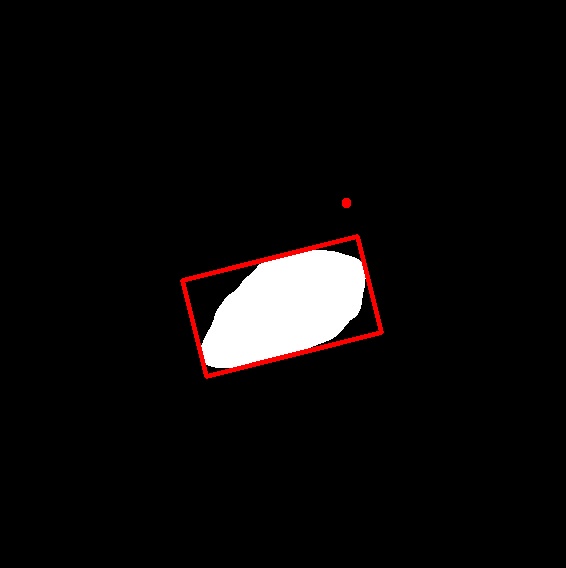

Supplement: Supplementary 1 — File S1 [file plantphenomics.0158.f1.zip › supplementary/0721_024_42.jpg]

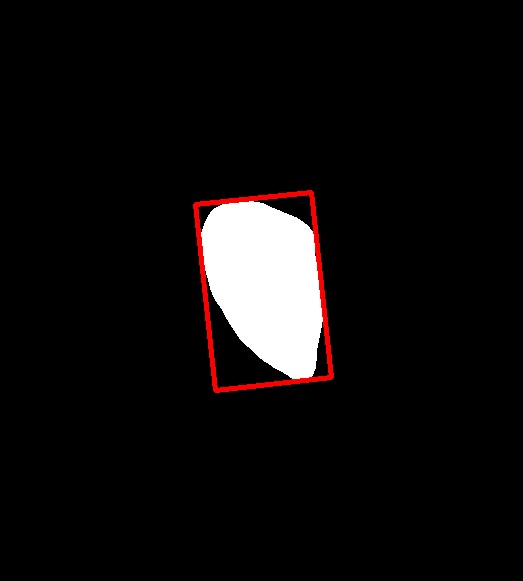

Supplement: Supplementary 1 — File S1 [file plantphenomics.0158.f1.zip › supplementary/0721_024_57.jpg]

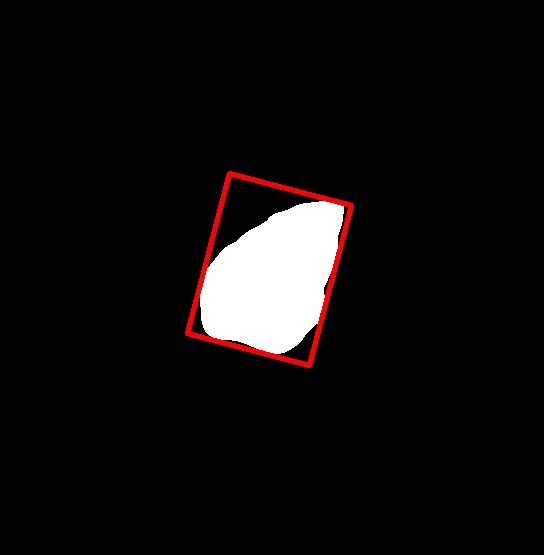

Supplement: Supplementary 1 — File S1 [file plantphenomics.0158.f1.zip › supplementary/0721_024_59.jpg]

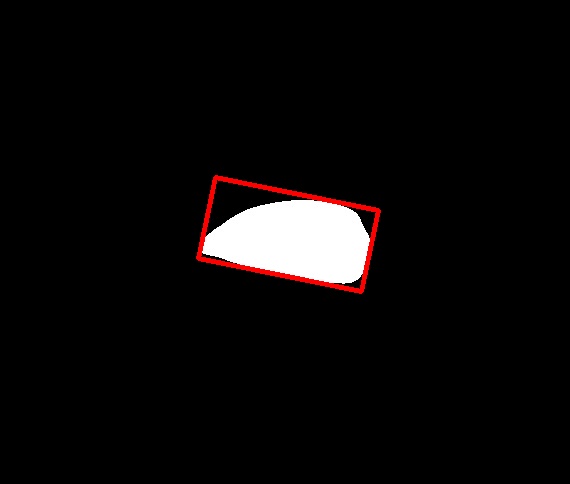

Supplement: Supplementary 1 — File S1 [file plantphenomics.0158.f1.zip › supplementary/0721_025_163.jpg]

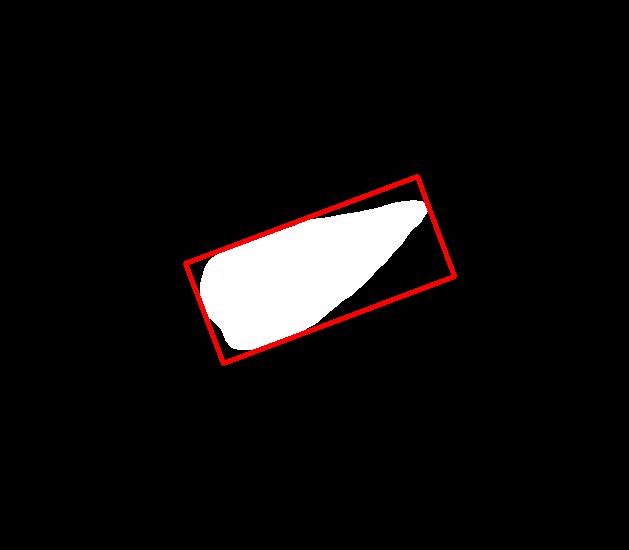

Supplement: Supplementary 1 — File S1 [file plantphenomics.0158.f1.zip › supplementary/0721_025_176.jpg]

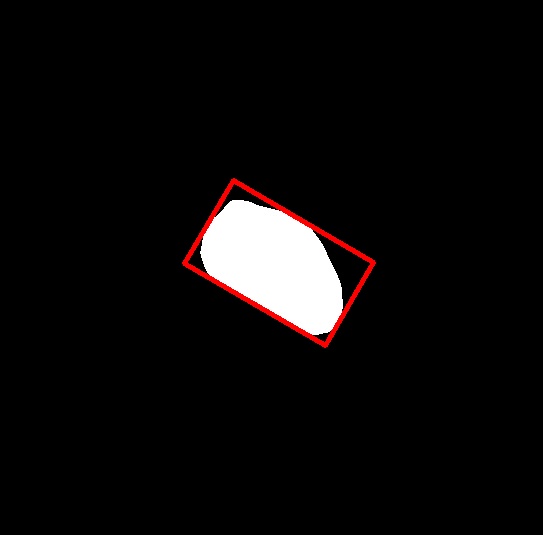

Supplement: Supplementary 1 — File S1 [file plantphenomics.0158.f1.zip › supplementary/0721_025_22.jpg]

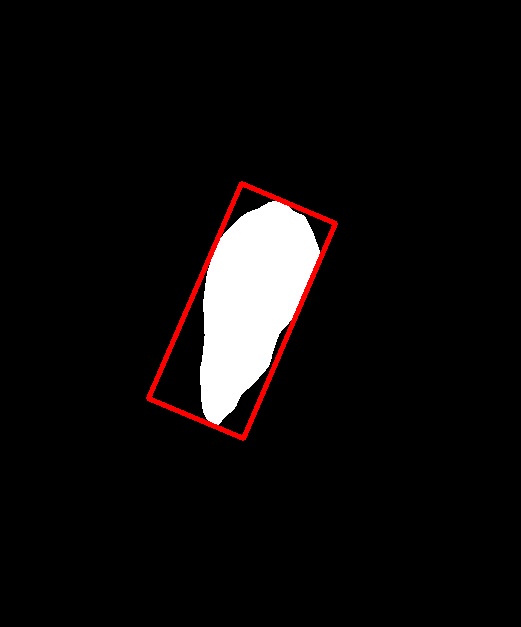

Supplement: Supplementary 1 — File S1 [file plantphenomics.0158.f1.zip › supplementary/0721_025_45.jpg]

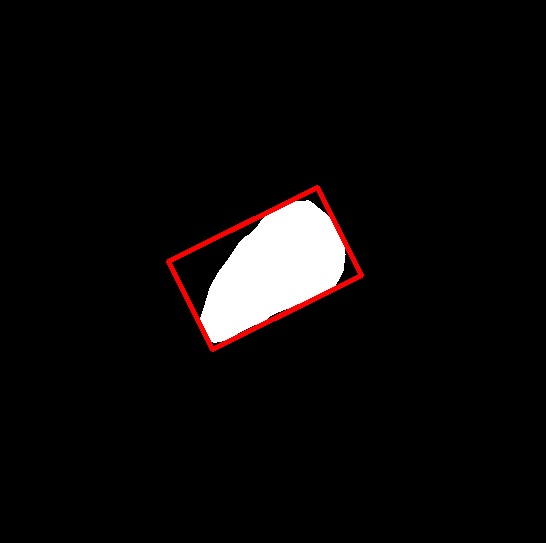

Supplement: Supplementary 1 — File S1 [file plantphenomics.0158.f1.zip › supplementary/0721_025_96.jpg]

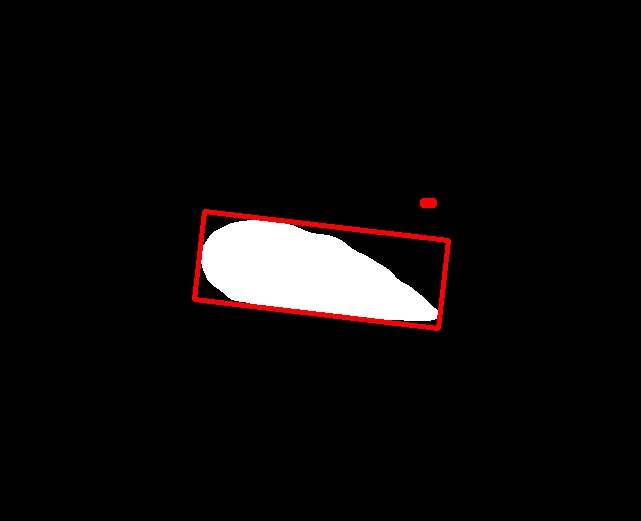

Supplement: Supplementary 1 — File S1 [file plantphenomics.0158.f1.zip › supplementary/0721_026_112.jpg]

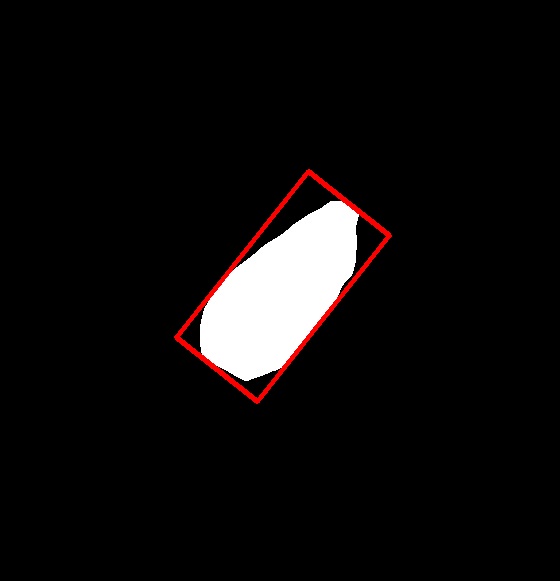

Supplement: Supplementary 1 — File S1 [file plantphenomics.0158.f1.zip › supplementary/0721_026_155.jpg]

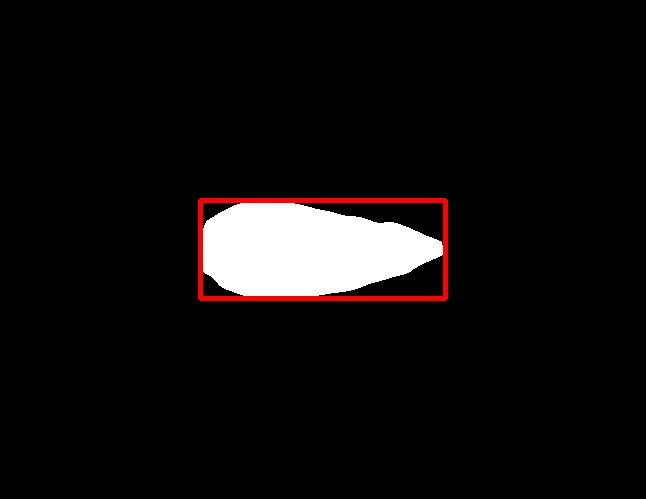

Supplement: Supplementary 1 — File S1 [file plantphenomics.0158.f1.zip › supplementary/0721_026_170.jpg]

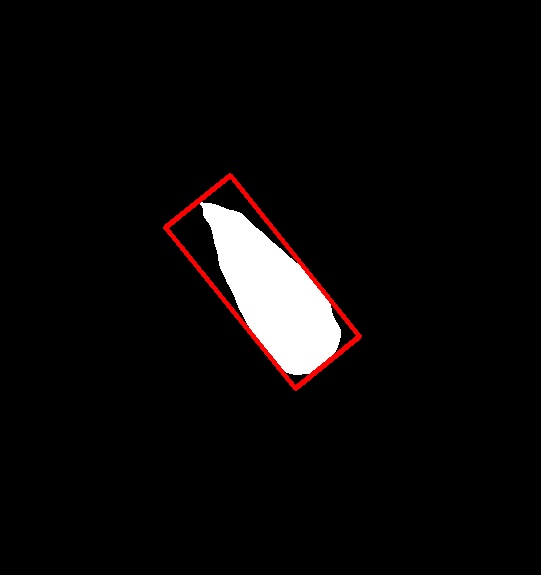

Supplement: Supplementary 1 — File S1 [file plantphenomics.0158.f1.zip › supplementary/0721_026_66.jpg]

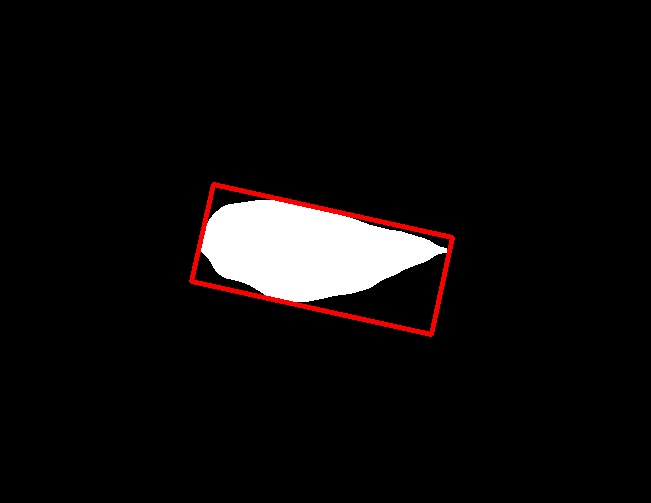

Supplement: Supplementary 1 — File S1 [file plantphenomics.0158.f1.zip › supplementary/0721_026_79.jpg]

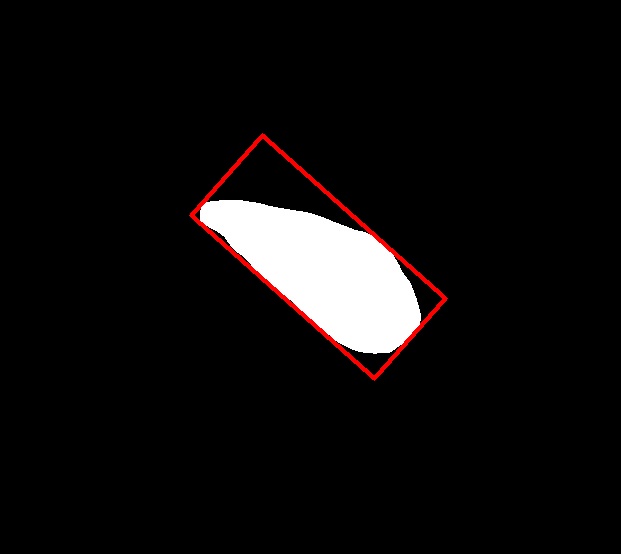

Supplement: Supplementary 1 — File S1 [file plantphenomics.0158.f1.zip › supplementary/0721_027_171.jpg]

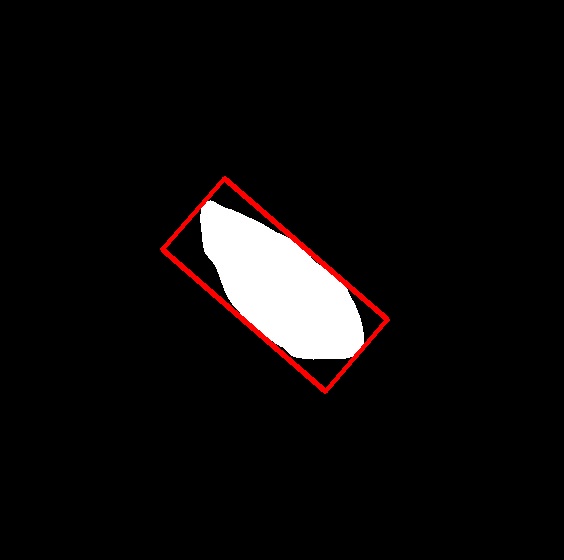

Supplement: Supplementary 1 — File S1 [file plantphenomics.0158.f1.zip › supplementary/0721_027_184.jpg]

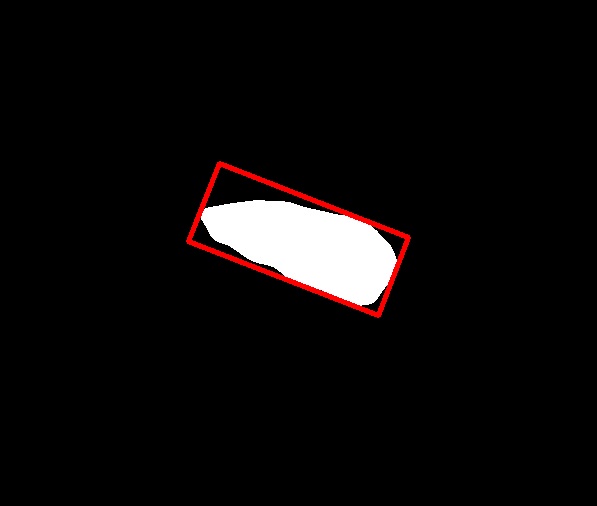

Supplement: Supplementary 1 — File S1 [file plantphenomics.0158.f1.zip › supplementary/0721_027_190.jpg]

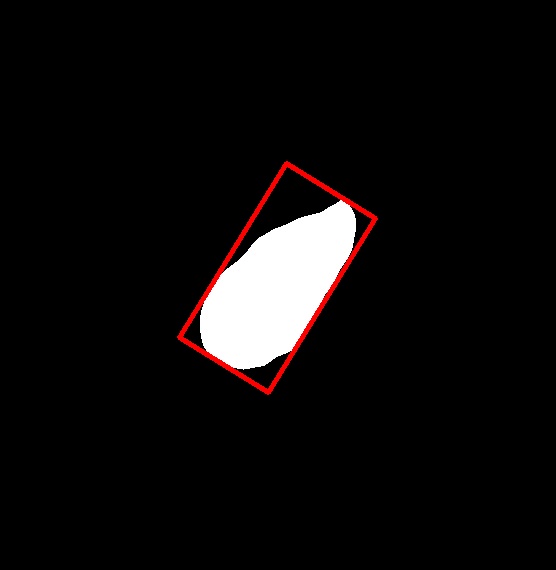

Supplement: Supplementary 1 — File S1 [file plantphenomics.0158.f1.zip › supplementary/0721_027_31.jpg]

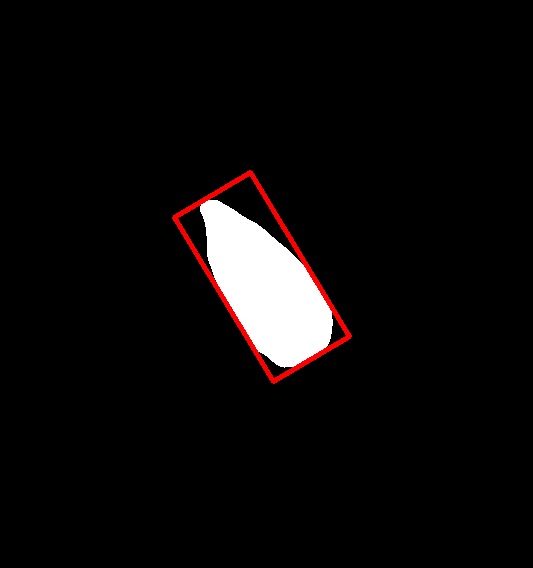

Supplement: Supplementary 1 — File S1 [file plantphenomics.0158.f1.zip › supplementary/0721_027_40.jpg]

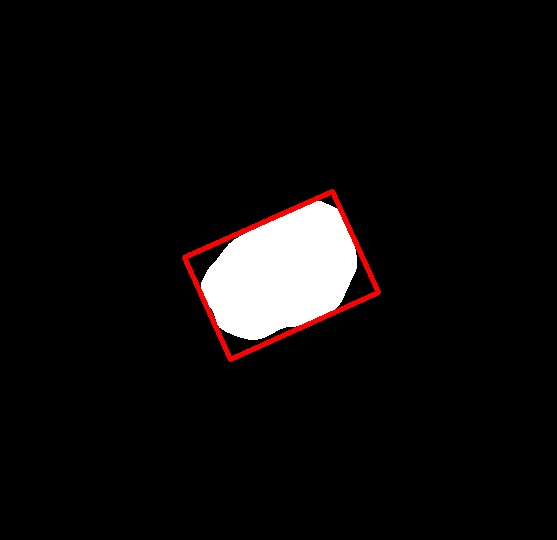

Supplement: Supplementary 1 — File S1 [file plantphenomics.0158.f1.zip › supplementary/0721_028_13.jpg]

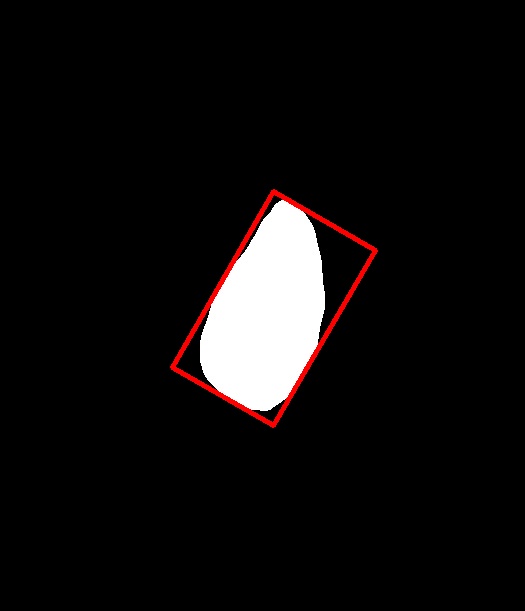

Supplement: Supplementary 1 — File S1 [file plantphenomics.0158.f1.zip › supplementary/0721_028_135.jpg]

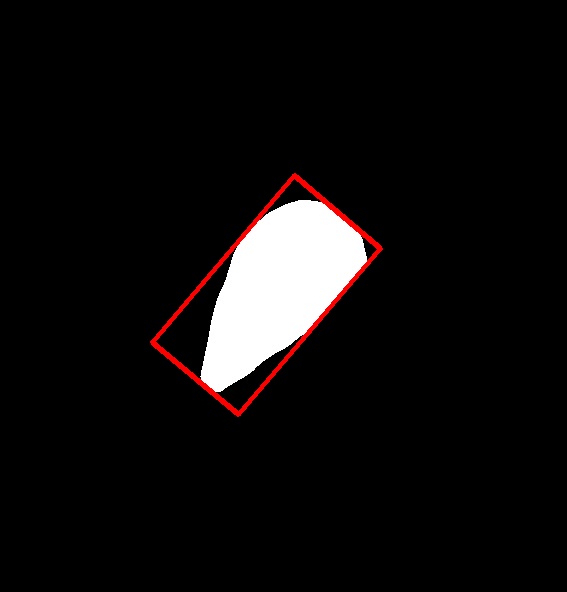

Supplement: Supplementary 1 — File S1 [file plantphenomics.0158.f1.zip › supplementary/0721_028_79.jpg]

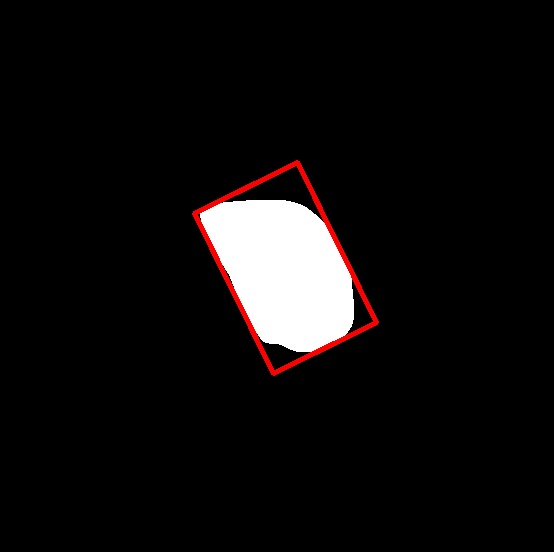

Supplement: Supplementary 1 — File S1 [file plantphenomics.0158.f1.zip › supplementary/0721_028_85.jpg]

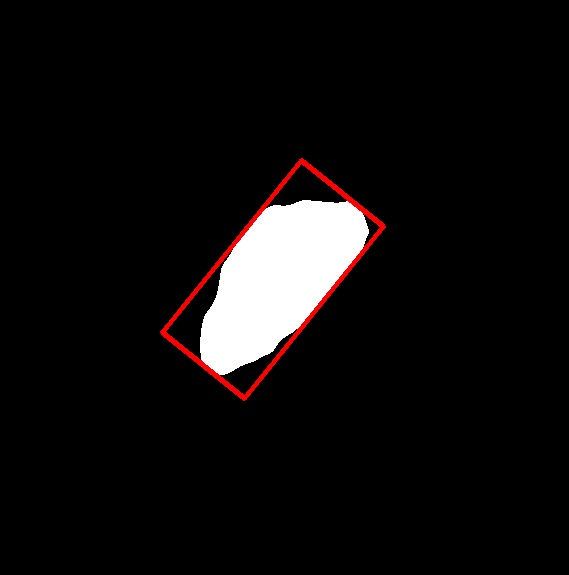

Supplement: Supplementary 1 — File S1 [file plantphenomics.0158.f1.zip › supplementary/0721_028_94.jpg]

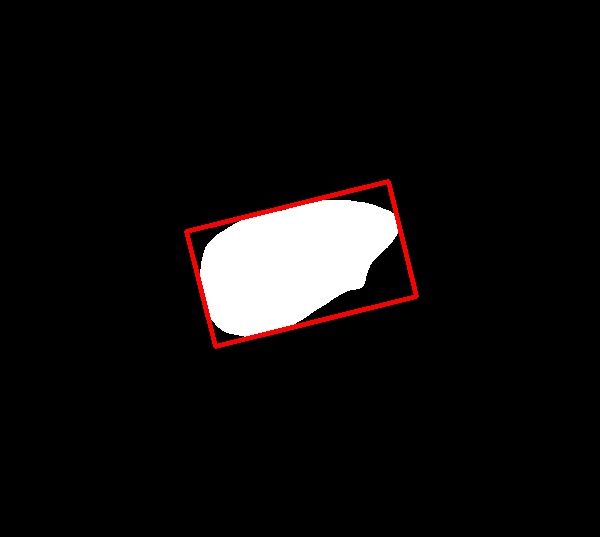

Supplement: Supplementary 1 — File S1 [file plantphenomics.0158.f1.zip › supplementary/0721_029_13.jpg]

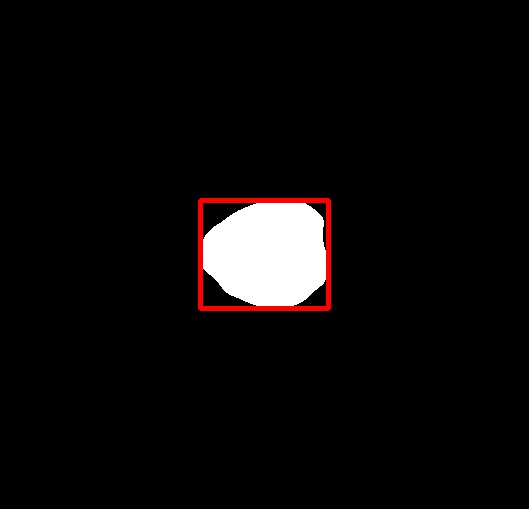

Supplement: Supplementary 1 — File S1 [file plantphenomics.0158.f1.zip › supplementary/0721_029_2.jpg]

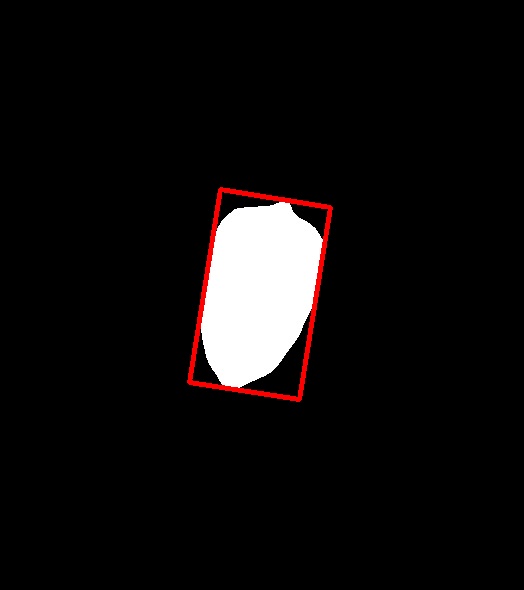

Supplement: Supplementary 1 — File S1 [file plantphenomics.0158.f1.zip › supplementary/0721_029_24.jpg]

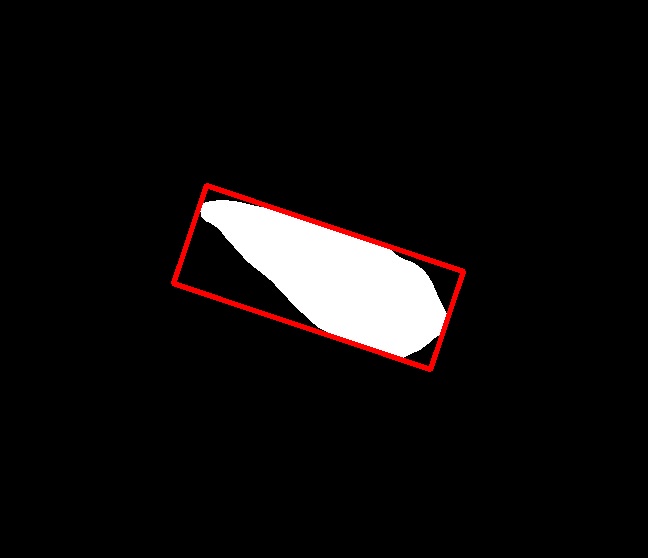

Supplement: Supplementary 1 — File S1 [file plantphenomics.0158.f1.zip › supplementary/0721_029_36.jpg]

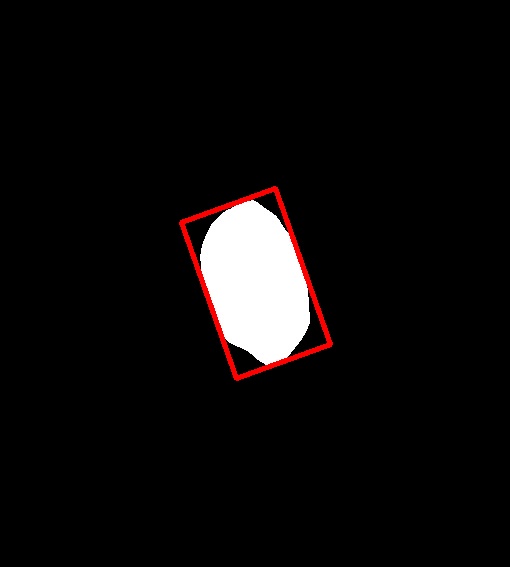

Supplement: Supplementary 1 — File S1 [file plantphenomics.0158.f1.zip › supplementary/0721_029_39.jpg]

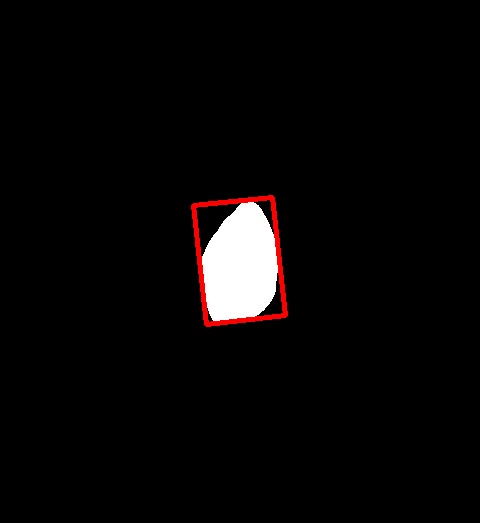

Supplement: Supplementary 1 — File S1 [file plantphenomics.0158.f1.zip › supplementary/0721_030_152.jpg]

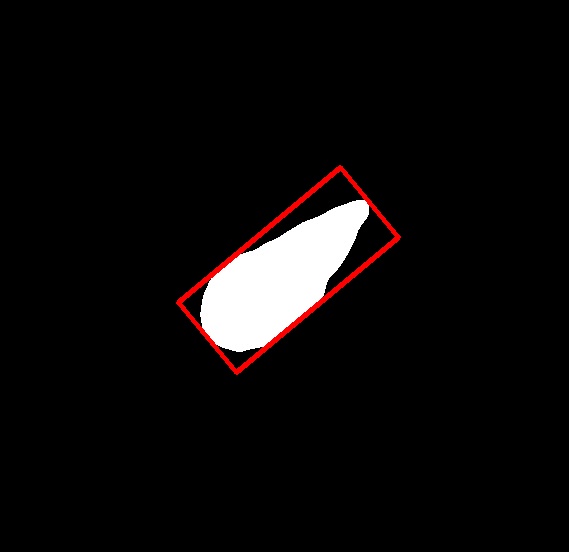

Supplement: Supplementary 1 — File S1 [file plantphenomics.0158.f1.zip › supplementary/0721_030_2.jpg]

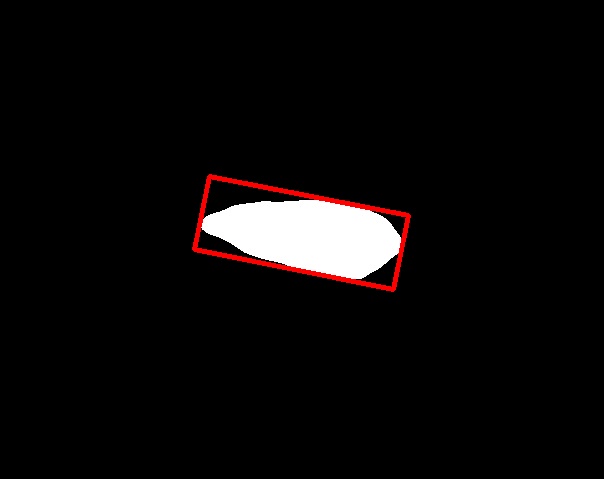

Supplement: Supplementary 1 — File S1 [file plantphenomics.0158.f1.zip › supplementary/0721_030_210.jpg]

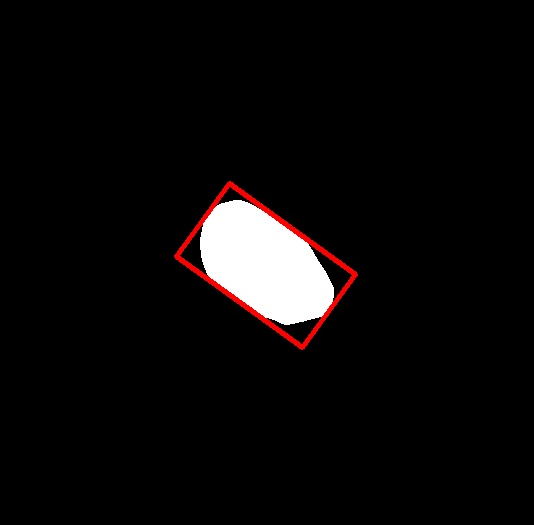

Supplement: Supplementary 1 — File S1 [file plantphenomics.0158.f1.zip › supplementary/0721_030_33.jpg]

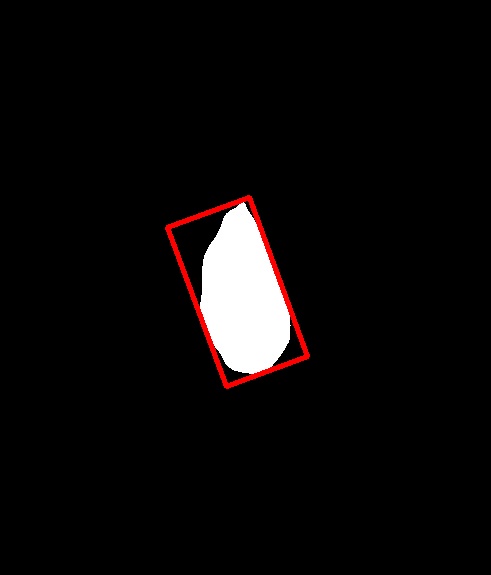

Supplement: Supplementary 1 — File S1 [file plantphenomics.0158.f1.zip › supplementary/0721_030_90.jpg]

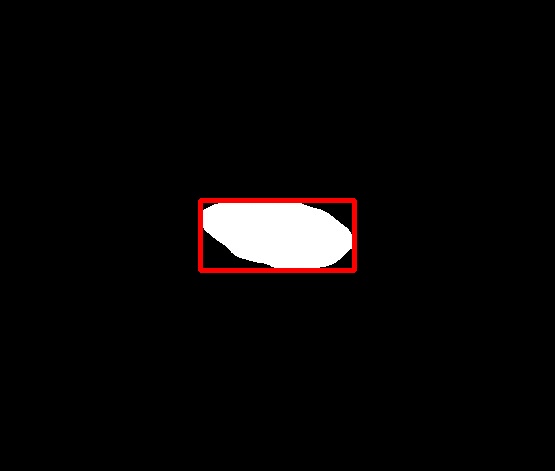

Supplement: Supplementary 1 — File S1 [file plantphenomics.0158.f1.zip › supplementary/0721_031_124.jpg]

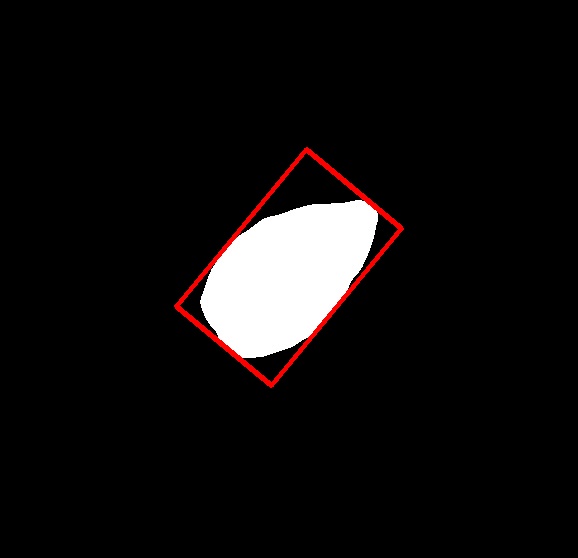

Supplement: Supplementary 1 — File S1 [file plantphenomics.0158.f1.zip › supplementary/0721_031_13.jpg]

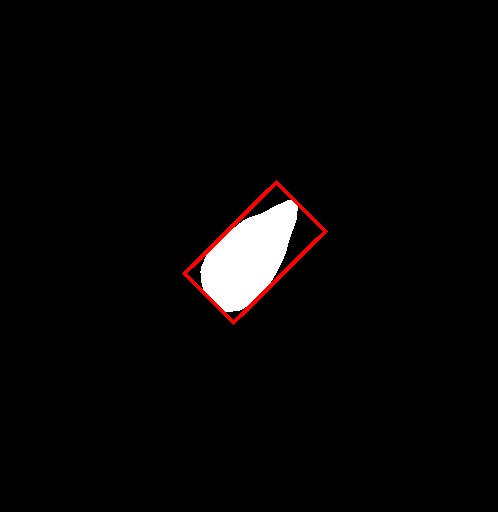

Supplement: Supplementary 1 — File S1 [file plantphenomics.0158.f1.zip › supplementary/0721_031_154.jpg]

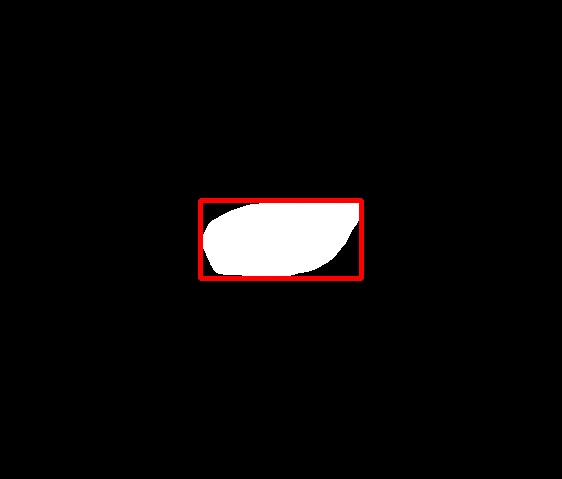

Supplement: Supplementary 1 — File S1 [file plantphenomics.0158.f1.zip › supplementary/0721_031_17.jpg]

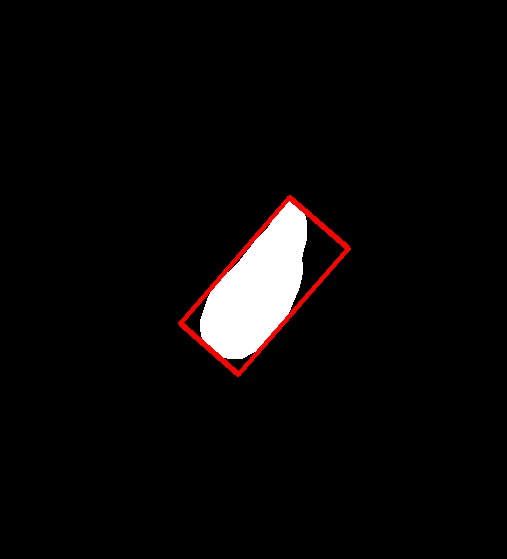

Supplement: Supplementary 1 — File S1 [file plantphenomics.0158.f1.zip › supplementary/0721_031_83.jpg]

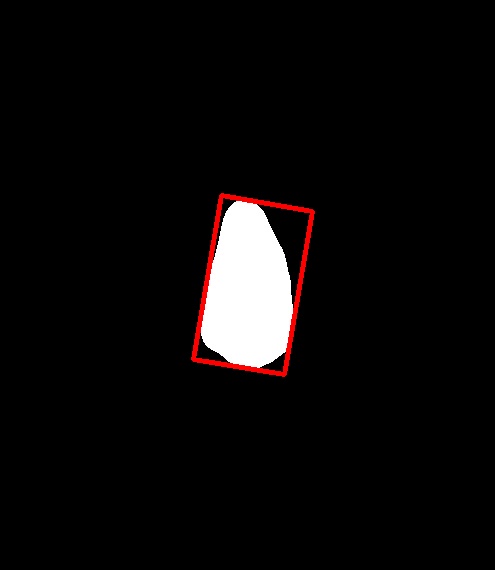

Supplement: Supplementary 1 — File S1 [file plantphenomics.0158.f1.zip › supplementary/0721_032_113.jpg]

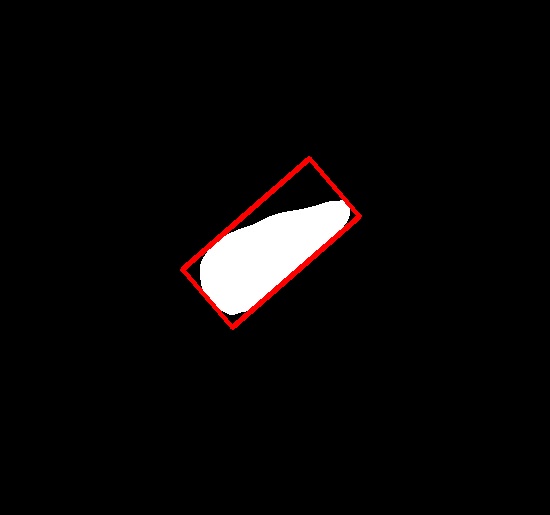

Supplement: Supplementary 1 — File S1 [file plantphenomics.0158.f1.zip › supplementary/0721_032_3.jpg]

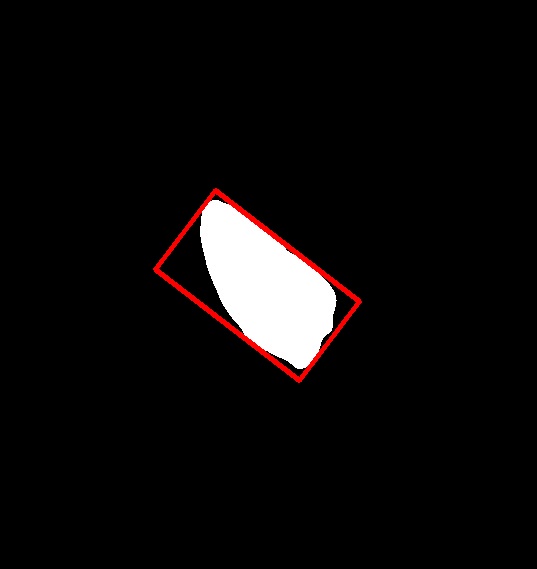

Supplement: Supplementary 1 — File S1 [file plantphenomics.0158.f1.zip › supplementary/0721_032_43.jpg]

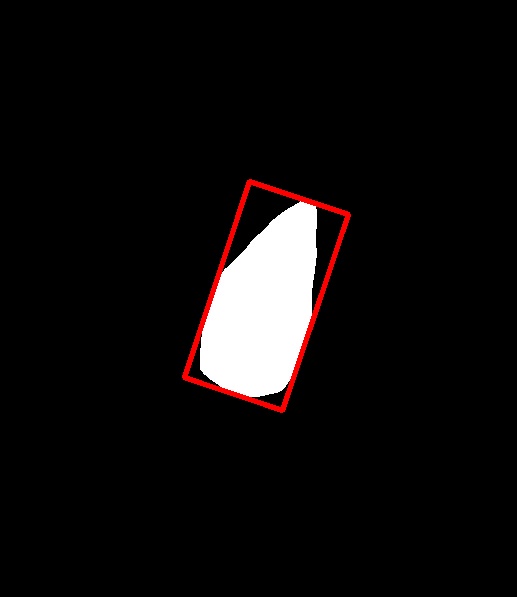

Supplement: Supplementary 1 — File S1 [file plantphenomics.0158.f1.zip › supplementary/0721_032_57.jpg]

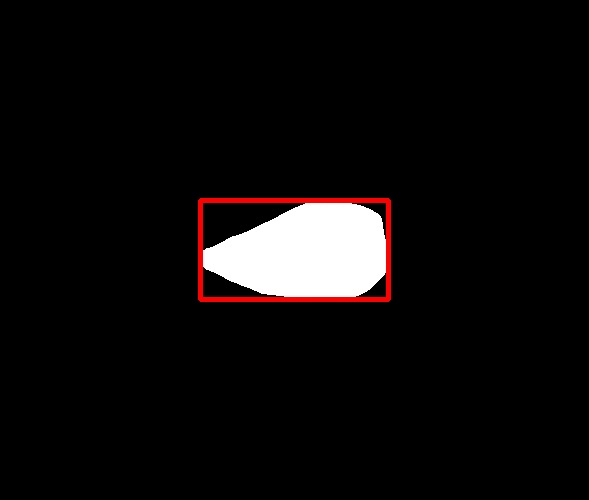

Supplement: Supplementary 1 — File S1 [file plantphenomics.0158.f1.zip › supplementary/0721_032_98.jpg]

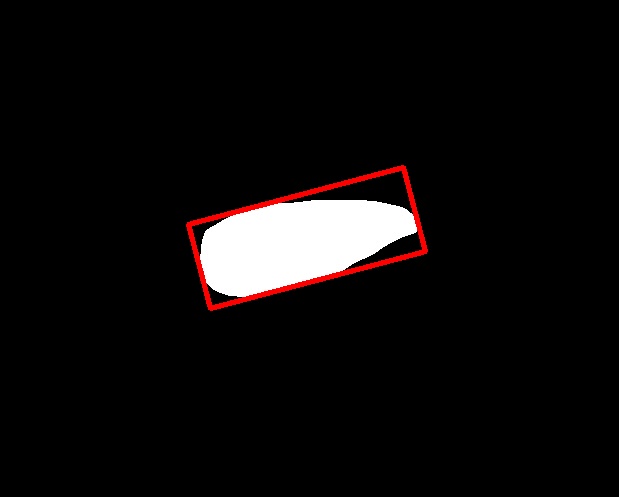

Supplement: Supplementary 1 — File S1 [file plantphenomics.0158.f1.zip › supplementary/0721_033_116.jpg]

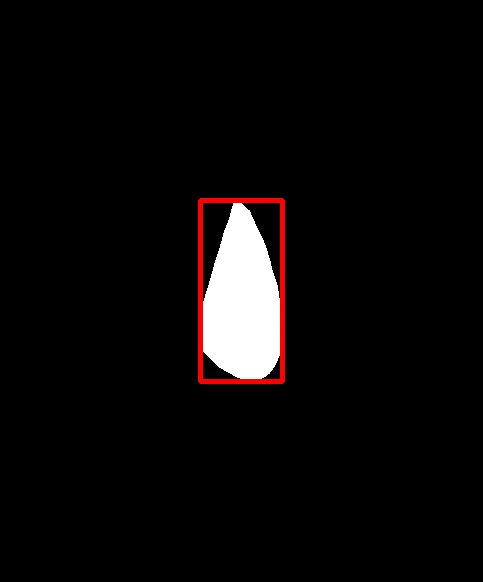

Supplement: Supplementary 1 — File S1 [file plantphenomics.0158.f1.zip › supplementary/0721_033_122.jpg]

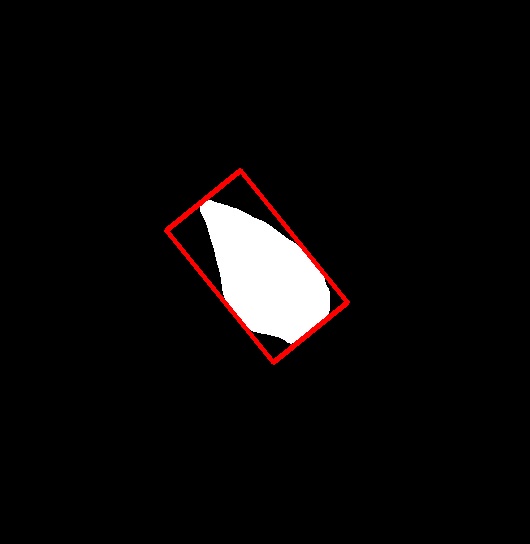

Supplement: Supplementary 1 — File S1 [file plantphenomics.0158.f1.zip › supplementary/0721_033_166.jpg]

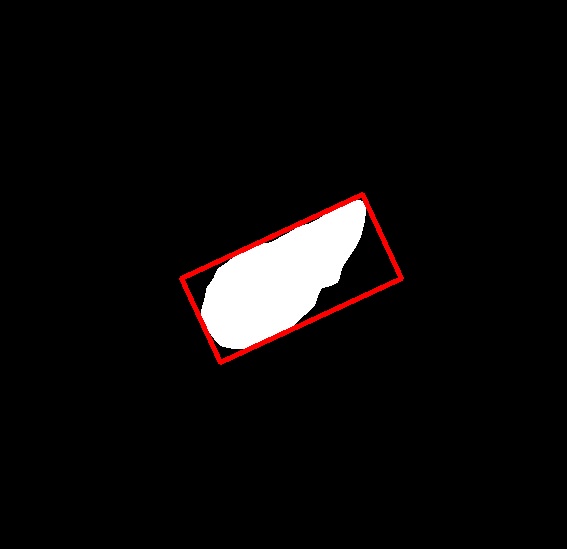

Supplement: Supplementary 1 — File S1 [file plantphenomics.0158.f1.zip › supplementary/0721_033_173.jpg]

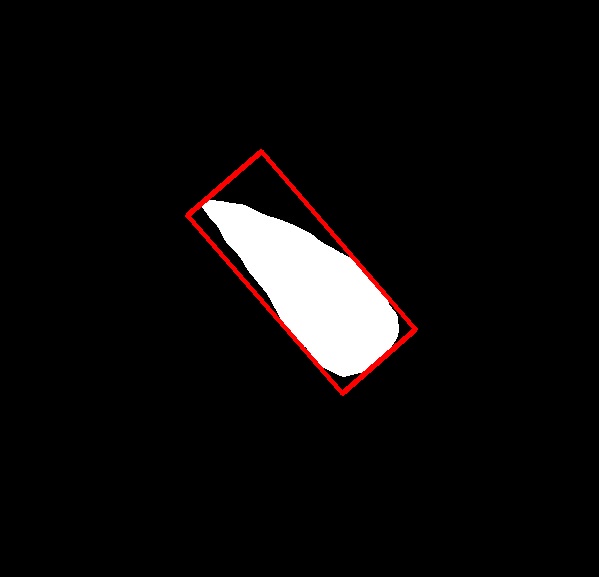

Supplement: Supplementary 1 — File S1 [file plantphenomics.0158.f1.zip › supplementary/0721_033_75.jpg]

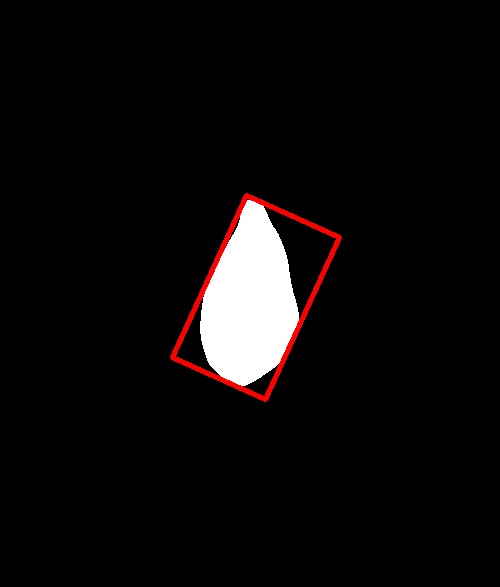

Supplement: Supplementary 1 — File S1 [file plantphenomics.0158.f1.zip › supplementary/0721_034_0.jpg]

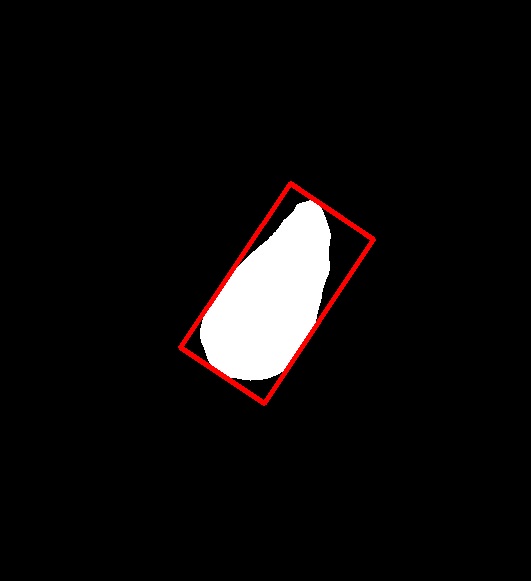

Supplement: Supplementary 1 — File S1 [file plantphenomics.0158.f1.zip › supplementary/0721_034_18.jpg]

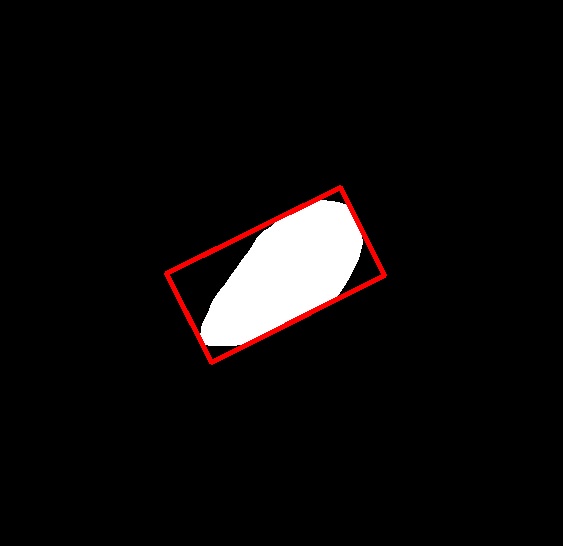

Supplement: Supplementary 1 — File S1 [file plantphenomics.0158.f1.zip › supplementary/0721_034_199.jpg]

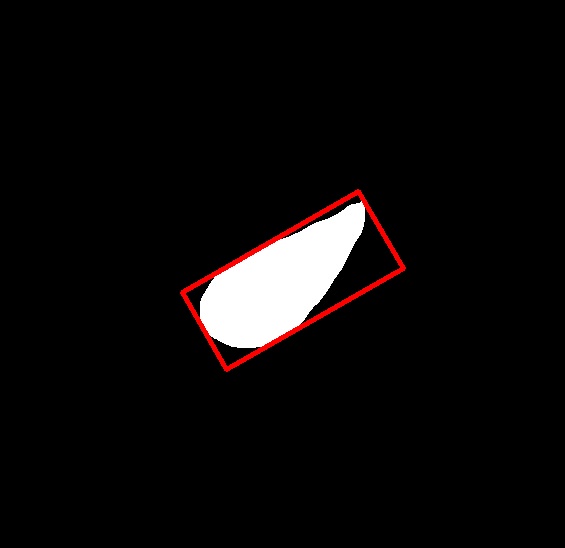

Supplement: Supplementary 1 — File S1 [file plantphenomics.0158.f1.zip › supplementary/0721_034_216.jpg]

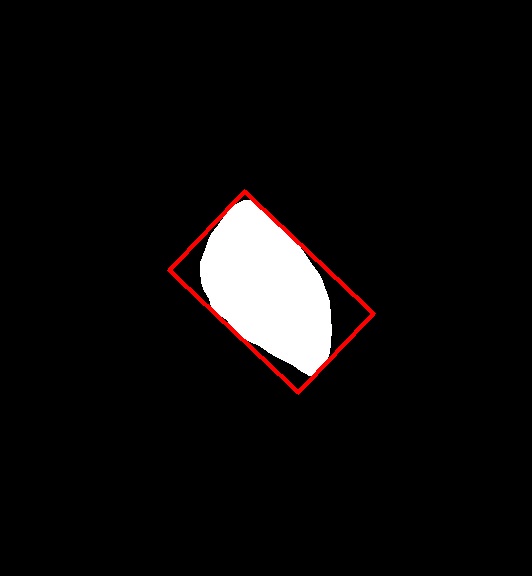

Supplement: Supplementary 1 — File S1 [file plantphenomics.0158.f1.zip › supplementary/0721_034_90.jpg]

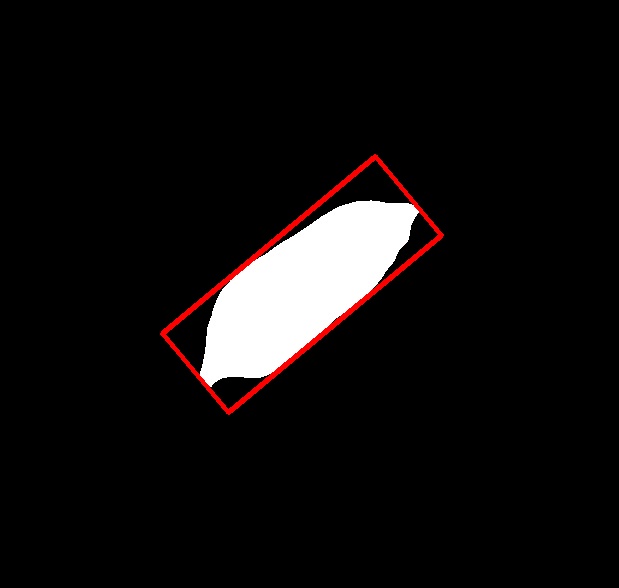

Supplement: Supplementary 1 — File S1 [file plantphenomics.0158.f1.zip › supplementary/0721_035_128.jpg]

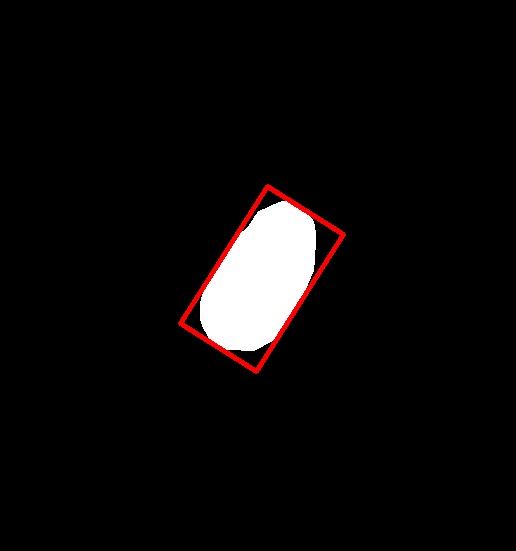

Supplement: Supplementary 1 — File S1 [file plantphenomics.0158.f1.zip › supplementary/0721_035_145.jpg]

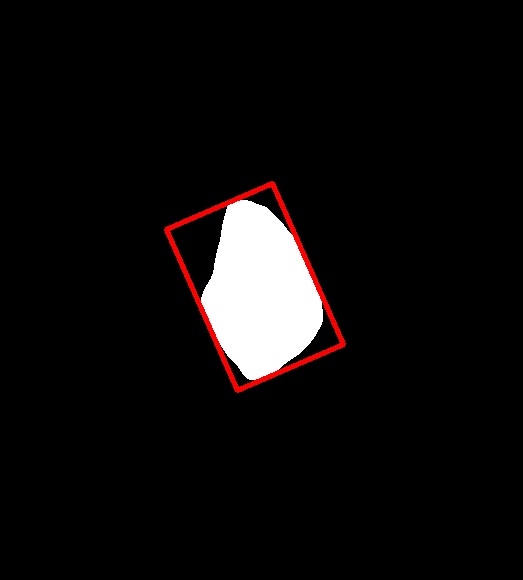

Supplement: Supplementary 1 — File S1 [file plantphenomics.0158.f1.zip › supplementary/0721_035_165.jpg]

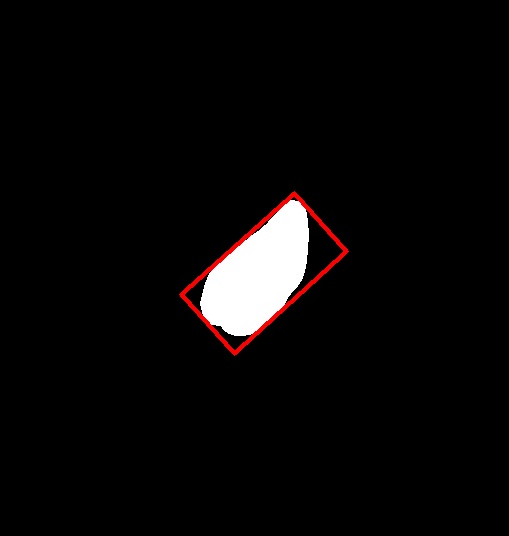

Supplement: Supplementary 1 — File S1 [file plantphenomics.0158.f1.zip › supplementary/0721_035_38.jpg]

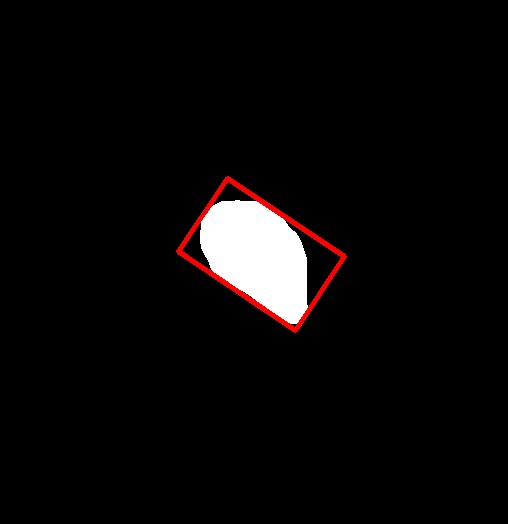

Supplement: Supplementary 1 — File S1 [file plantphenomics.0158.f1.zip › supplementary/0721_035_8.jpg]

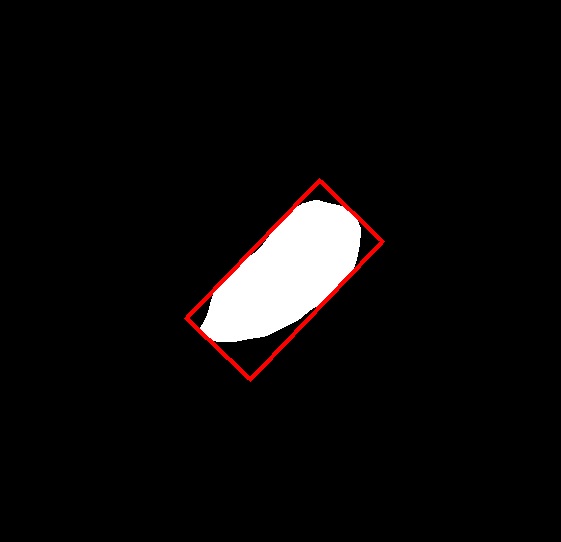

Supplement: Supplementary 1 — File S1 [file plantphenomics.0158.f1.zip › supplementary/0721_036_107.jpg]

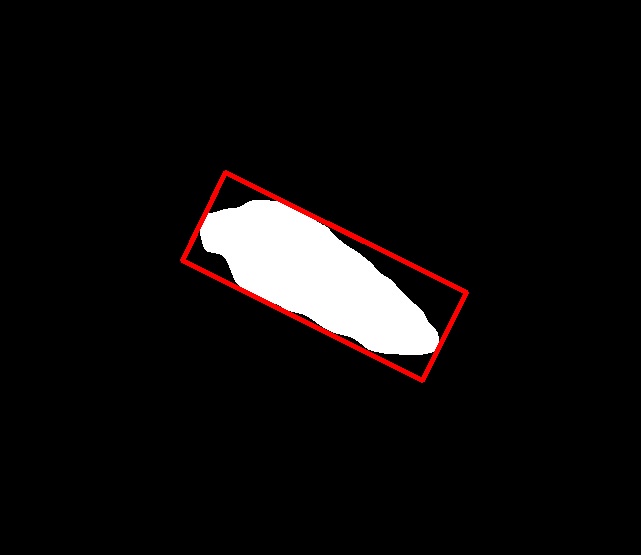

Supplement: Supplementary 1 — File S1 [file plantphenomics.0158.f1.zip › supplementary/0721_036_111.jpg]

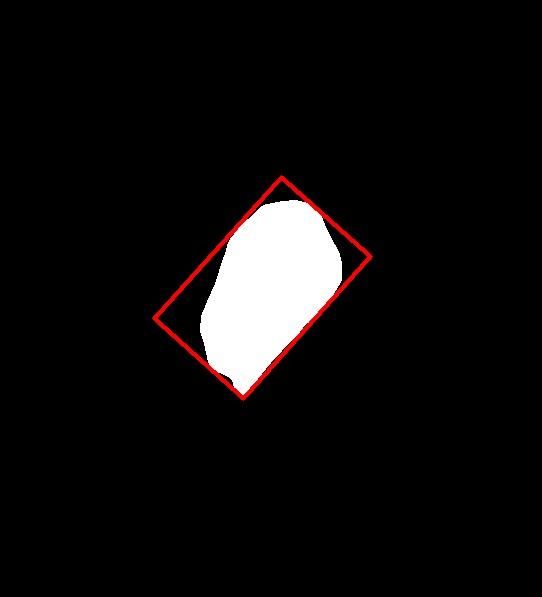

Supplement: Supplementary 1 — File S1 [file plantphenomics.0158.f1.zip › supplementary/0721_036_76.jpg]

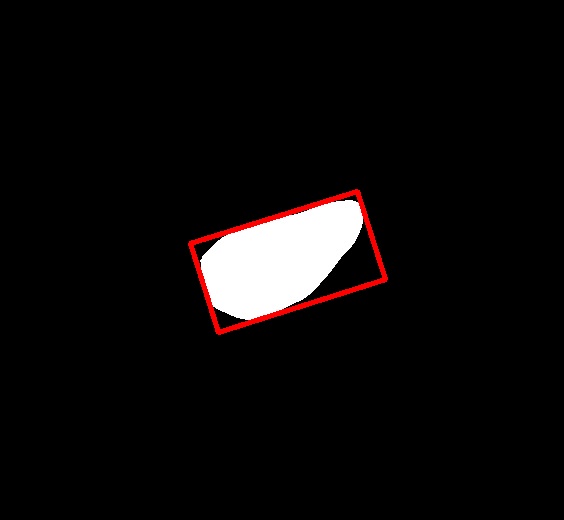

Supplement: Supplementary 1 — File S1 [file plantphenomics.0158.f1.zip › supplementary/0721_036_83.jpg]

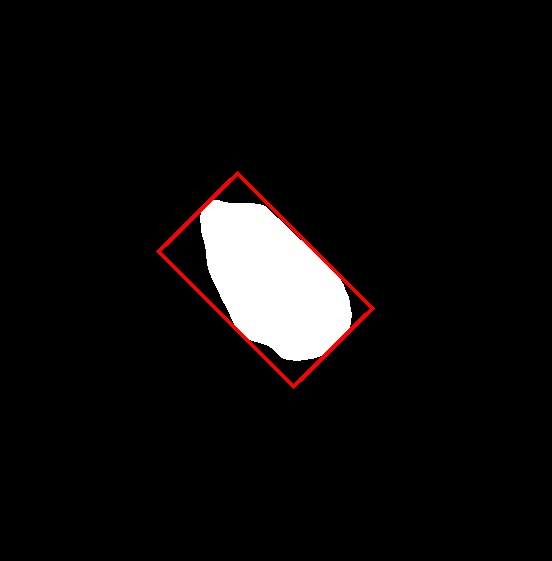

Supplement: Supplementary 1 — File S1 [file plantphenomics.0158.f1.zip › supplementary/0721_036_87.jpg]

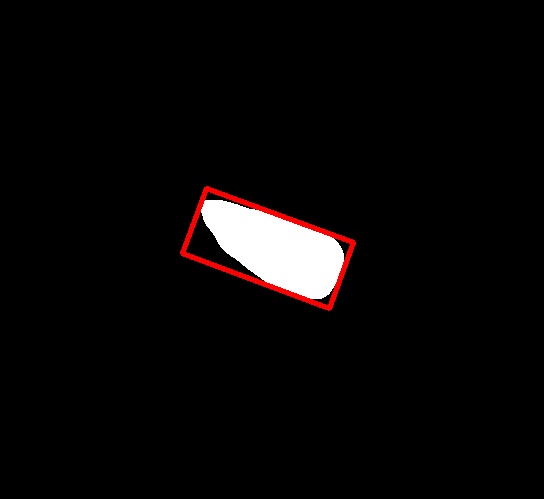

Supplement: Supplementary 1 — File S1 [file plantphenomics.0158.f1.zip › supplementary/0721_037_12.jpg]

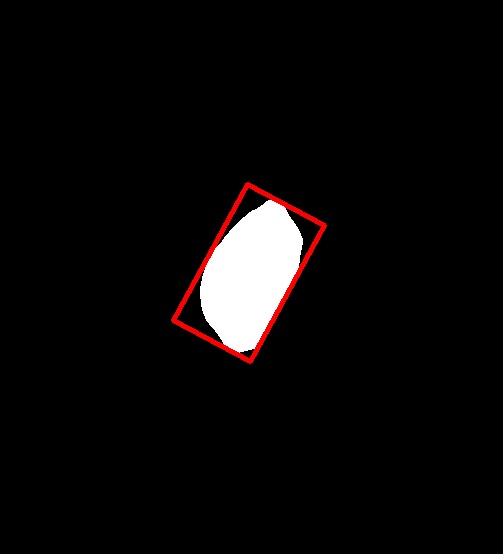

Supplement: Supplementary 1 — File S1 [file plantphenomics.0158.f1.zip › supplementary/0721_037_136.jpg]

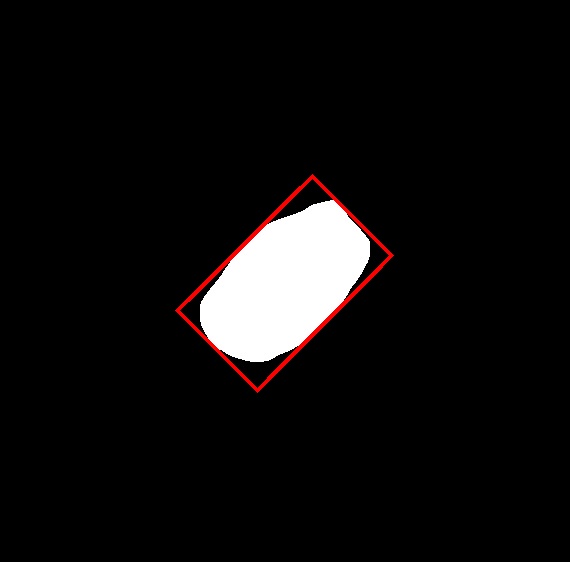

Supplement: Supplementary 1 — File S1 [file plantphenomics.0158.f1.zip › supplementary/0721_037_176.jpg]

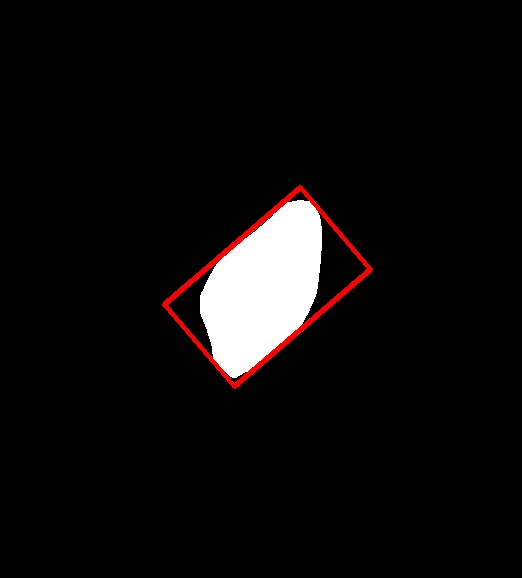

Supplement: Supplementary 1 — File S1 [file plantphenomics.0158.f1.zip › supplementary/0721_037_199.jpg]

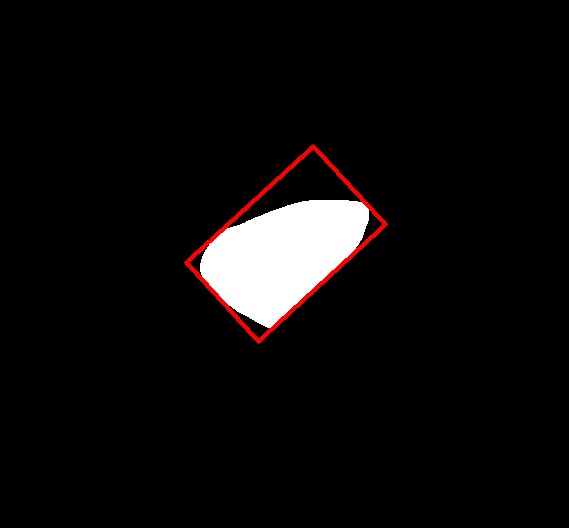

Supplement: Supplementary 1 — File S1 [file plantphenomics.0158.f1.zip › supplementary/0721_037_2.jpg]

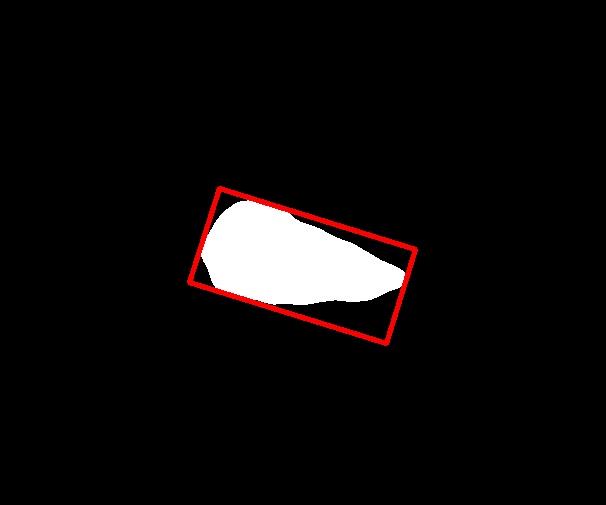

Supplement: Supplementary 1 — File S1 [file plantphenomics.0158.f1.zip › supplementary/0721_038_101.jpg]

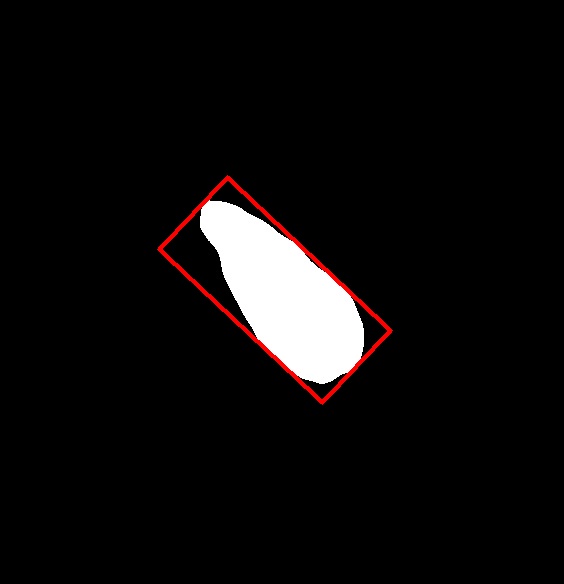

Supplement: Supplementary 1 — File S1 [file plantphenomics.0158.f1.zip › supplementary/0721_038_140.jpg]

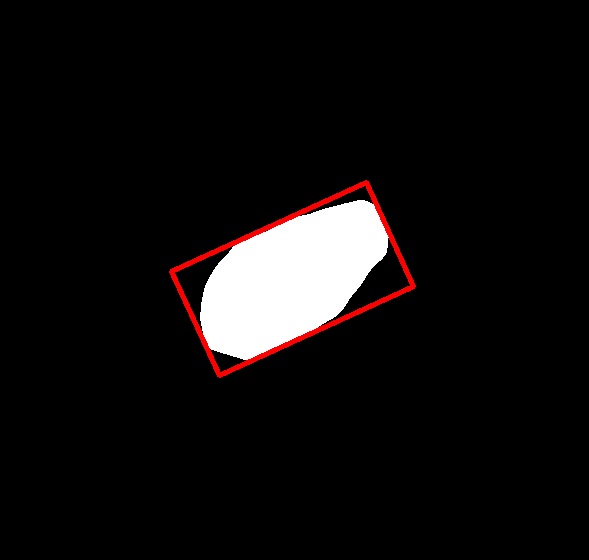

Supplement: Supplementary 1 — File S1 [file plantphenomics.0158.f1.zip › supplementary/0721_038_35.jpg]

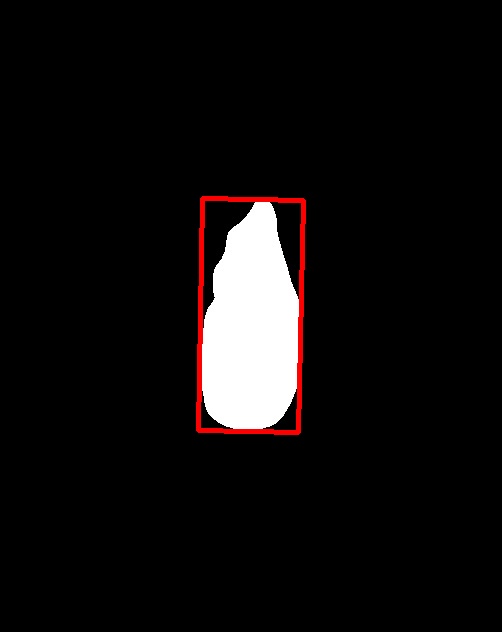

Supplement: Supplementary 1 — File S1 [file plantphenomics.0158.f1.zip › supplementary/0721_038_58.jpg]

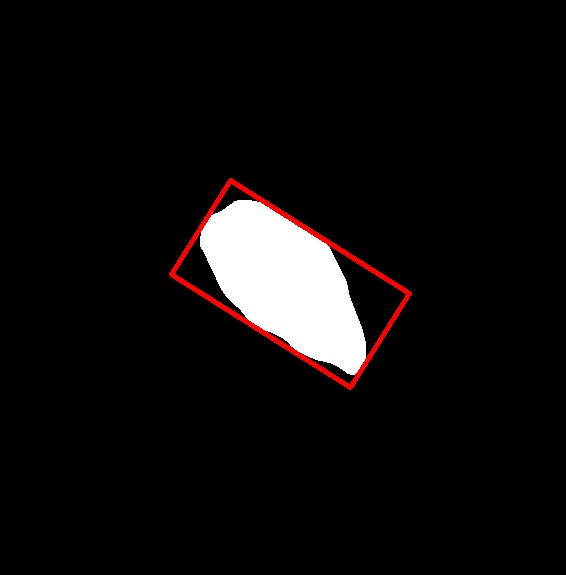

Supplement: Supplementary 1 — File S1 [file plantphenomics.0158.f1.zip › supplementary/0721_038_62.jpg]

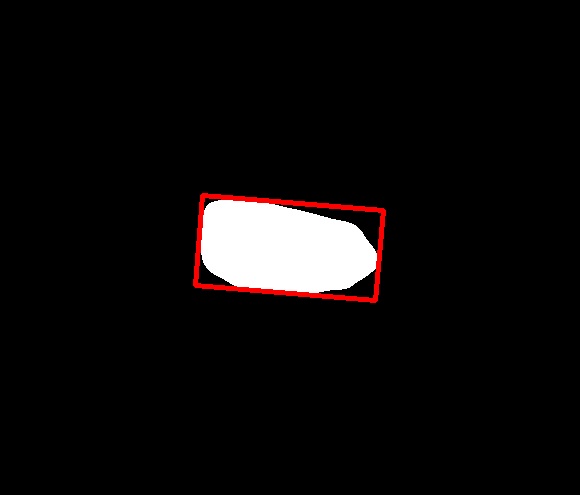

Supplement: Supplementary 1 — File S1 [file plantphenomics.0158.f1.zip › supplementary/0721_039_0.jpg]

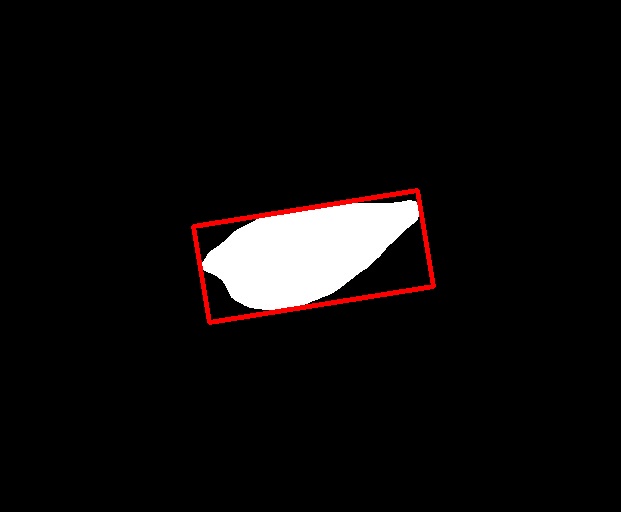

Supplement: Supplementary 1 — File S1 [file plantphenomics.0158.f1.zip › supplementary/0721_039_120.jpg]

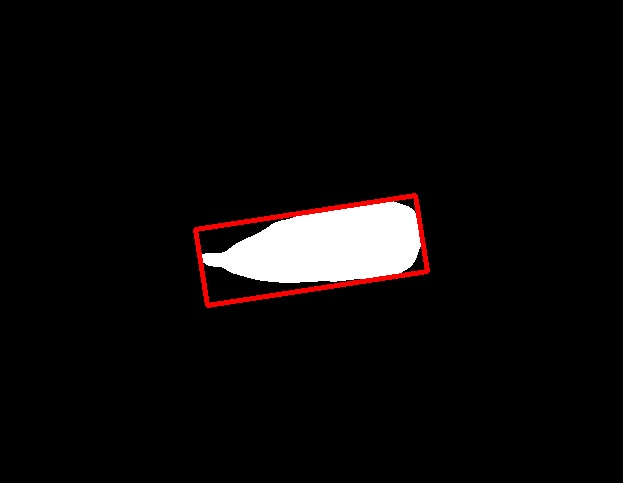

Supplement: Supplementary 1 — File S1 [file plantphenomics.0158.f1.zip › supplementary/0721_039_160.jpg]

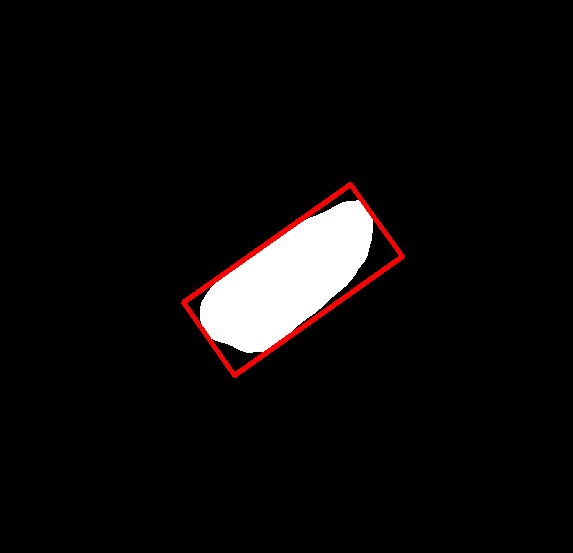

Supplement: Supplementary 1 — File S1 [file plantphenomics.0158.f1.zip › supplementary/0721_039_40.jpg]

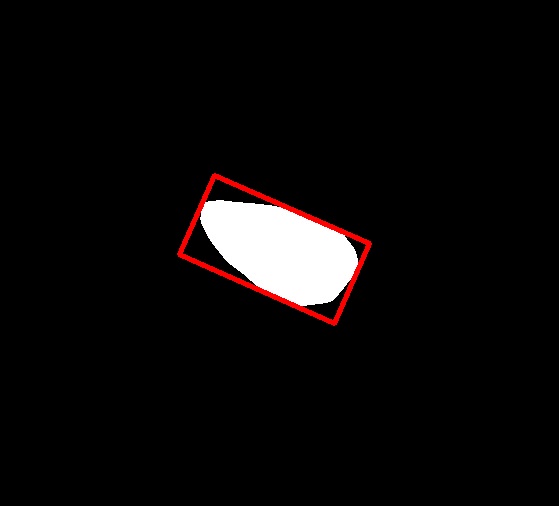

Supplement: Supplementary 1 — File S1 [file plantphenomics.0158.f1.zip › supplementary/0721_039_62.jpg]

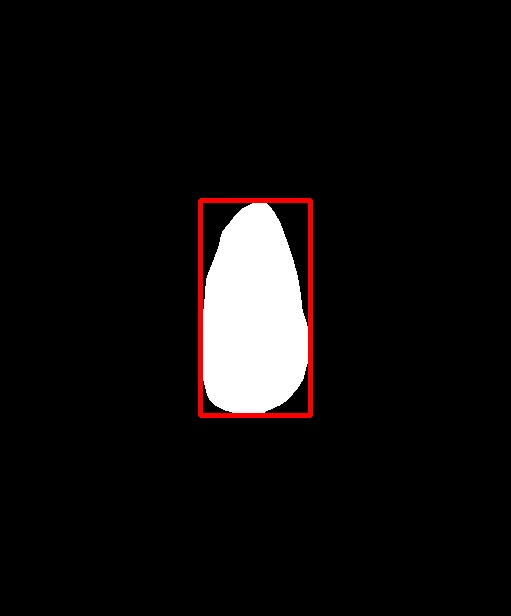

Supplement: Supplementary 1 — File S1 [file plantphenomics.0158.f1.zip › supplementary/0721_040_153.jpg]

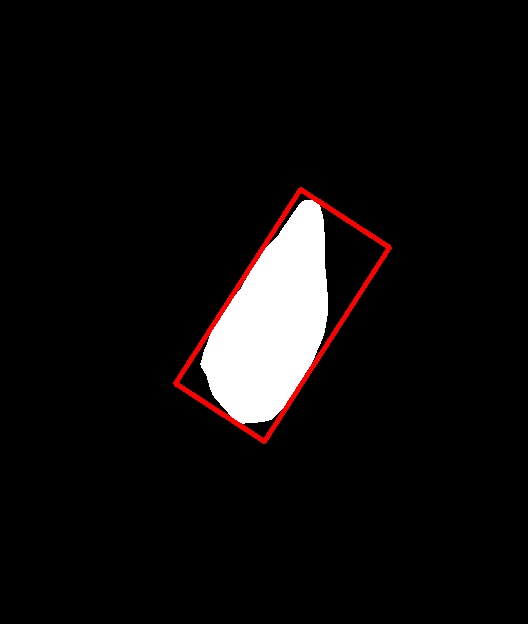

Supplement: Supplementary 1 — File S1 [file plantphenomics.0158.f1.zip › supplementary/0721_040_154.jpg]

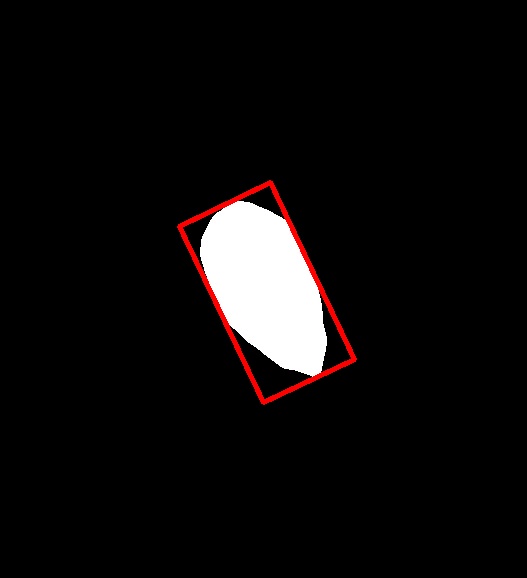

Supplement: Supplementary 1 — File S1 [file plantphenomics.0158.f1.zip › supplementary/0721_040_158.jpg]

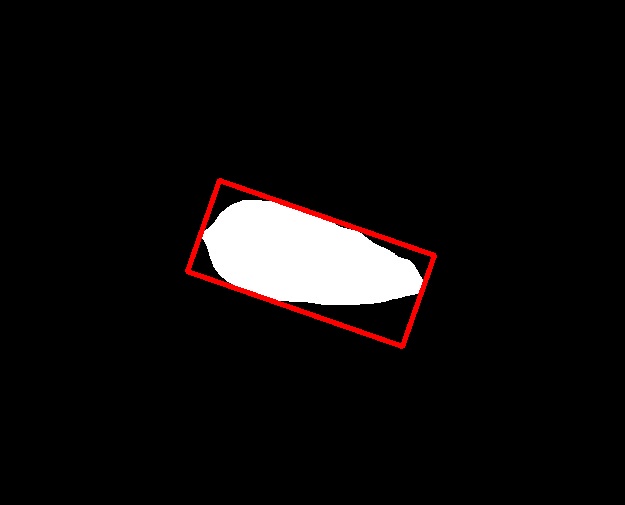

Supplement: Supplementary 1 — File S1 [file plantphenomics.0158.f1.zip › supplementary/0721_040_191.jpg]

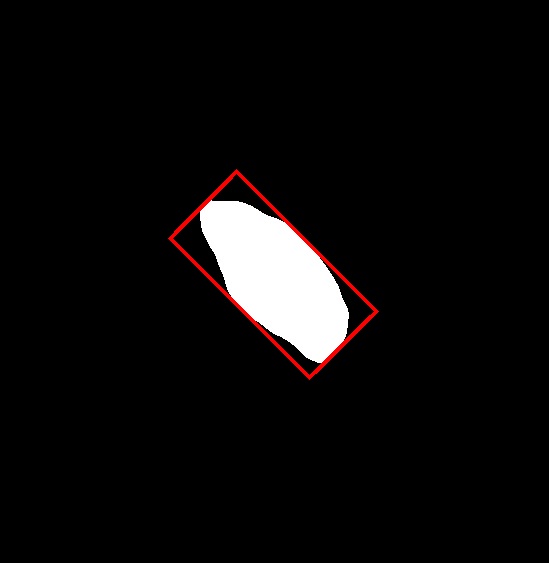

Supplement: Supplementary 1 — File S1 [file plantphenomics.0158.f1.zip › supplementary/0721_040_199.jpg]
