# Supplementary figures and images for: Time-Series Field Phenotyping of Soybean Growth Analysis by Combining Multimodal Deep Learning and Dynamic Modeling (part 1 of 2)
Source: Plant Phenomics. 2024 Mar 20;6:0158. doi: 10.34133/plantphenomics.0158 (PMC10959008; doi:10.34133/plantphenomics.0158)

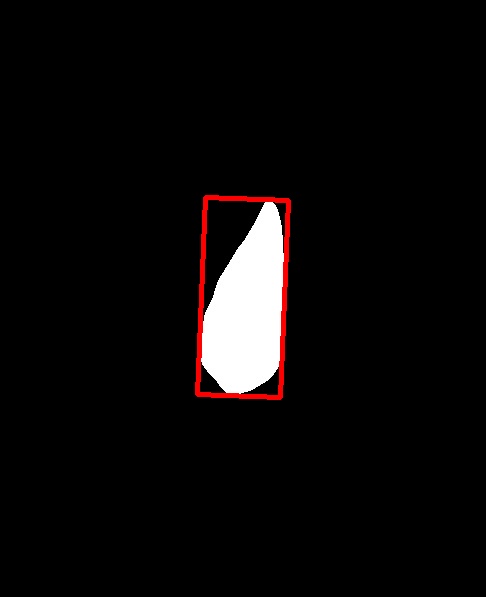

Supplement: Supplementary 1 — File S1 [file plantphenomics.0158.f1.zip › supplementary/0721_001_115.jpg]

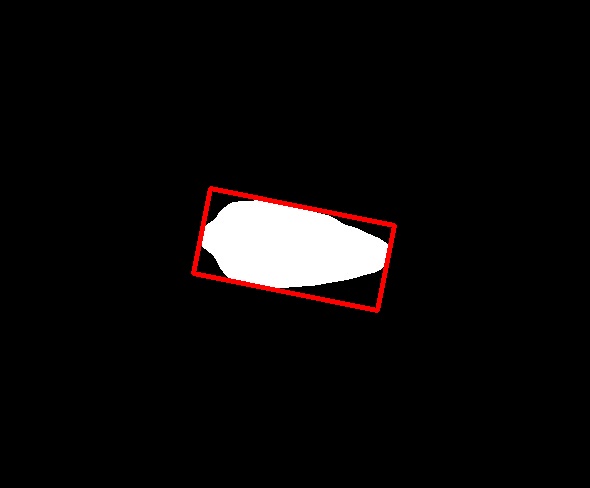

Supplement: Supplementary 1 — File S1 [file plantphenomics.0158.f1.zip › supplementary/0721_001_126.jpg]

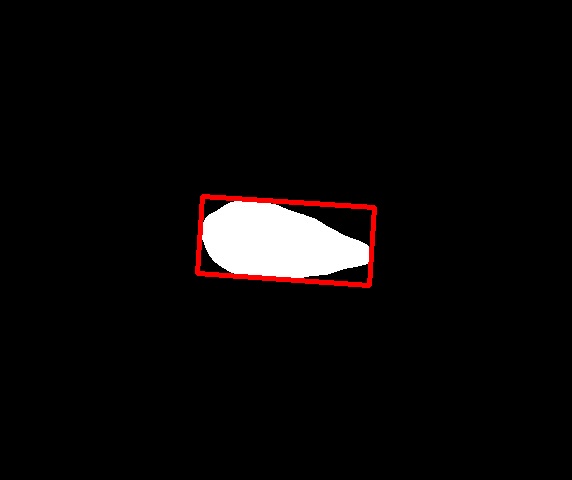

Supplement: Supplementary 1 — File S1 [file plantphenomics.0158.f1.zip › supplementary/0721_001_13.jpg]

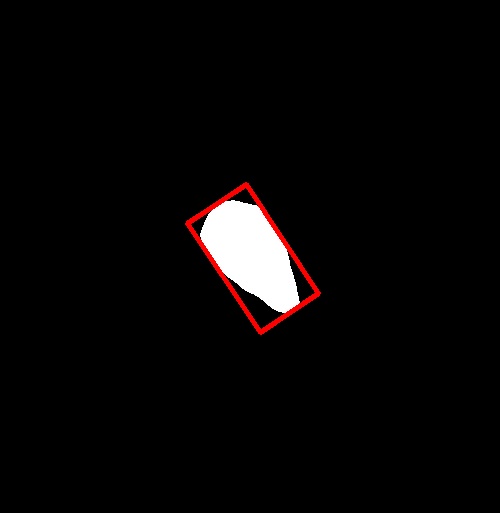

Supplement: Supplementary 1 — File S1 [file plantphenomics.0158.f1.zip › supplementary/0721_001_15.jpg]

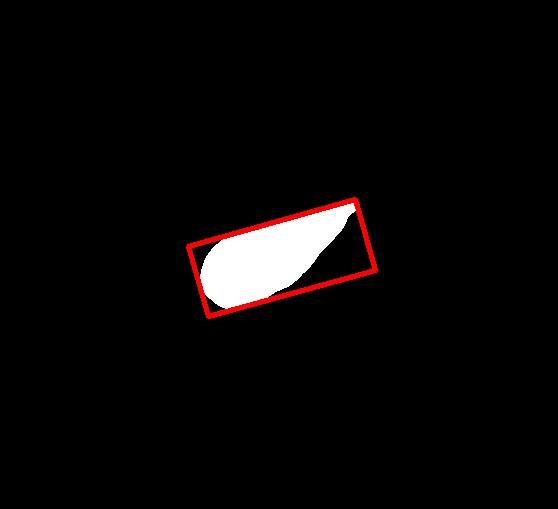

Supplement: Supplementary 1 — File S1 [file plantphenomics.0158.f1.zip › supplementary/0721_001_74.jpg]

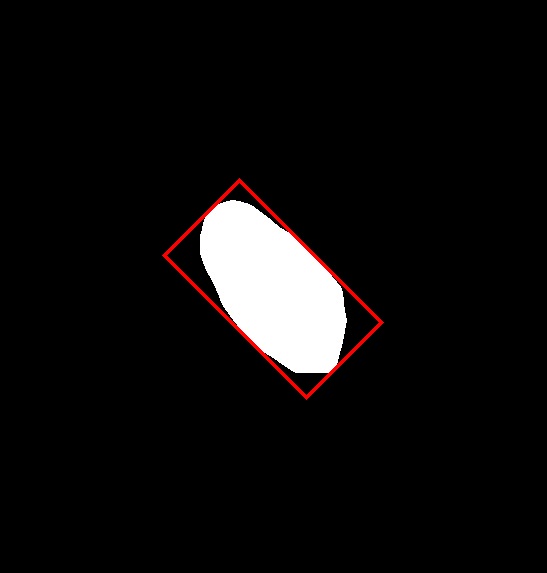

Supplement: Supplementary 1 — File S1 [file plantphenomics.0158.f1.zip › supplementary/0721_002_0.jpg]

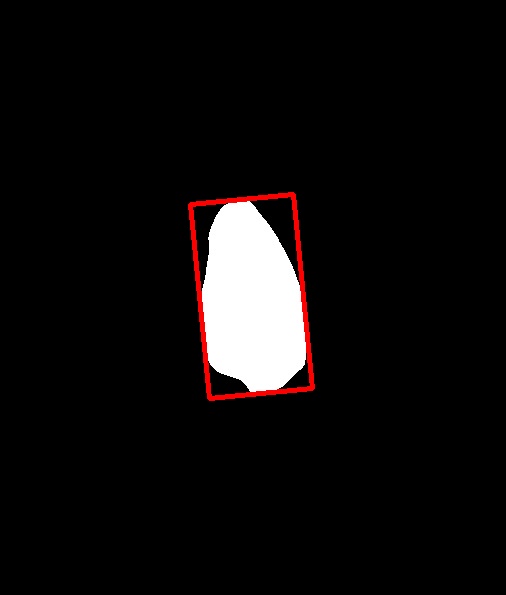

Supplement: Supplementary 1 — File S1 [file plantphenomics.0158.f1.zip › supplementary/0721_002_15.jpg]

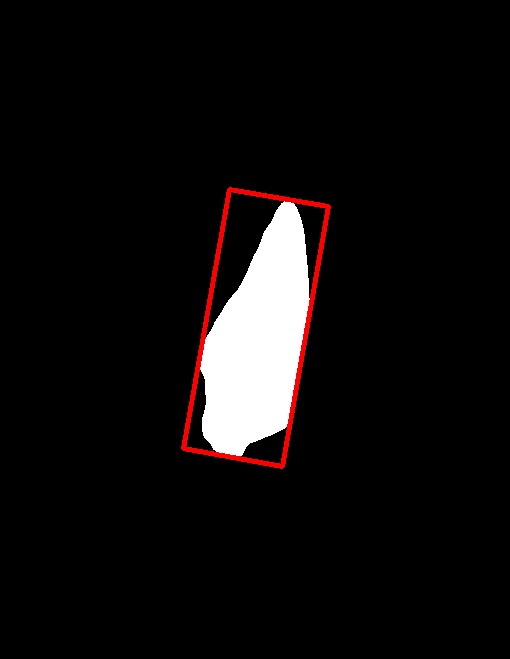

Supplement: Supplementary 1 — File S1 [file plantphenomics.0158.f1.zip › supplementary/0721_002_33.jpg]

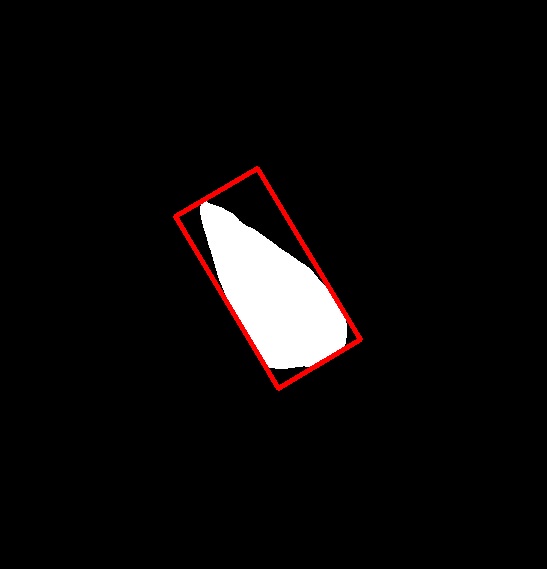

Supplement: Supplementary 1 — File S1 [file plantphenomics.0158.f1.zip › supplementary/0721_002_35.jpg]

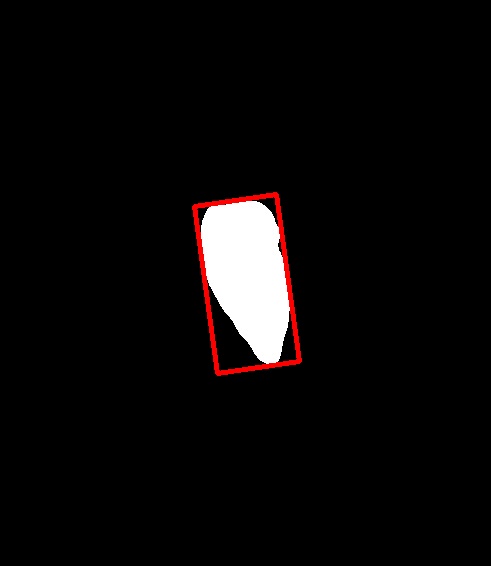

Supplement: Supplementary 1 — File S1 [file plantphenomics.0158.f1.zip › supplementary/0721_002_42.jpg]

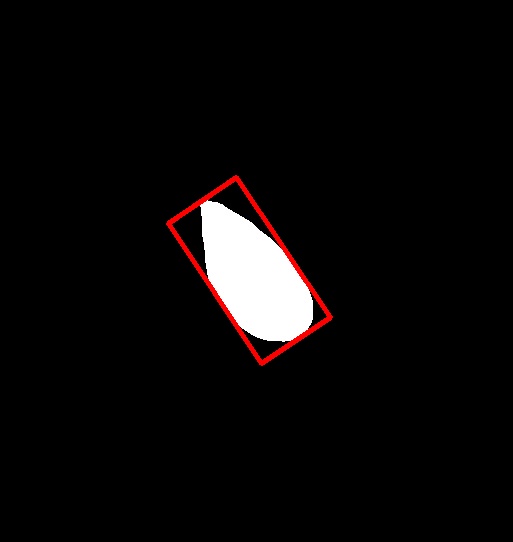

Supplement: Supplementary 1 — File S1 [file plantphenomics.0158.f1.zip › supplementary/0721_003_24.jpg]

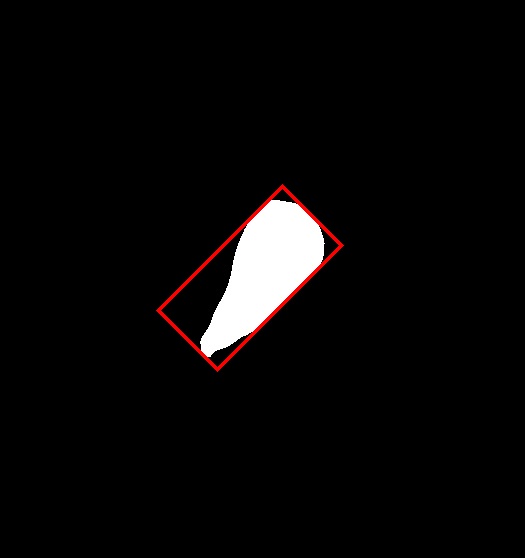

Supplement: Supplementary 1 — File S1 [file plantphenomics.0158.f1.zip › supplementary/0721_003_35.jpg]

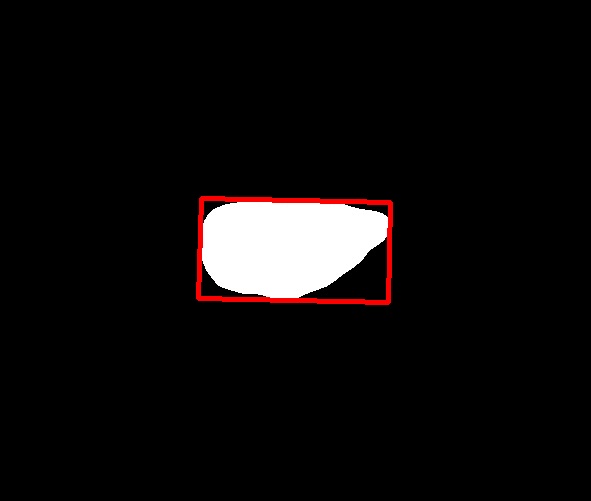

Supplement: Supplementary 1 — File S1 [file plantphenomics.0158.f1.zip › supplementary/0721_003_46.jpg]

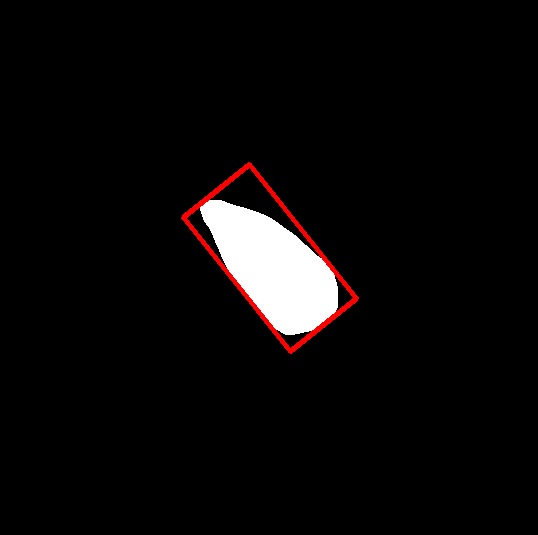

Supplement: Supplementary 1 — File S1 [file plantphenomics.0158.f1.zip › supplementary/0721_003_5.jpg]

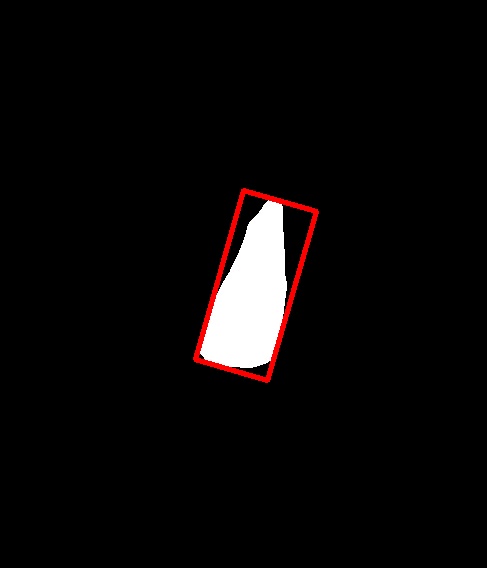

Supplement: Supplementary 1 — File S1 [file plantphenomics.0158.f1.zip › supplementary/0721_003_90.jpg]

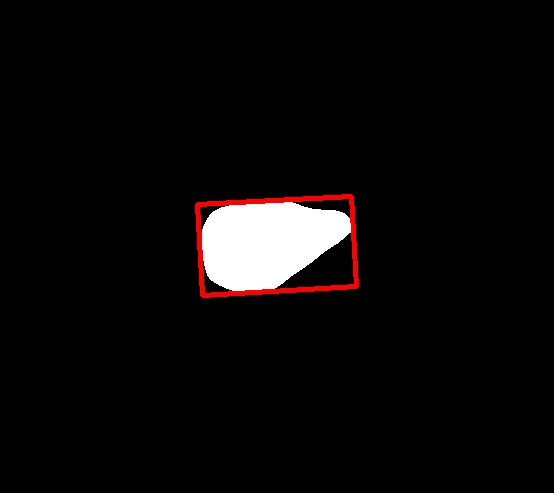

Supplement: Supplementary 1 — File S1 [file plantphenomics.0158.f1.zip › supplementary/0721_004_103.jpg]

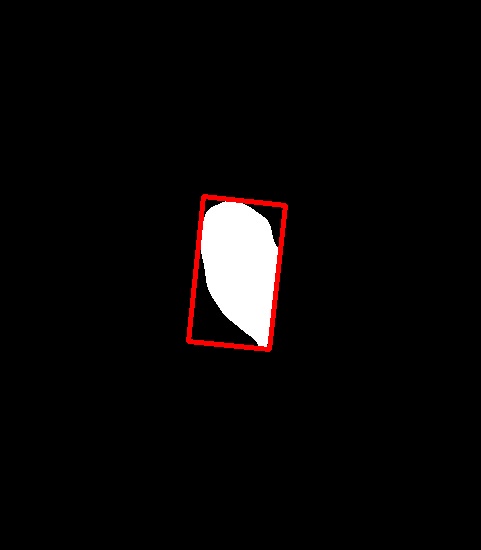

Supplement: Supplementary 1 — File S1 [file plantphenomics.0158.f1.zip › supplementary/0721_004_116.jpg]

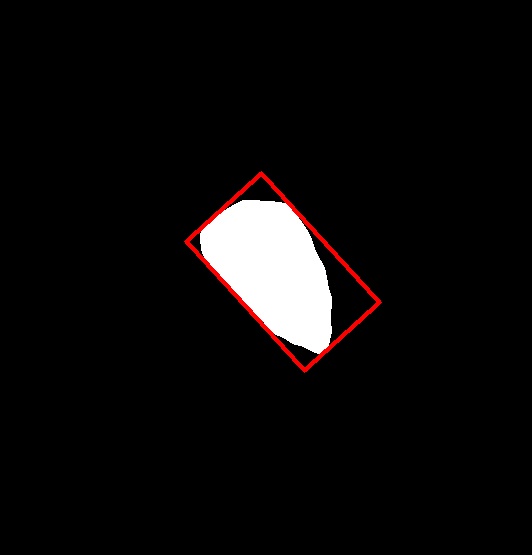

Supplement: Supplementary 1 — File S1 [file plantphenomics.0158.f1.zip › supplementary/0721_004_34.jpg]

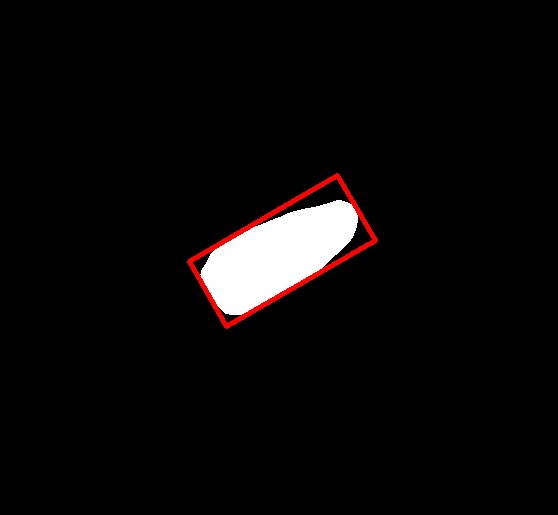

Supplement: Supplementary 1 — File S1 [file plantphenomics.0158.f1.zip › supplementary/0721_004_68.jpg]

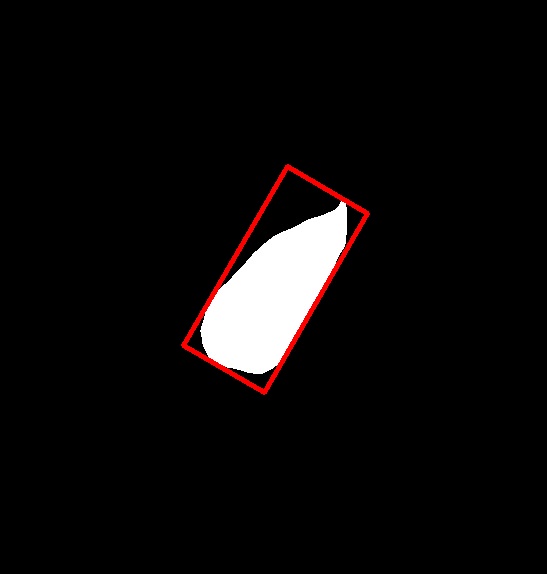

Supplement: Supplementary 1 — File S1 [file plantphenomics.0158.f1.zip › supplementary/0721_004_97.jpg]

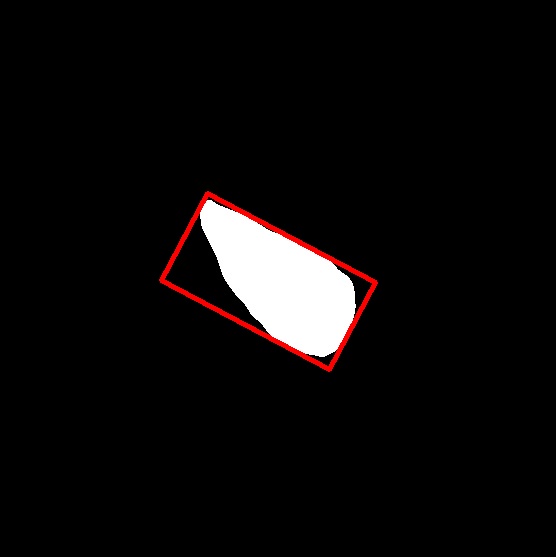

Supplement: Supplementary 1 — File S1 [file plantphenomics.0158.f1.zip › supplementary/0721_005_0.jpg]

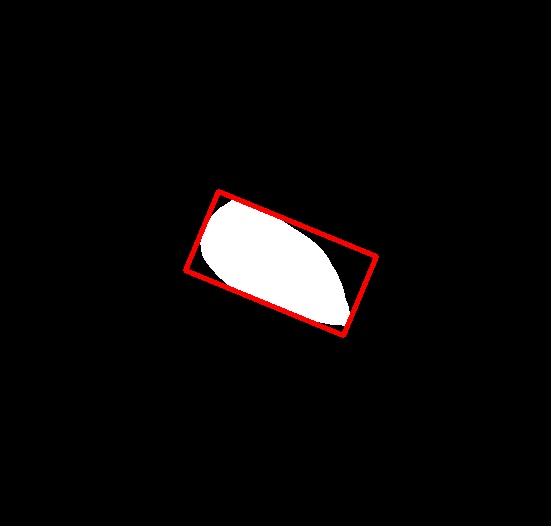

Supplement: Supplementary 1 — File S1 [file plantphenomics.0158.f1.zip › supplementary/0721_005_147.jpg]

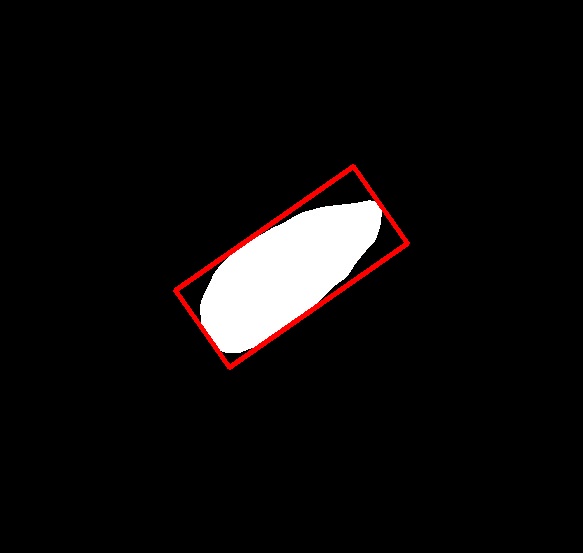

Supplement: Supplementary 1 — File S1 [file plantphenomics.0158.f1.zip › supplementary/0721_005_176.jpg]

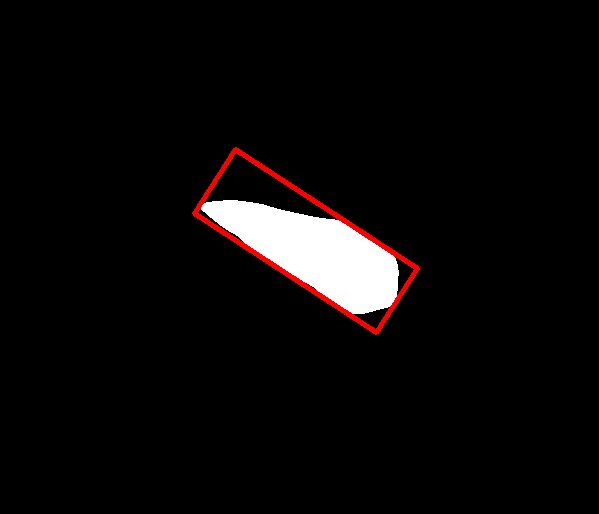

Supplement: Supplementary 1 — File S1 [file plantphenomics.0158.f1.zip › supplementary/0721_005_57.jpg]

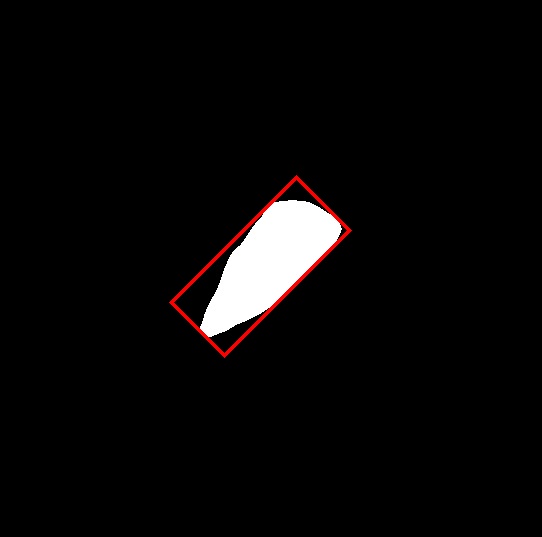

Supplement: Supplementary 1 — File S1 [file plantphenomics.0158.f1.zip › supplementary/0721_005_80.jpg]

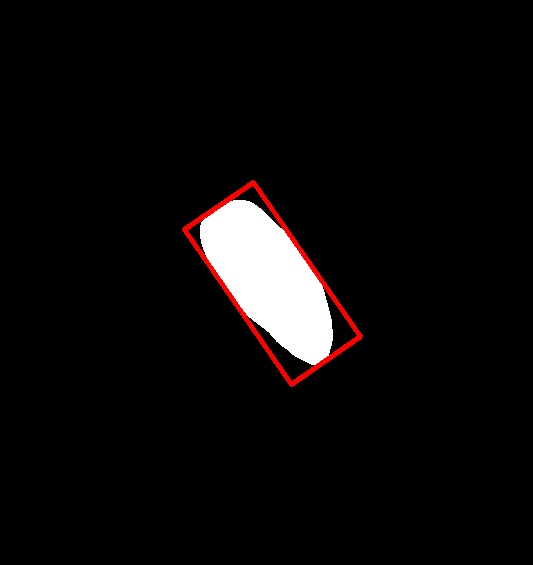

Supplement: Supplementary 1 — File S1 [file plantphenomics.0158.f1.zip › supplementary/0721_006_0.jpg]

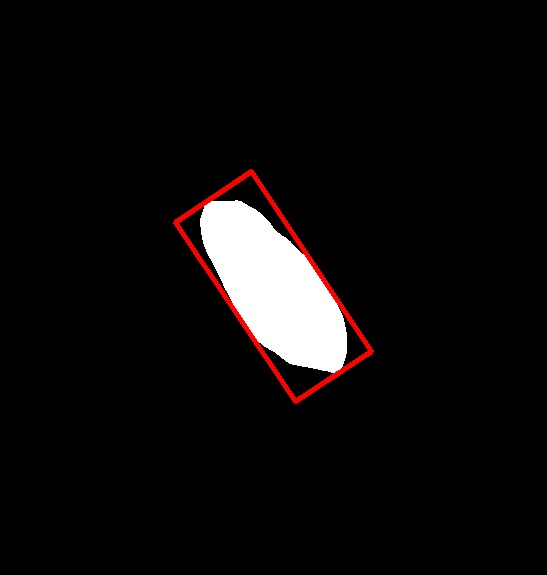

Supplement: Supplementary 1 — File S1 [file plantphenomics.0158.f1.zip › supplementary/0721_006_2.jpg]

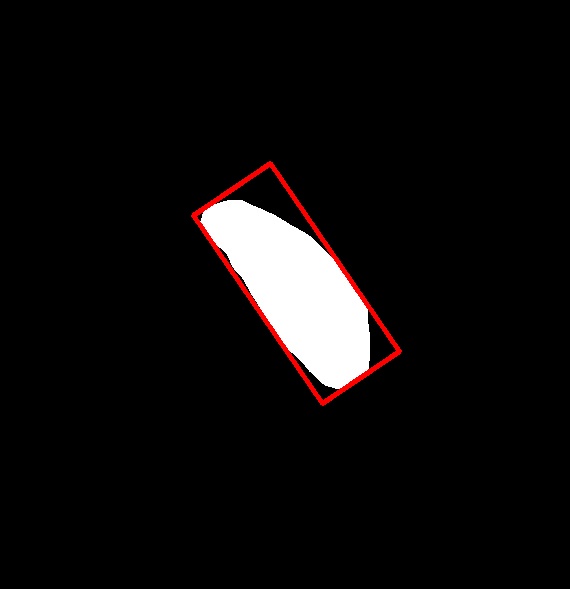

Supplement: Supplementary 1 — File S1 [file plantphenomics.0158.f1.zip › supplementary/0721_006_35.jpg]

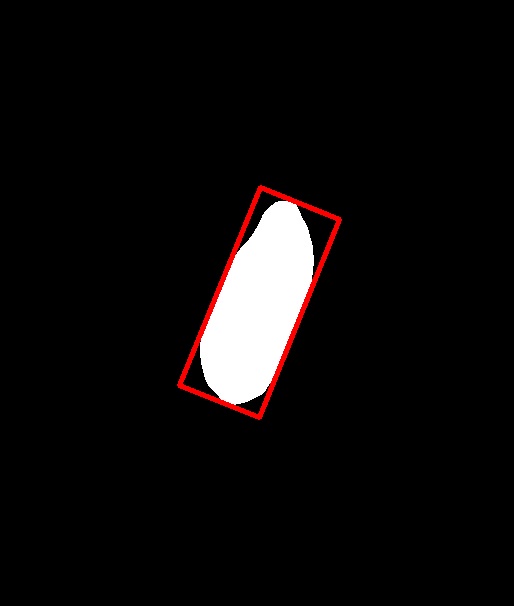

Supplement: Supplementary 1 — File S1 [file plantphenomics.0158.f1.zip › supplementary/0721_006_55.jpg]

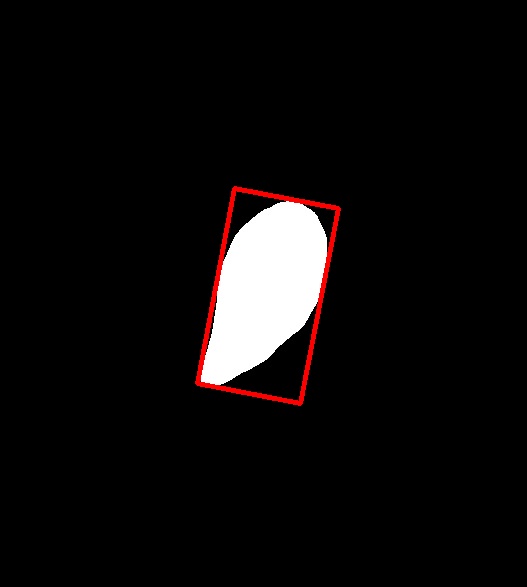

Supplement: Supplementary 1 — File S1 [file plantphenomics.0158.f1.zip › supplementary/0721_006_68.jpg]

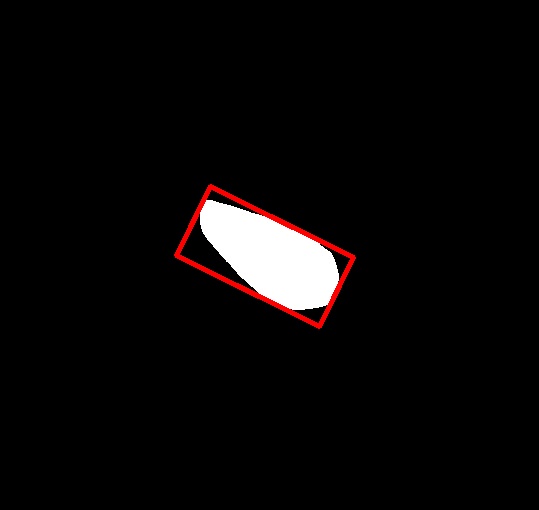

Supplement: Supplementary 1 — File S1 [file plantphenomics.0158.f1.zip › supplementary/0721_007_2.jpg]

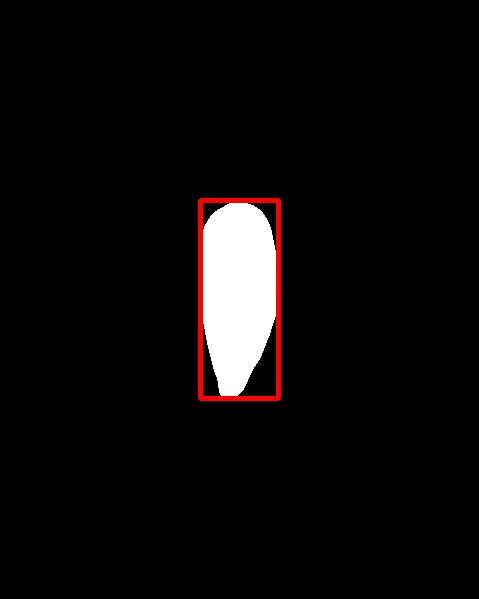

Supplement: Supplementary 1 — File S1 [file plantphenomics.0158.f1.zip › supplementary/0721_007_24.jpg]

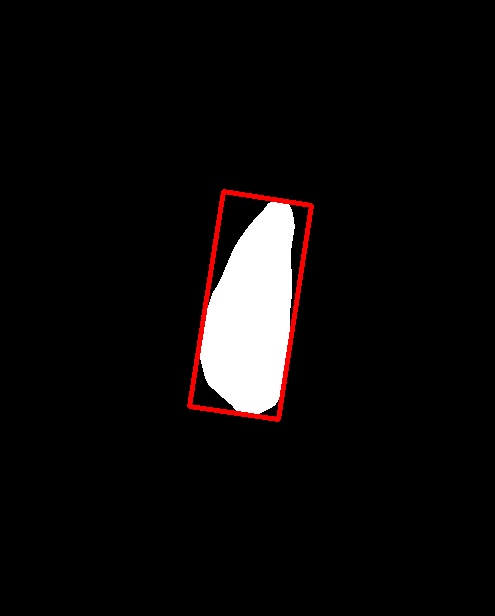

Supplement: Supplementary 1 — File S1 [file plantphenomics.0158.f1.zip › supplementary/0721_007_35.jpg]

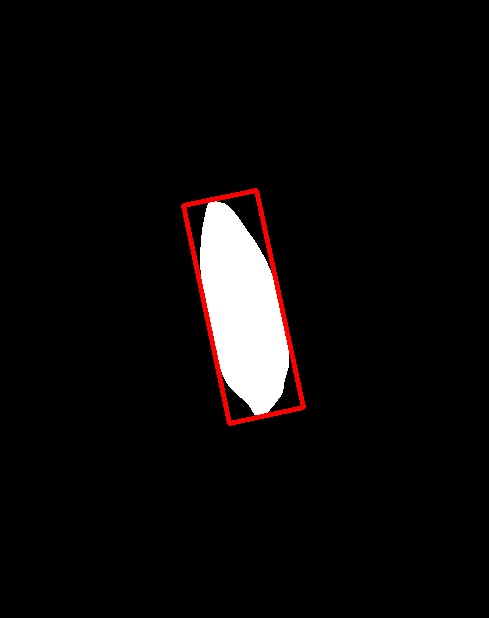

Supplement: Supplementary 1 — File S1 [file plantphenomics.0158.f1.zip › supplementary/0721_007_79.jpg]

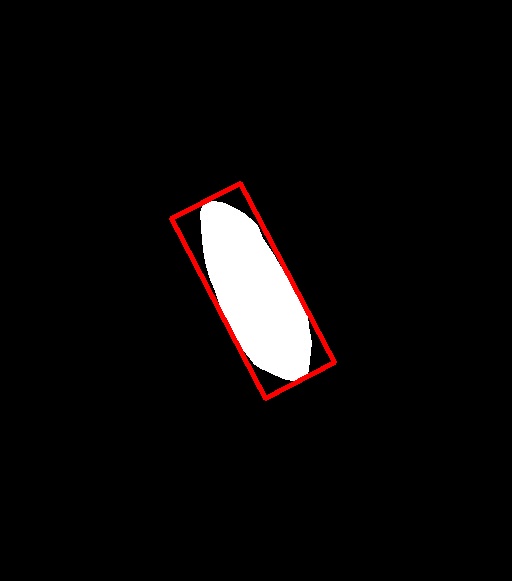

Supplement: Supplementary 1 — File S1 [file plantphenomics.0158.f1.zip › supplementary/0721_007_90.jpg]

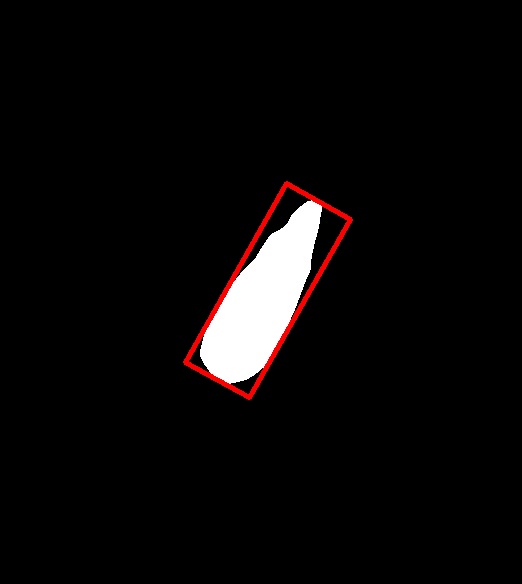

Supplement: Supplementary 1 — File S1 [file plantphenomics.0158.f1.zip › supplementary/0721_008_11.jpg]

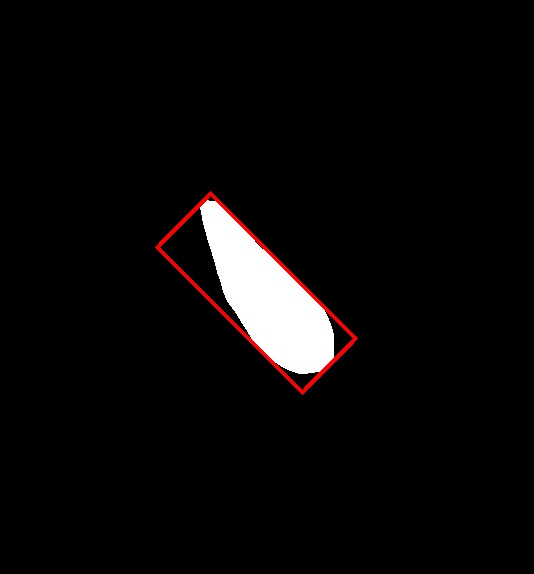

Supplement: Supplementary 1 — File S1 [file plantphenomics.0158.f1.zip › supplementary/0721_008_13.jpg]

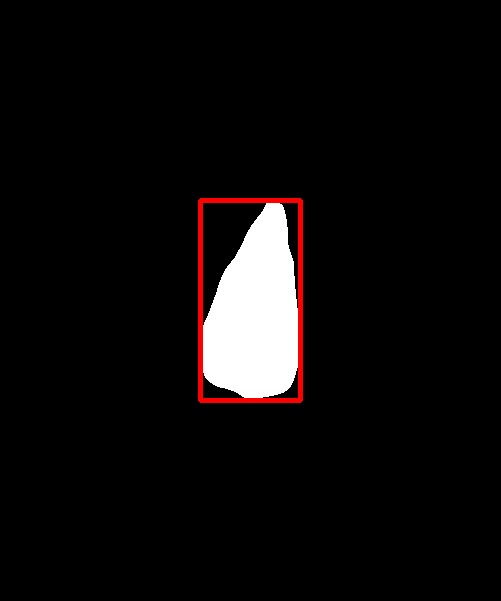

Supplement: Supplementary 1 — File S1 [file plantphenomics.0158.f1.zip › supplementary/0721_008_142.jpg]

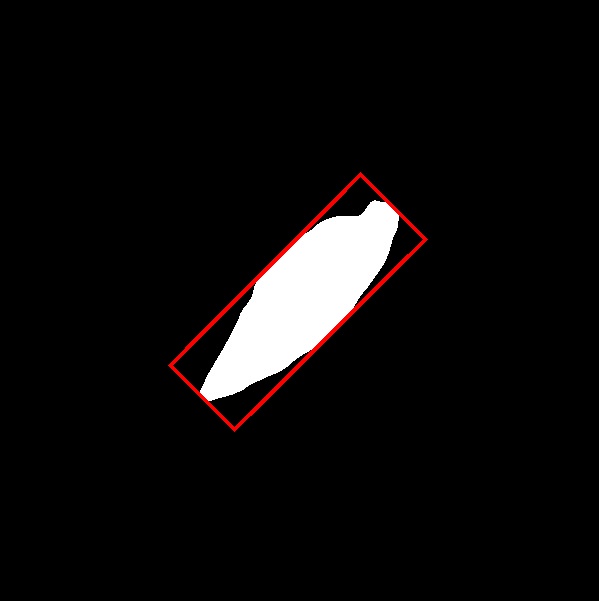

Supplement: Supplementary 1 — File S1 [file plantphenomics.0158.f1.zip › supplementary/0721_008_149.jpg]

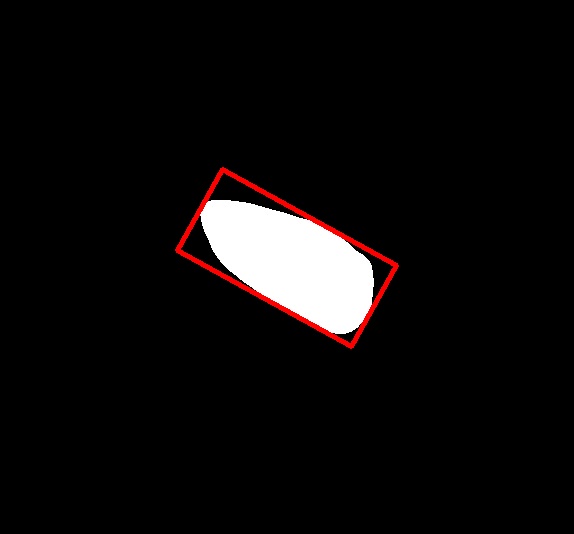

Supplement: Supplementary 1 — File S1 [file plantphenomics.0158.f1.zip › supplementary/0721_008_55.jpg]

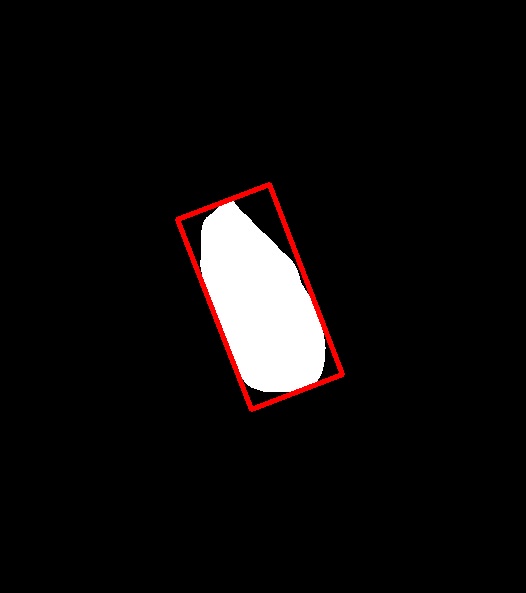

Supplement: Supplementary 1 — File S1 [file plantphenomics.0158.f1.zip › supplementary/0721_009_166.jpg]

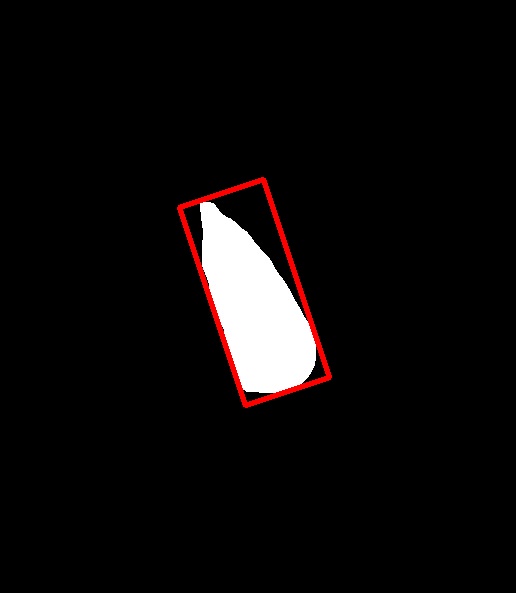

Supplement: Supplementary 1 — File S1 [file plantphenomics.0158.f1.zip › supplementary/0721_009_19.jpg]

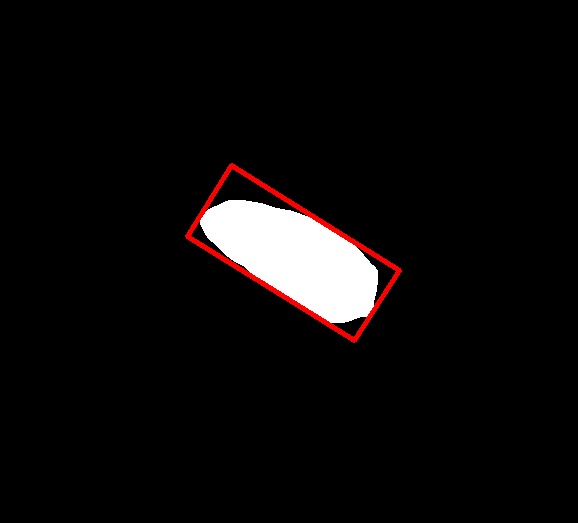

Supplement: Supplementary 1 — File S1 [file plantphenomics.0158.f1.zip › supplementary/0721_009_42.jpg]

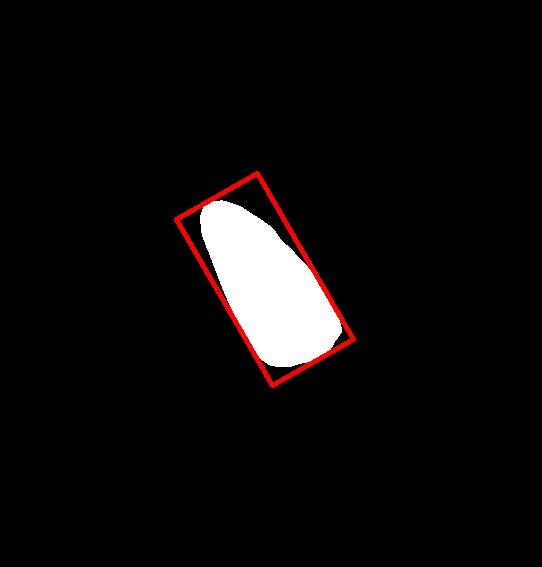

Supplement: Supplementary 1 — File S1 [file plantphenomics.0158.f1.zip › supplementary/0721_009_89.jpg]

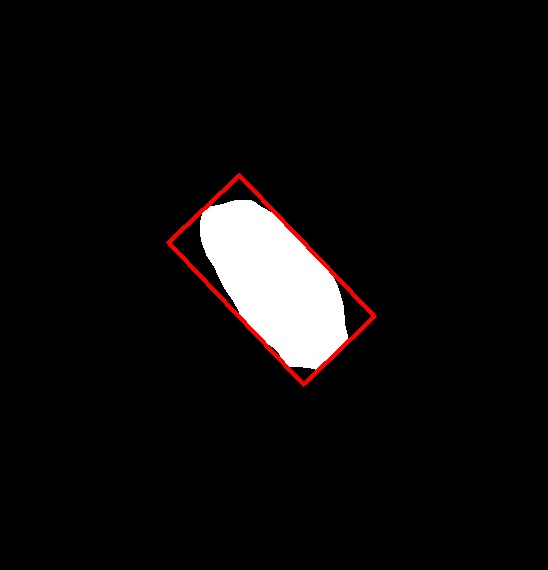

Supplement: Supplementary 1 — File S1 [file plantphenomics.0158.f1.zip › supplementary/0721_009_99.jpg]

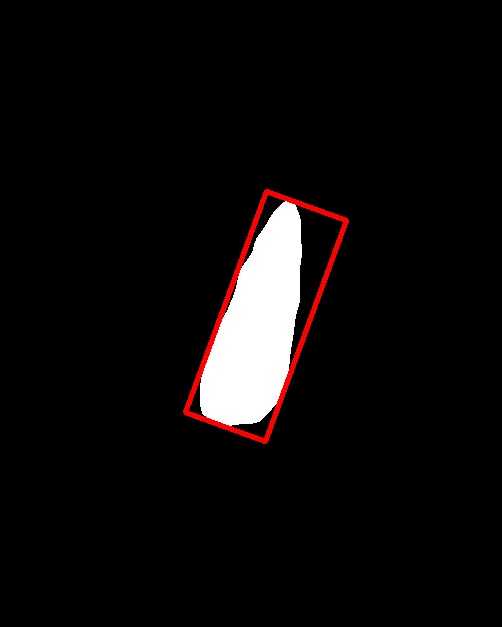

Supplement: Supplementary 1 — File S1 [file plantphenomics.0158.f1.zip › supplementary/0721_010_101.jpg]

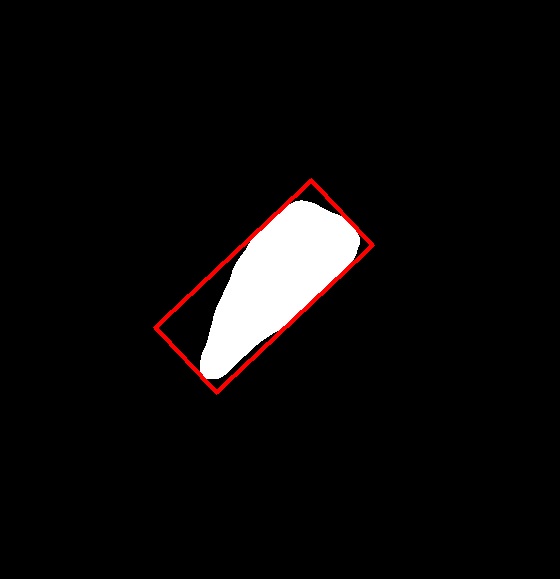

Supplement: Supplementary 1 — File S1 [file plantphenomics.0158.f1.zip › supplementary/0721_010_141.jpg]

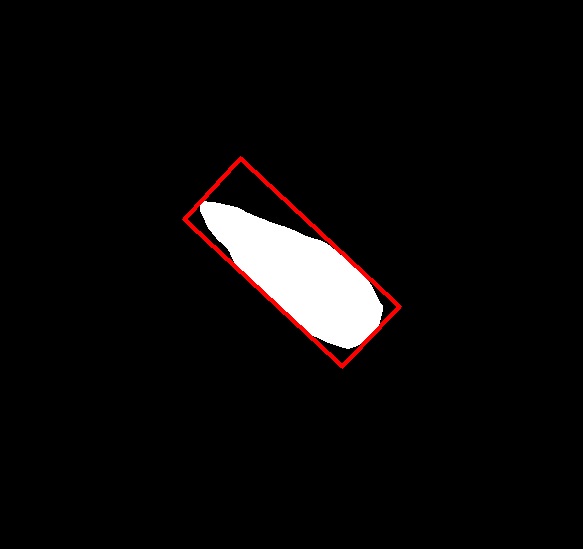

Supplement: Supplementary 1 — File S1 [file plantphenomics.0158.f1.zip › supplementary/0721_010_174.jpg]

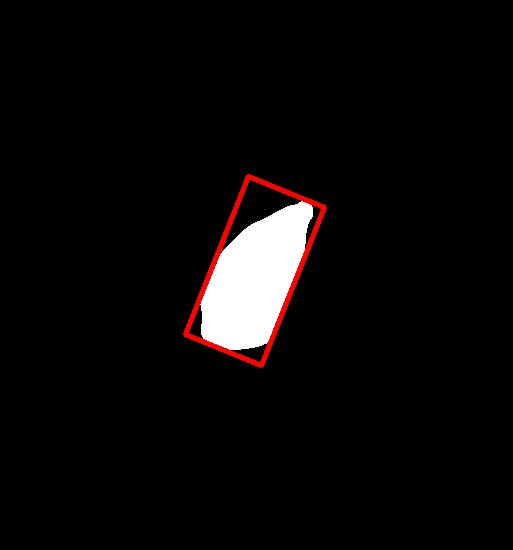

Supplement: Supplementary 1 — File S1 [file plantphenomics.0158.f1.zip › supplementary/0721_010_31.jpg]

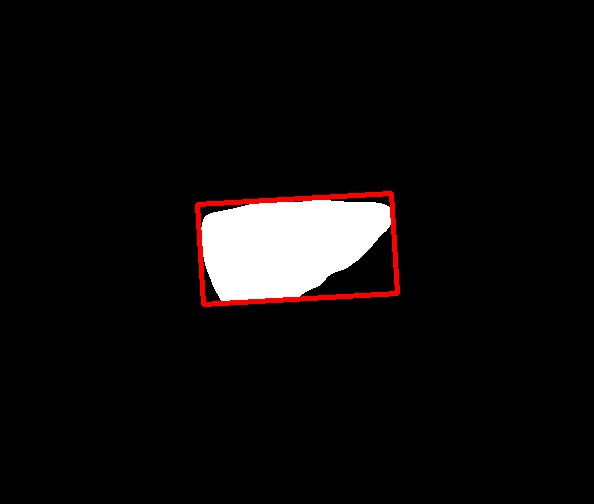

Supplement: Supplementary 1 — File S1 [file plantphenomics.0158.f1.zip › supplementary/0721_010_34.jpg]

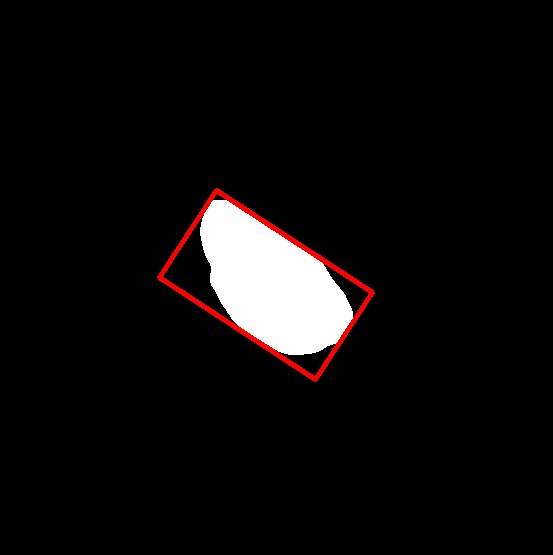

Supplement: Supplementary 1 — File S1 [file plantphenomics.0158.f1.zip › supplementary/0721_011_110.jpg]

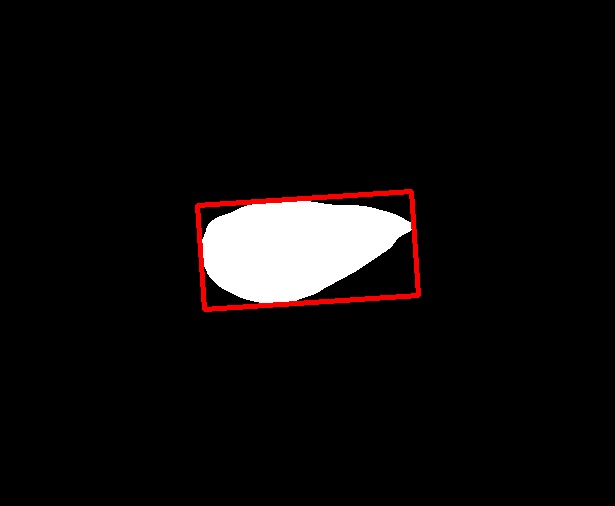

Supplement: Supplementary 1 — File S1 [file plantphenomics.0158.f1.zip › supplementary/0721_011_13.jpg]

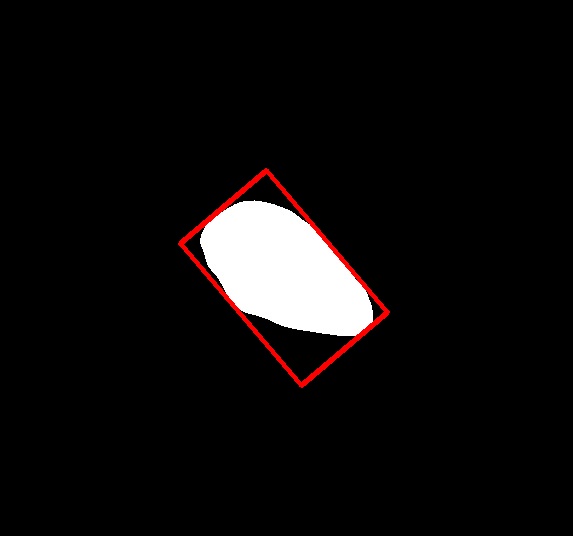

Supplement: Supplementary 1 — File S1 [file plantphenomics.0158.f1.zip › supplementary/0721_011_22.jpg]

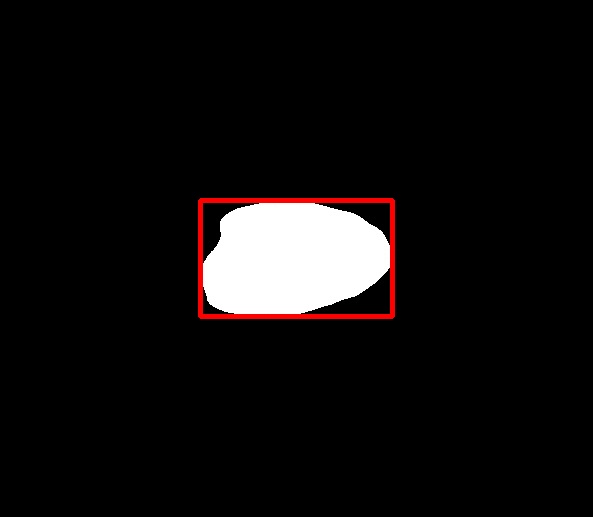

Supplement: Supplementary 1 — File S1 [file plantphenomics.0158.f1.zip › supplementary/0721_011_46.jpg]

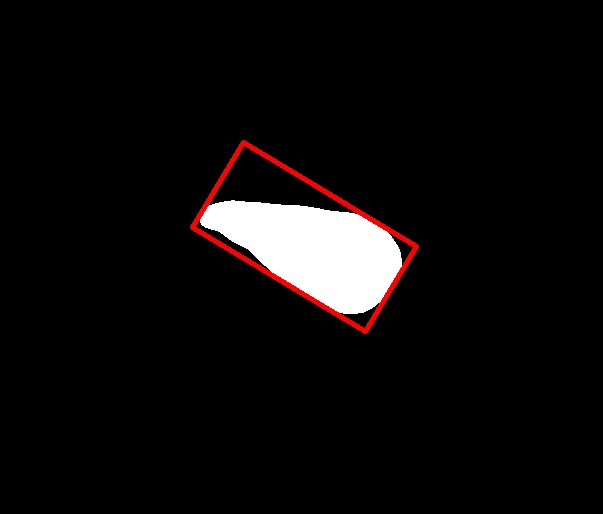

Supplement: Supplementary 1 — File S1 [file plantphenomics.0158.f1.zip › supplementary/0721_011_52.jpg]

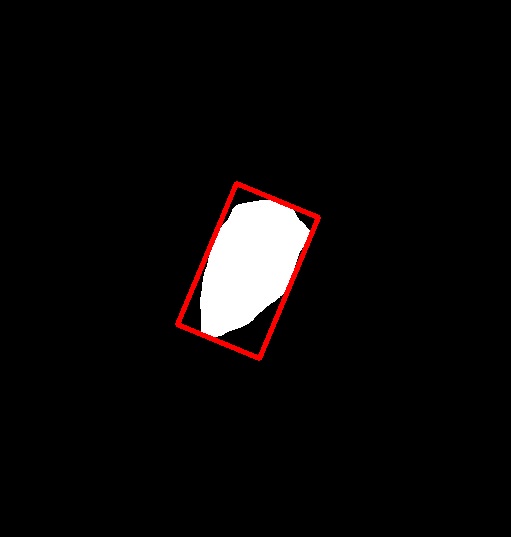

Supplement: Supplementary 1 — File S1 [file plantphenomics.0158.f1.zip › supplementary/0721_012_103.jpg]

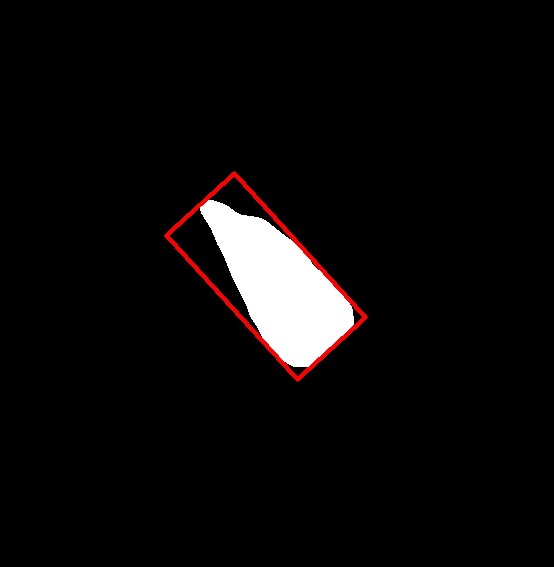

Supplement: Supplementary 1 — File S1 [file plantphenomics.0158.f1.zip › supplementary/0721_012_11.jpg]

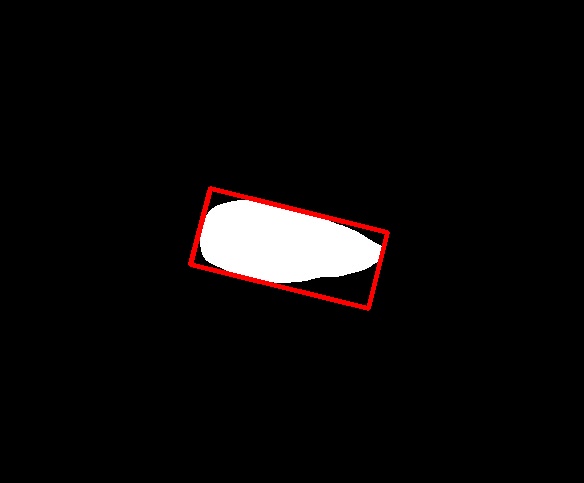

Supplement: Supplementary 1 — File S1 [file plantphenomics.0158.f1.zip › supplementary/0721_012_113.jpg]

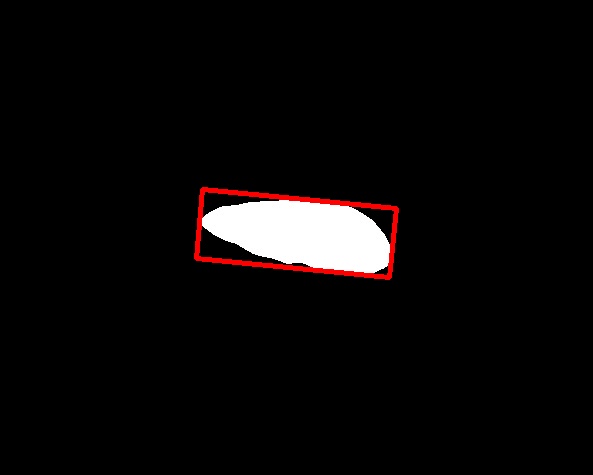

Supplement: Supplementary 1 — File S1 [file plantphenomics.0158.f1.zip › supplementary/0721_012_24.jpg]

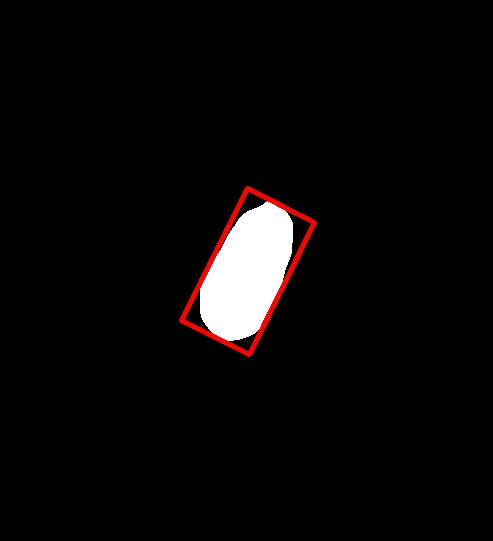

Supplement: Supplementary 1 — File S1 [file plantphenomics.0158.f1.zip › supplementary/0721_012_35.jpg]

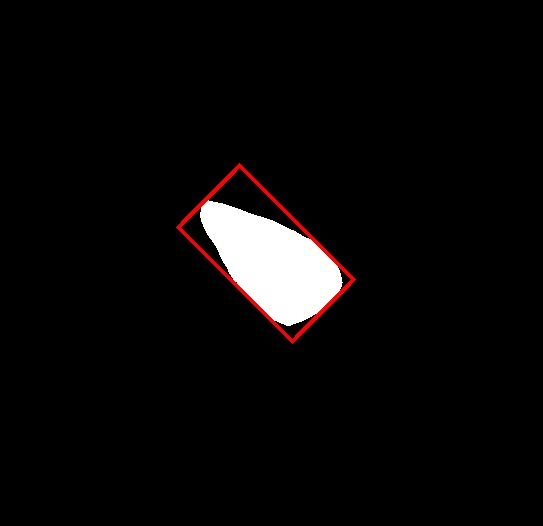

Supplement: Supplementary 1 — File S1 [file plantphenomics.0158.f1.zip › supplementary/0721_013_0.jpg]

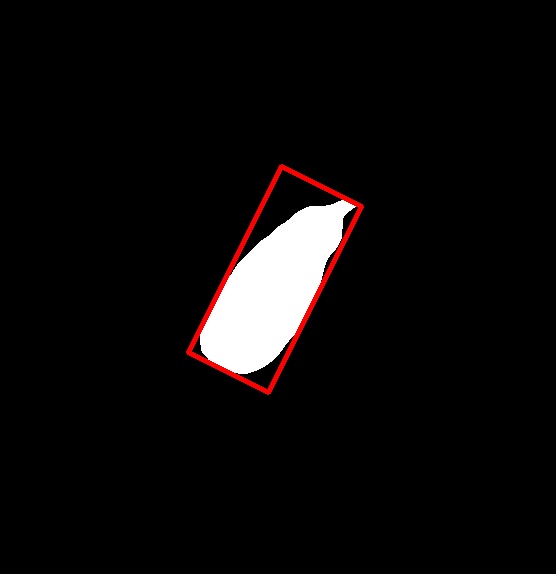

Supplement: Supplementary 1 — File S1 [file plantphenomics.0158.f1.zip › supplementary/0721_013_13.jpg]

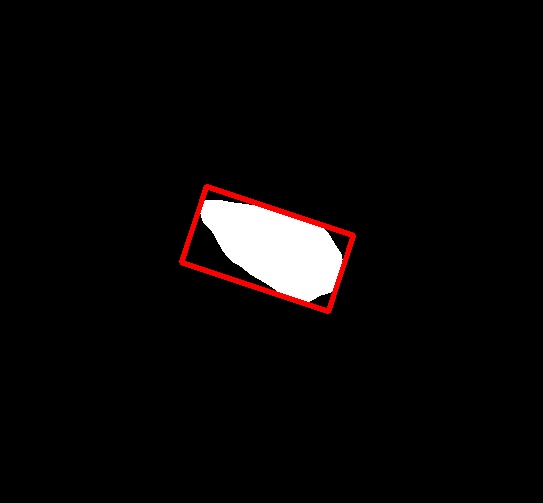

Supplement: Supplementary 1 — File S1 [file plantphenomics.0158.f1.zip › supplementary/0721_013_15.jpg]

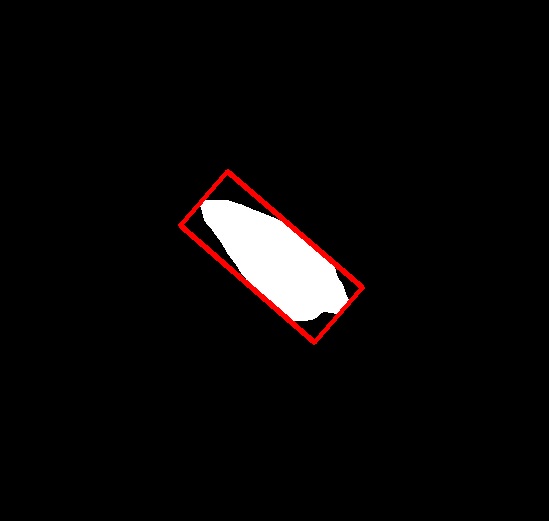

Supplement: Supplementary 1 — File S1 [file plantphenomics.0158.f1.zip › supplementary/0721_013_18.jpg]

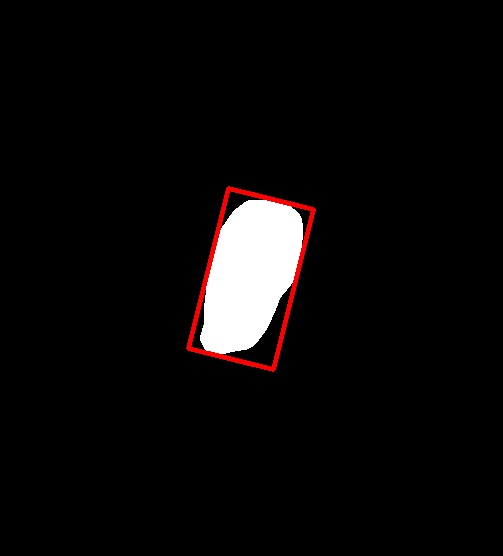

Supplement: Supplementary 1 — File S1 [file plantphenomics.0158.f1.zip › supplementary/0721_013_21.jpg]

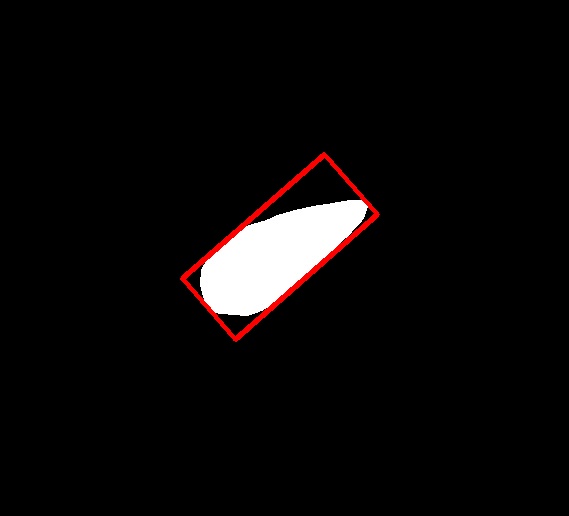

Supplement: Supplementary 1 — File S1 [file plantphenomics.0158.f1.zip › supplementary/0721_014_105.jpg]

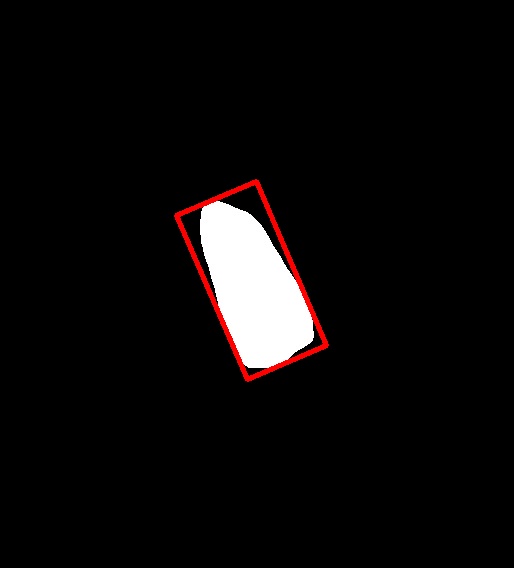

Supplement: Supplementary 1 — File S1 [file plantphenomics.0158.f1.zip › supplementary/0721_014_112.jpg]

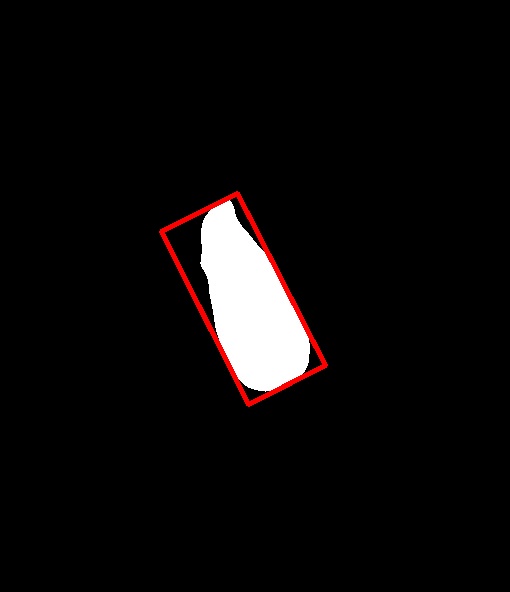

Supplement: Supplementary 1 — File S1 [file plantphenomics.0158.f1.zip › supplementary/0721_014_130.jpg]

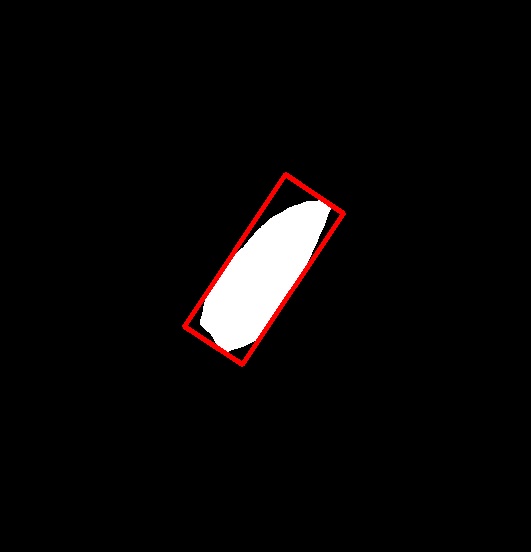

Supplement: Supplementary 1 — File S1 [file plantphenomics.0158.f1.zip › supplementary/0721_014_40.jpg]

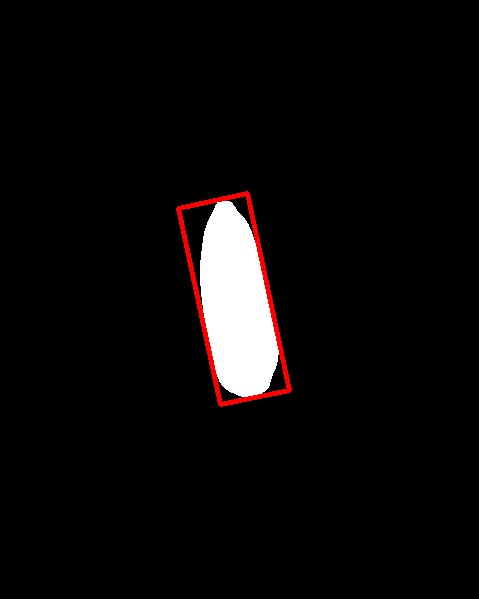

Supplement: Supplementary 1 — File S1 [file plantphenomics.0158.f1.zip › supplementary/0721_014_83.jpg]

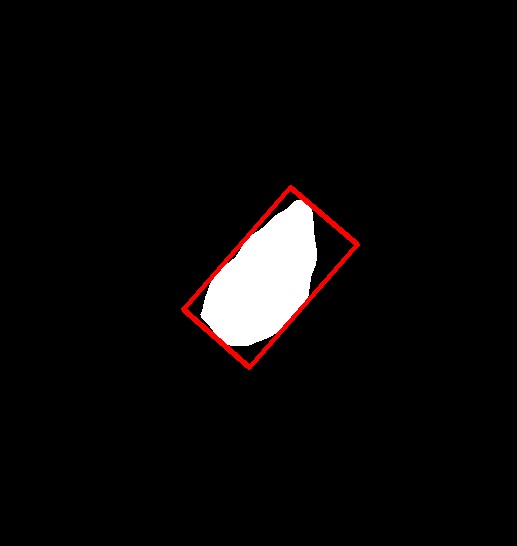

Supplement: Supplementary 1 — File S1 [file plantphenomics.0158.f1.zip › supplementary/0721_015_107.jpg]

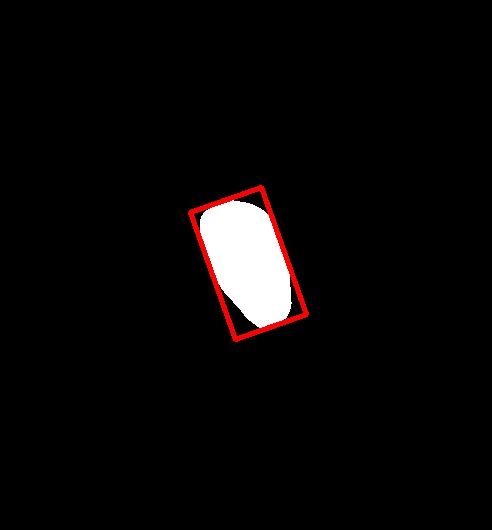

Supplement: Supplementary 1 — File S1 [file plantphenomics.0158.f1.zip › supplementary/0721_015_130.jpg]

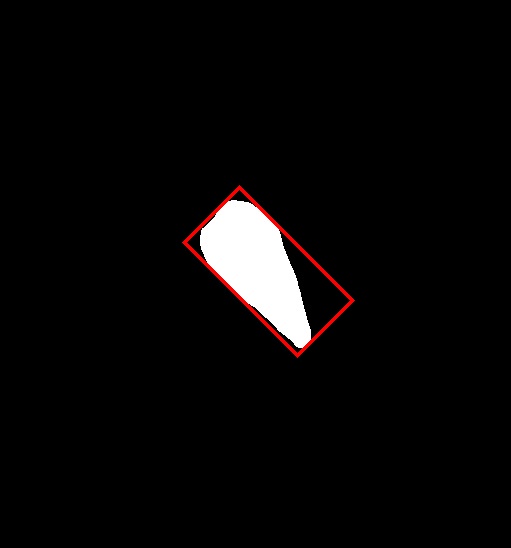

Supplement: Supplementary 1 — File S1 [file plantphenomics.0158.f1.zip › supplementary/0721_015_45.jpg]

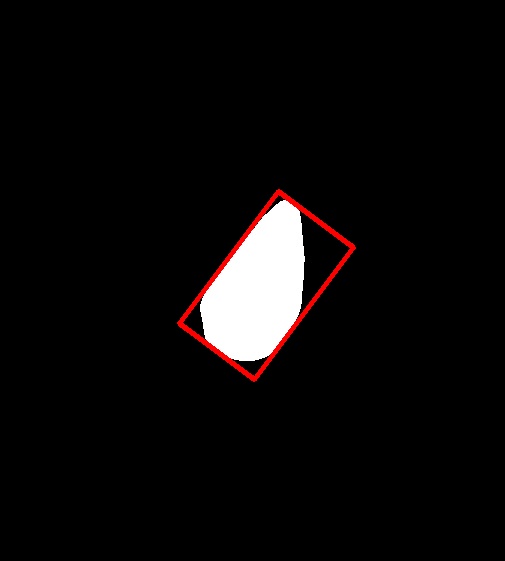

Supplement: Supplementary 1 — File S1 [file plantphenomics.0158.f1.zip › supplementary/0721_015_75.jpg]

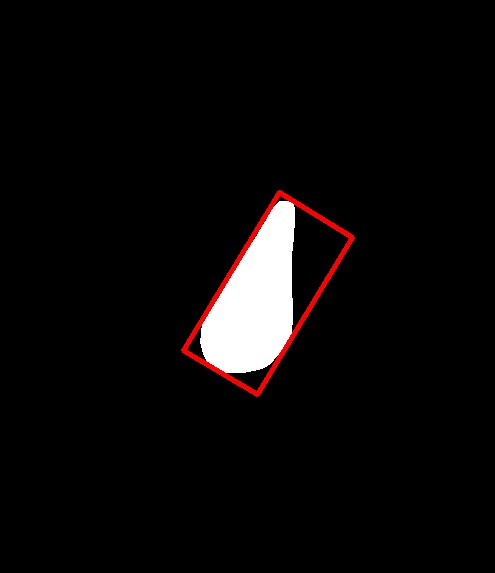

Supplement: Supplementary 1 — File S1 [file plantphenomics.0158.f1.zip › supplementary/0721_015_86.jpg]

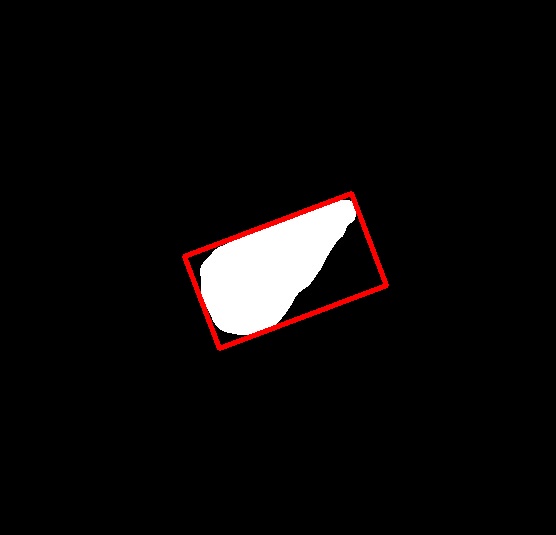

Supplement: Supplementary 1 — File S1 [file plantphenomics.0158.f1.zip › supplementary/0721_016_120.jpg]

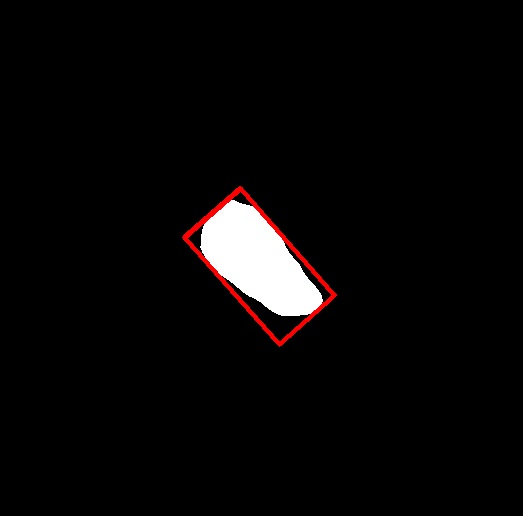

Supplement: Supplementary 1 — File S1 [file plantphenomics.0158.f1.zip › supplementary/0721_016_15.jpg]

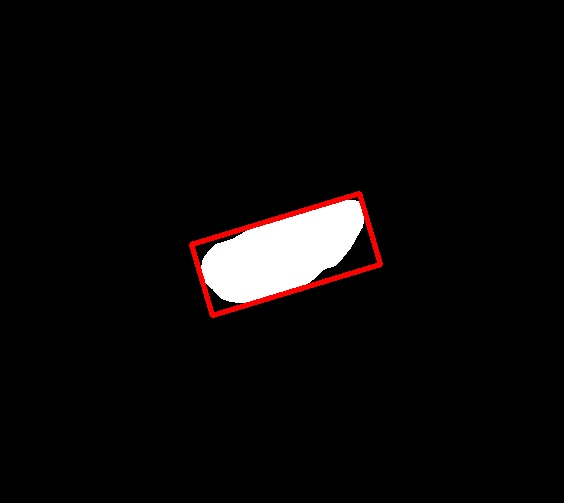

Supplement: Supplementary 1 — File S1 [file plantphenomics.0158.f1.zip › supplementary/0721_016_19.jpg]

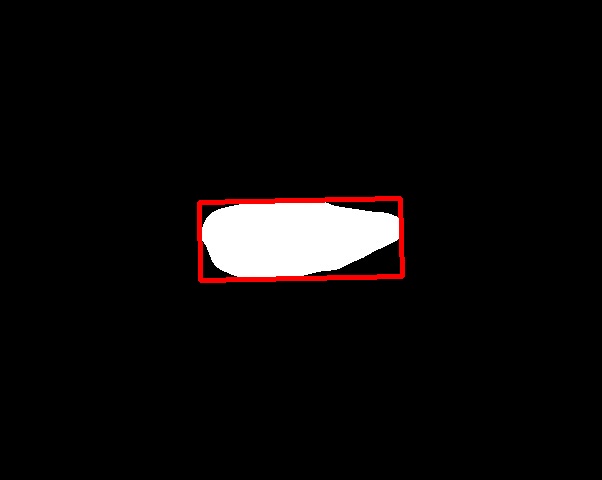

Supplement: Supplementary 1 — File S1 [file plantphenomics.0158.f1.zip › supplementary/0721_016_57.jpg]

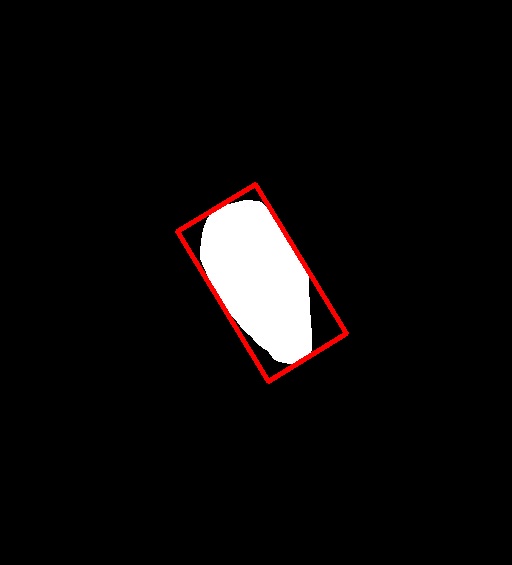

Supplement: Supplementary 1 — File S1 [file plantphenomics.0158.f1.zip › supplementary/0721_016_61.jpg]

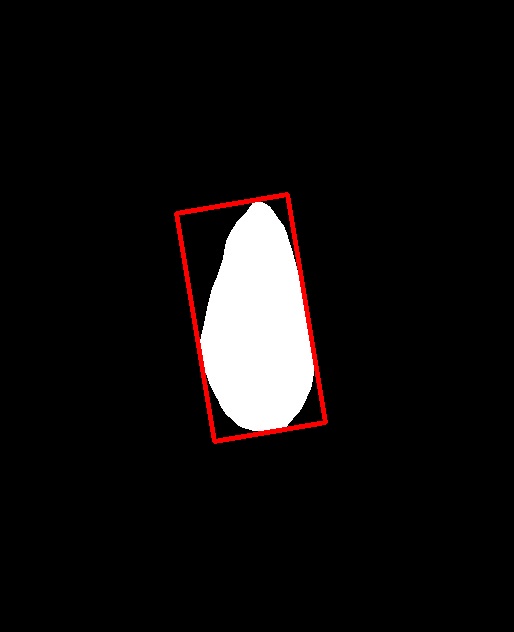

Supplement: Supplementary 1 — File S1 [file plantphenomics.0158.f1.zip › supplementary/0721_017_1.jpg]

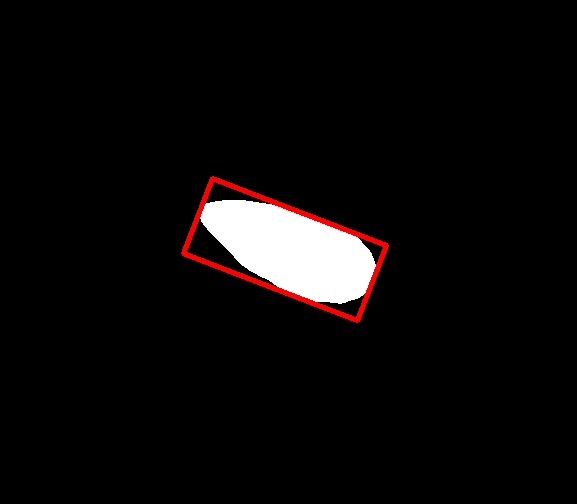

Supplement: Supplementary 1 — File S1 [file plantphenomics.0158.f1.zip › supplementary/0721_017_11.jpg]

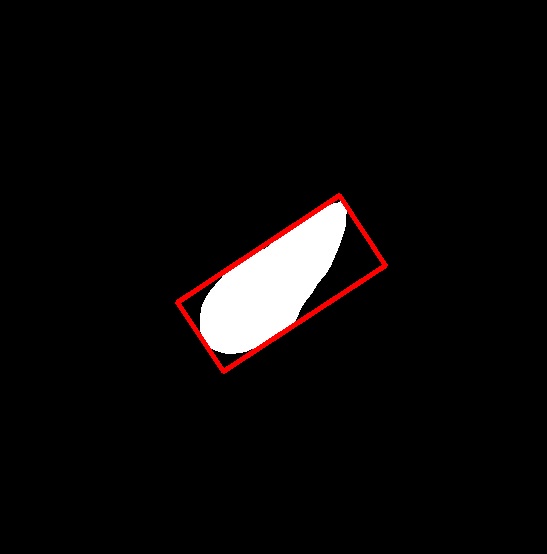

Supplement: Supplementary 1 — File S1 [file plantphenomics.0158.f1.zip › supplementary/0721_017_13.jpg]

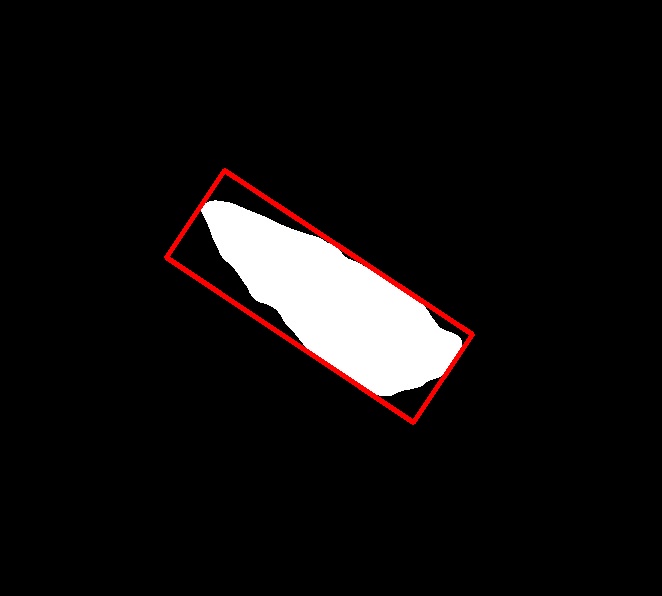

Supplement: Supplementary 1 — File S1 [file plantphenomics.0158.f1.zip › supplementary/0721_017_138.jpg]

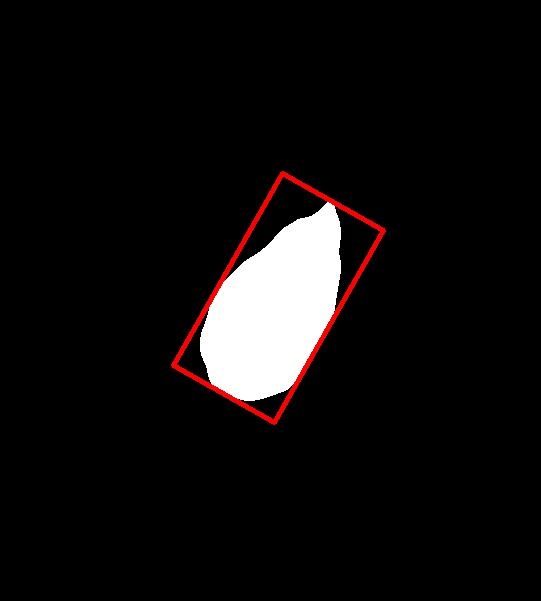

Supplement: Supplementary 1 — File S1 [file plantphenomics.0158.f1.zip › supplementary/0721_017_19.jpg]

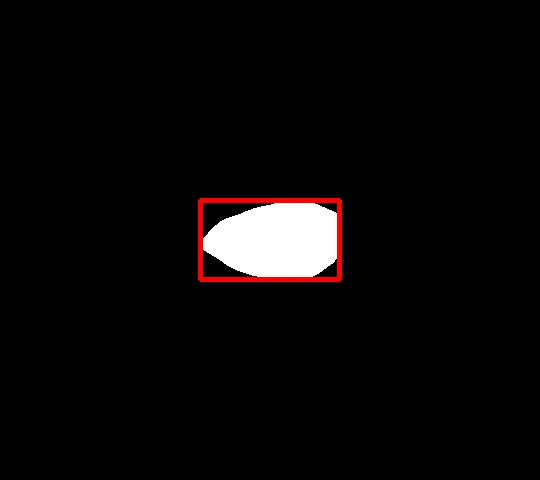

Supplement: Supplementary 1 — File S1 [file plantphenomics.0158.f1.zip › supplementary/0721_018_10.jpg]

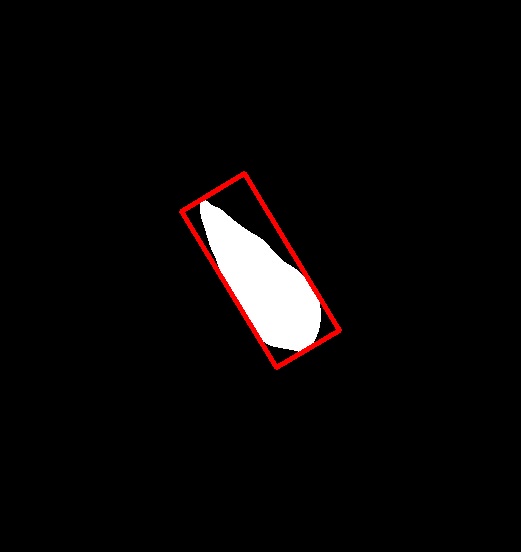

Supplement: Supplementary 1 — File S1 [file plantphenomics.0158.f1.zip › supplementary/0721_018_130.jpg]

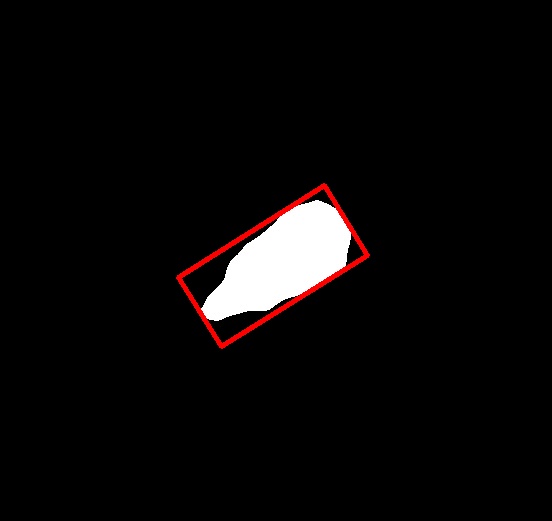

Supplement: Supplementary 1 — File S1 [file plantphenomics.0158.f1.zip › supplementary/0721_018_18.jpg]

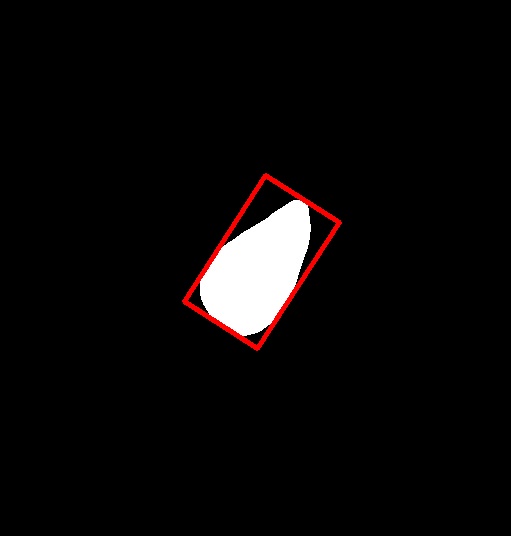

Supplement: Supplementary 1 — File S1 [file plantphenomics.0158.f1.zip › supplementary/0721_018_24.jpg]

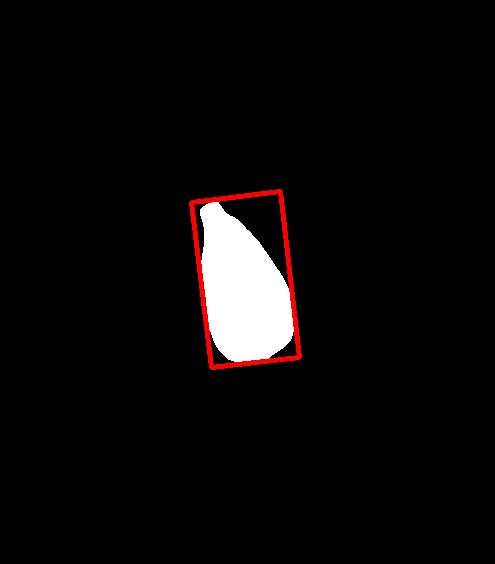

Supplement: Supplementary 1 — File S1 [file plantphenomics.0158.f1.zip › supplementary/0721_018_84.jpg]

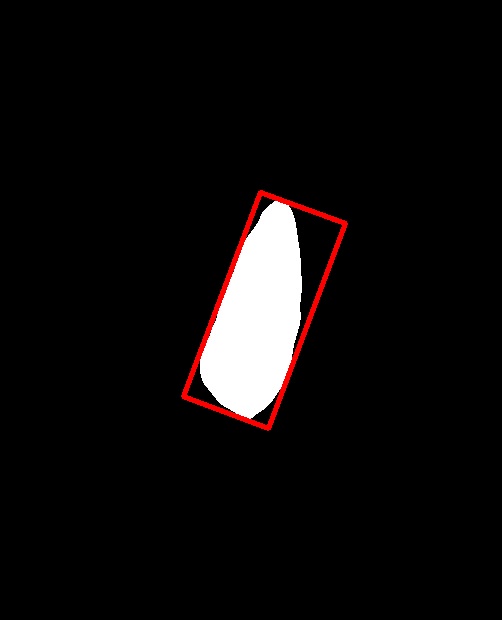

Supplement: Supplementary 1 — File S1 [file plantphenomics.0158.f1.zip › supplementary/0721_019_114.jpg]

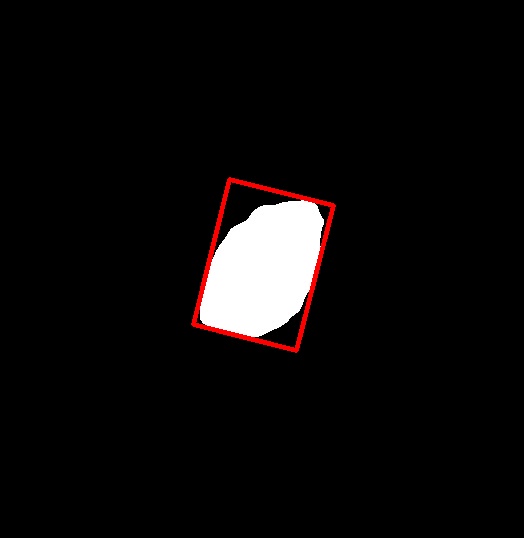

Supplement: Supplementary 1 — File S1 [file plantphenomics.0158.f1.zip › supplementary/0721_019_132.jpg]

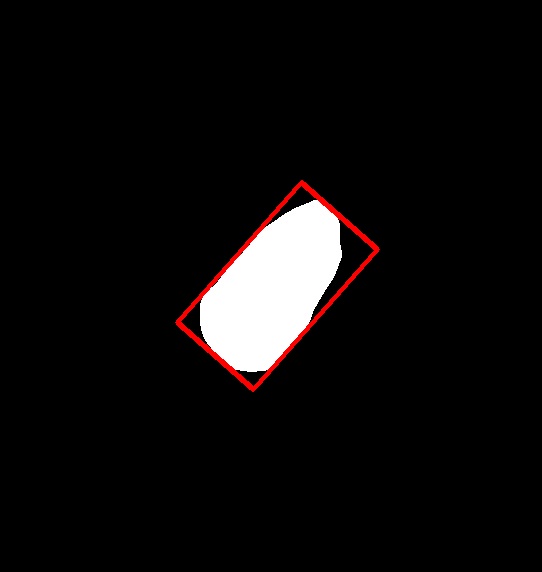

Supplement: Supplementary 1 — File S1 [file plantphenomics.0158.f1.zip › supplementary/0721_019_138.jpg]

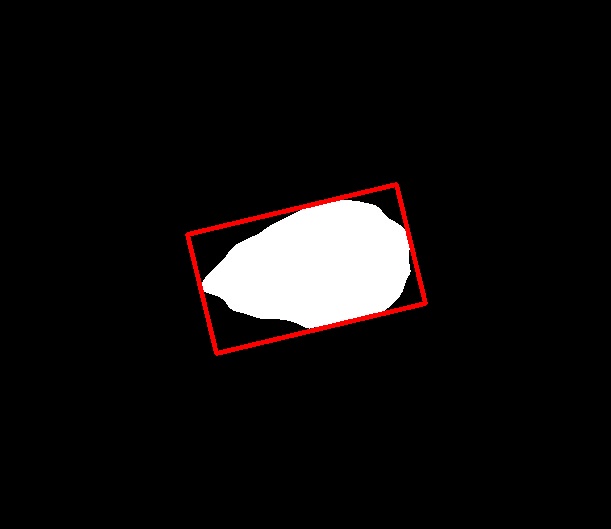

Supplement: Supplementary 1 — File S1 [file plantphenomics.0158.f1.zip › supplementary/0721_019_156.jpg]

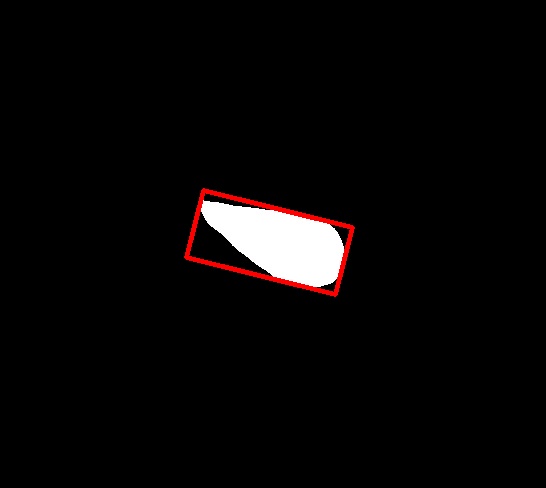

Supplement: Supplementary 1 — File S1 [file plantphenomics.0158.f1.zip › supplementary/0721_019_160.jpg]

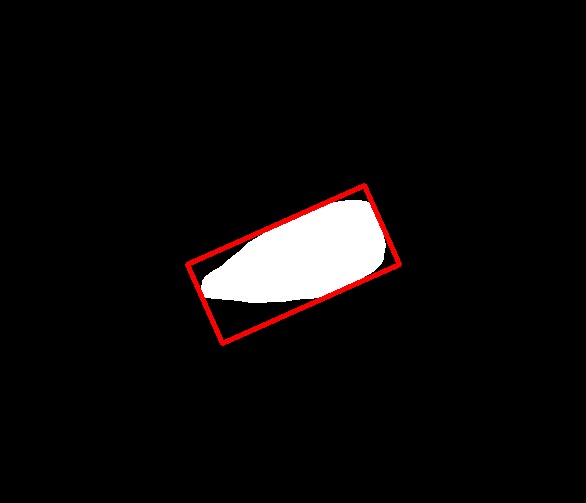

Supplement: Supplementary 1 — File S1 [file plantphenomics.0158.f1.zip › supplementary/0721_020_121.jpg]

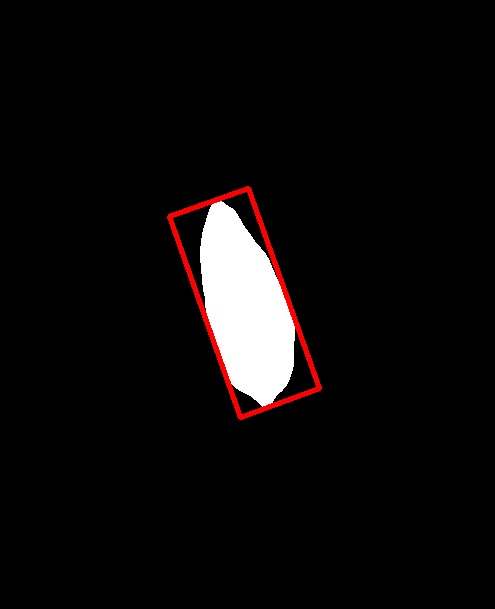

Supplement: Supplementary 1 — File S1 [file plantphenomics.0158.f1.zip › supplementary/0721_020_130.jpg]

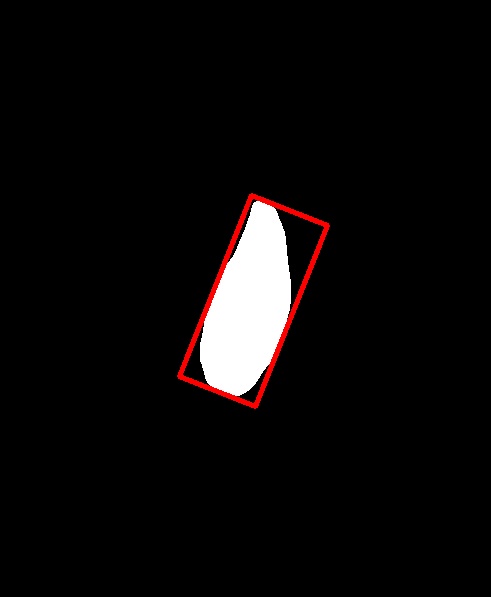

Supplement: Supplementary 1 — File S1 [file plantphenomics.0158.f1.zip › supplementary/0721_020_136.jpg]

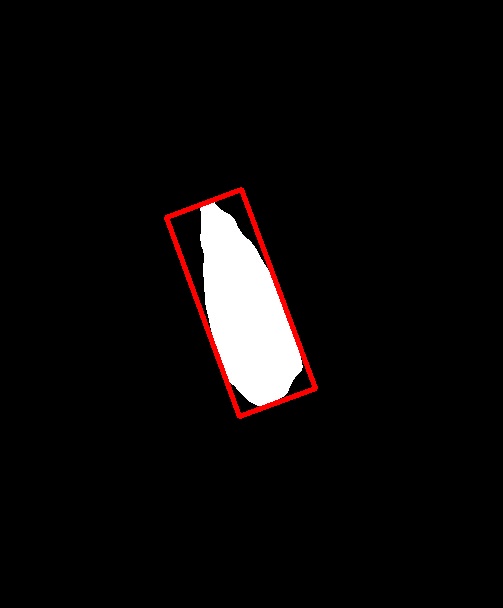

Supplement: Supplementary 1 — File S1 [file plantphenomics.0158.f1.zip › supplementary/0721_020_2.jpg]

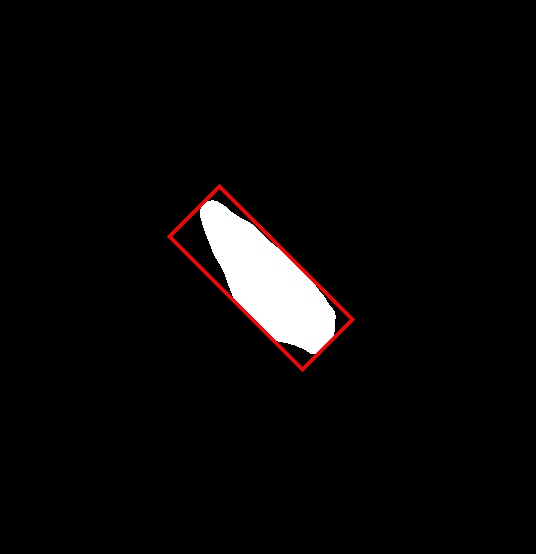

Supplement: Supplementary 1 — File S1 [file plantphenomics.0158.f1.zip › supplementary/0721_020_33.jpg]
